# Diversity and Distribution of *Colletotrichum* Species Causing Anthracnose in China

**DOI:** 10.3390/jof11110781

**Published:** 2025-10-30

**Authors:** Weishan Zhang, Xinlei Fan

**Affiliations:** 1 The Key Laboratory of Efficient Production of Forest Resources, Beijing Forestry University, Beijing 100083, China; weishanzhang@bjfu.edu.cn; 2 The Key Laboratory for Silviculture and Conservation of the Ministry of Education, Beijing Forestry University, Beijing 100083, China

**Keywords:** *Colletotrichum*, species diversity, taxonomic revision, walnut anthracnose

## Abstract

This study conducted a systematic investigation and identification of pathogenic fungi associated with anthracnose symptoms on economically important plants across multiple provinces in China (Beijing, Fujian, Guangdong, Guizhou, and Shaanxi). Through multi-locus phylogenetic analysis (ITS, *gapdh*, *chs-1*, *act*, *tub2*, *his3*, and *cal*) and morphological characterization of 67 strains, a total of 16 *Colletotrichum* species were identified, belonging to six species complexes (*C. acutatum*, *C. boninense*, *C. destructivum*, *C. gloeosporioides*, *C. orchidearum*, and *C. spaethianum*). Among these, four novel species were described: *Colletotrichum aquilariae*, *C. crataegi*, *C. dongguanense*, and *C. flavosporum*. The study also confirmed 12 known species: *C. boninense*, *C. fioriniae*, *C. fructicola*, *C. godetiae* (with *C. americanum* proposed as its synonym), *C. gloeosporioides* (with *C. juglandicola*, *C. juglandium*, and *C. peakense* proposed as its synonyms), *C. karsti*, *C. nymphaeae*, *C. orchidearum* (with *C. subplurivorum* proposed as its synonym), *C. plurivorum*, *C. siamense*, *C. sojae*, and *C. spaethianum*. The research revealed significant pathogen species diversity, distinct geographical distribution patterns (greatest diversity in Beijing, novel species primarily from Guangdong), and host preferences (e.g., *C. gloeosporioides* was the most widely distributed and dominant species on walnut). Furthermore, ten new host records were reported. The study explored correlations between pathogens and their hosts, particularly walnut, providing a crucial foundation for understanding the pathogen composition and ecology of anthracnose diseases affecting plants in China.

## 1. Introduction

*Colletotrichum* (Glomerellaceae) is distributed as one of the top ten global phytopathogenic genera worldwide [1]. *Colletotrichum* fungus grows and reproduces more rapidly in tropical and subtropical regions [2]. They can infect the aerial tissues of plants [3]. The typical symptoms are forming light to dark brown spots, which are usually round or irregular in shape [4,5]. In severe cases, they can merge into large areas, leading to extensive necrosis and wilting of plant leaves, stems, and other parts [6,7]. In addition, *Colletotrichum* is also capable of inducing post-harvest rot, which causes fruits and vegetables to emit a foul odor [8].

The morphological characteristics of *Colletotrichum* species exhibit variation and overlap. The conidia of the *C. acutatum* species complex are mostly long-elliptical or long-ovoid and relatively large [9]. The length-to-width ratio and the distinct scar at the base of the conidia of *C. boninense* show subtle differences compared to *C. gloeosporioides* [10,11]. The conidia of the *C. gloeosporioides* species complex are mostly long-elliptical or cylindrical and relatively small [12]. Therefore, accurate species identification is difficult based solely on morphological characteristics. Such morphological ambiguities highlight the need for complementary molecular or biochemical approaches to ensure reliable species delimitation.

With the development and refinement of molecular biological techniques, the classification and species concepts of *Colletotrichum* have become increasingly well-defined [13,14,15,16]. Jayawardena et al. [14] have provided molecular data for 248 species, divided into 14 species complexes and 13 singleton species. Subsequently, Liu et al. [16] have provided molecular data for 280 species, divided into 16 species complexes and 15 singleton species of *Colletotrichum*. *Colletotrichum karsti*, *C*. *siamense*, *C*. *fructicola*, *C*. *fioriniae*, and *C*. *gloeosporioides* are species with high isolation rates in China [16], primarily infecting woody plants such as walnut, *Camellia oleifera*, and *Cinnamomum camphora* [2].

Multiple distinct *Colletotrichum* species can infect the same host. Existing research indicates that walnut anthracnose can be caused by several groups, including the *Colletotrichum acutatum* species complex, the *C. boninense* species complex, and the *C. gloeosporioides* species complex [17,18,19,20,21]. De Silva et al. [22] report different *Colletotrichum* species causing anthracnose in chili peppers. These studies indicate that walnuts, *Camellia oleifera*, and chili peppers are more susceptible to infection by *Colletotrichum* fungi compared to other plants.

The aims of this study are to (1) systematically identify the species composition of anthracnose fungi on walnuts and determine whether these species differ in their geographical distribution and host adaptation and (2) expand the investigation to non-walnut hosts, exploring whether ecological isolation or coevolution exists between these hosts and the anthracnose fungi predominantly occurring on walnuts and reveal the pathogen diversity of the hosts.

## 2. Materials and Methods

### 2.1. Disease Survey and Sample Isolations

**Figure 1:**
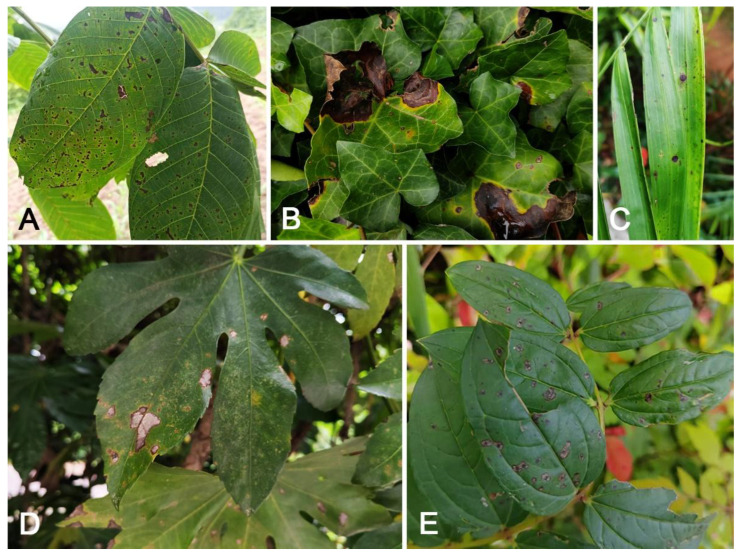
Diseased plants in Beijing, Fuzhou, and Guizhou: (**A**) symptoms of *Juglans regia* in Beijing; (**B**) leaf spots of *Hedera nepalensis* var. *sinensis* in Guizhou; (**C**) leaf spots of *Chamaedorea pinnatifrons* in Fuzhou; (**D**) pathogenic fungi on *Fatsia japonica* leaves in Guizhou; (**E**) leaf spots of *Coriaria napalensis* in Fuzhou.

From 2023 to 2024, a total of 110 symptomatic samples of branch cankers and leaf spots were collected from five provincial regions in China (Beijing, Fujian, Guangdong, Guizhou, and Shaanxi), among which 67 samples (Figure 1) exhibited typical anthracnose symptoms. The disease samples were placed in specimen bags, and detailed sample information was recorded. They were then brought back to the laboratory for isolation. The tissue isolation method was used to isolate the leaf spot samples [23]. The fresh fruiting bodies on the branch cankers were cut open with a sterilized knife to expose the spore masses. The spore masses were transferred to PDA plates with a sterilized needle to obtain pure cultures at 25 °C. Obtain the sexual and asexual morphs of fungi on PDA at 25 °C. Capture microscopic images of fungi using a DIC microscope. All samples have been deposited in the Museum of the Beijing Forestry University (BJFC) and the Institute of Microbiology, Chinese Academy of Sciences (IMCAS). The pure cultures obtained have been deposited in the China Forestry Culture Collection Center (CFCC, https://cfcc.caf.ac.cn/) and the Laboratory of Forest Pathology Resources (LFPR), Beijing Forestry University.

### 2.2. DNA Extraction and PCR Amplification

Genomic DNA was extracted from cultures growing on a PDA medium using a modified CTAB method [24]. To identify the strains, we amplified the following gene loci: the internal transcribed spacer (ITS) regions, glyceraldehyde 3-phosphate dehydrogenase (*gapdh*), partial sequences of the chitin synthase 1 (*chs-1*), actin (*act*), β-tubulin 2 (*tub2*), Histone3 (*his3*), and Calmodulin (*cal*) [2,16]. We used the respective primer pairs: ITS1/ITS4, GDF1/GDR1, CHS-79F/CHS-345R, ACT-512F/ACT-783R, T1/Bt2b, CYLH3F/ CYLH3R, and CL1C/CL2C [12,16,25,26,27,28,29,30]. The PCR conditions for different genes used in the genus *Colletotrichum* are listed in Table 1. The amplicons were sequenced by the SinoGenoMax Company Limited (Beijing, China). All sequence data obtained in this study have been submitted to GenBank (Supplementary Table S1).

### 2.3. Phylogenetic Analysis

*Colletotrichum* species sequences were downloaded from NCBI for phylogenetic analysis, referring to Zhang and Chen et al. [31], Zhang and Nizamani et al. [7], Sui et al. [2], and Li et al. [32]. Sequence assembly was performed using Seqman v. 7.1.0 software. Alignment was conducted using MAFFT v. 7 [33] (http://mafft.cbrc.jp/alignment/server/index.html, accessed on 7 October 2025). Sequence trimming and editing were carried out using MEGA v. 6 [34]. Phylogenetic analysis was performed using maximum likelihood (ML) and Bayesian inference (BI). PhyML v. 3.0 was used for ML analysis, with the GTR nucleotide substitution model and the bootstrap (BS) method with 1000 replicates [35]. Bayesian Inference (BI) analysis was performed using the Markov chain Monte Carlo (MCMC) algorithm [36]. Two MCMC chains were run starting from a random tree for 1,000,000 generations, with trees saved every 1000 generations and the first 25% of trees discarded as burn-in for posterior probability (BPP). The BPP of each analysis was used to evaluate the remaining trees. The phylogenetic tree was visualized in FigTree v.1.3.1 [37].

### 2.4. Correlation Analysis Between Pathogens and Hosts

Correlation analysis between pathogens and hosts was performed using Excel 2020 software. A structured database was constructed by integrating species identification results of isolated strains, host information, and geographic origins (provincial/municipal sampling sites). Occurrence frequency and relative abundance of different species were calculated using Excel pivot tables to clarify differences in the composition of dominant species across hosts. The relative abundances of different species in walnut were computed using the pie chart tool in Excel 2020 software to evaluate the dominant species in walnut. Combined with geographic and host information, evolutionary and ecological associations were further elucidated.

## 3. Results

### 3.1. Phylogenetic Analyses

Based on the single-gene phylogenetic analysis of the ITS region, it was confirmed that the 67 isolates belong to six species complexes of *C*. *acutatum*, *C*. *boninense*, *C*. *destructivum*, *C*. *gloeosporioides*, *C*. *orchidearum*, and *C*. *spaethianum*. Based on multi-gene phylogenetic analysis (ITS, *gapdh*, *chs-1*, *act*, *tub2*, *his3*, and *cal*), the 67 isolates belonged to four new species and 12 known species within the six species complexes, with the specific results as follows:

#### 3.1.1. *Colletotrichum acutatum* Species Complex Phylogenetic Analysis

**Figure 2:**
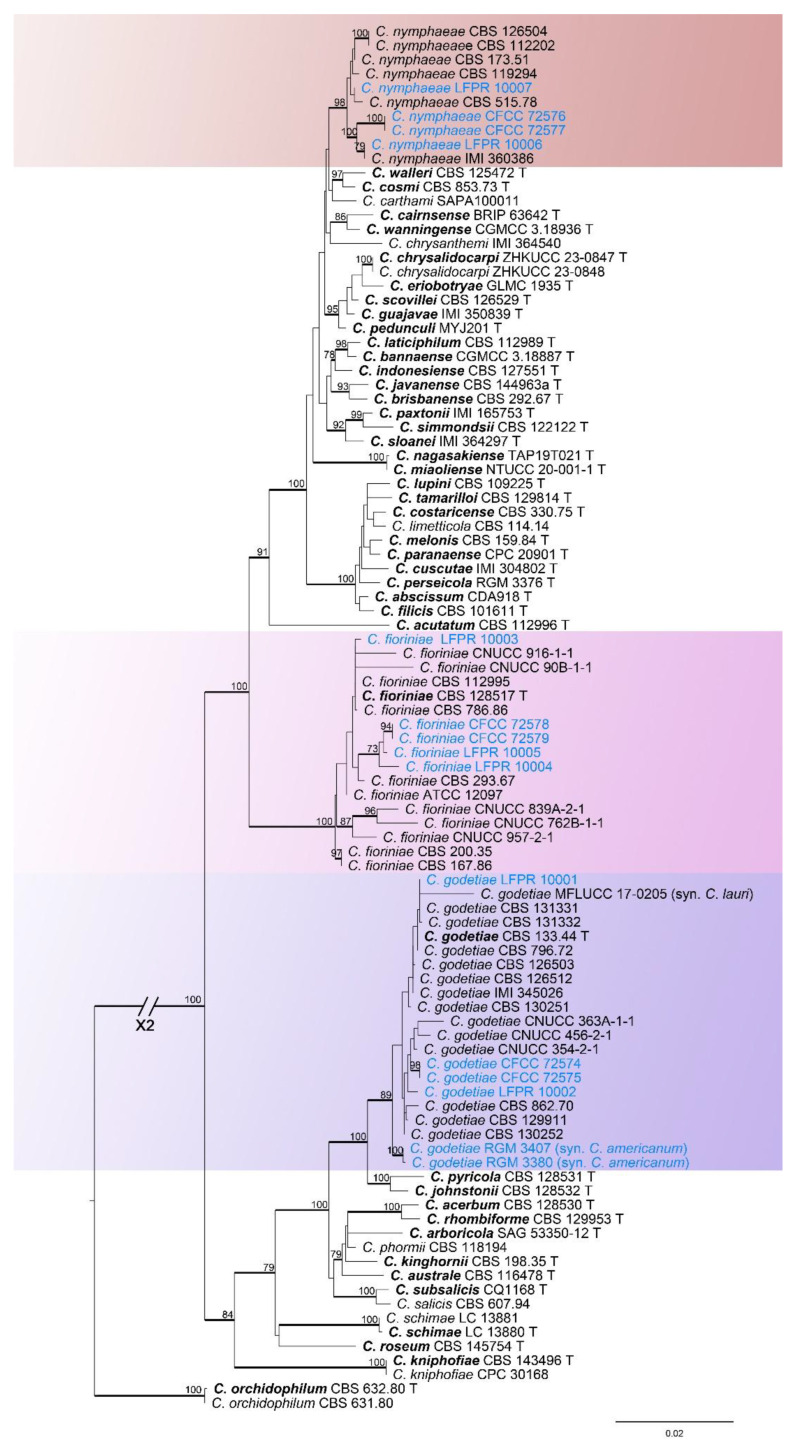
Phylogenetic tree of the *Colletotrichum acutatum* species complex resulting from maximum likelihood analysis. Numbers at the nodes indicate ML bootstrap values (ML-BS ≥ 70%) and Bayesian posterior probabilities (BPP ≥ 0.90) are emphasized by thickened branches. Ex-type strains are marked with “T” and presented in bold. Strains from this study are shown in blue.

The *Colletotrichum acutatum* species complex was analyzed using multiple gene loci (ITS, *gapdh*, *chs-1*, *act*, *tub2*, and *his3*) (Figure 2). The outgroup was *C. orchidophilum* (CBS 632.80 and CBS 631.80). In the ML analysis based on the combined gene dataset, the best likelihood value was −11,632.287603. The matrix had 846 distinct alignment patterns. The proportion of gaps and completely undetermined characters was 12.39%. The estimated base frequencies were as follows: A = 0.214646, C = 0.281884, G = 0.266176, T = 0.237294; substitution rates: AC = 0.901509, AG = 3.938106, AT = 1.080309, CG = 0.554337, CT = 4.209148, GT = 1.000000; the shape parameter of the gamma distribution α = 0.247371. The 13 strains in this study formed three distinct clades, with 13 strains clustering with *C*. *fioriniae*, *C*. *godetiae*, and *C*. *nymphaeae*, respectively (Figure 2). All three clades were supported with over 89% ML and 1.00 Bayesian posterior probability (BYPP). The 13 strains were identified as three known species (*C*. *fioriniae*, *C*. *godetiae*, and *C*. *nymphaeae*) within the *C*. *acutatum* species complex (Figure 2).

#### 3.1.2. *Colletotrichum boninense* Species Complex Phylogenetic Analysis

**Figure 3:**
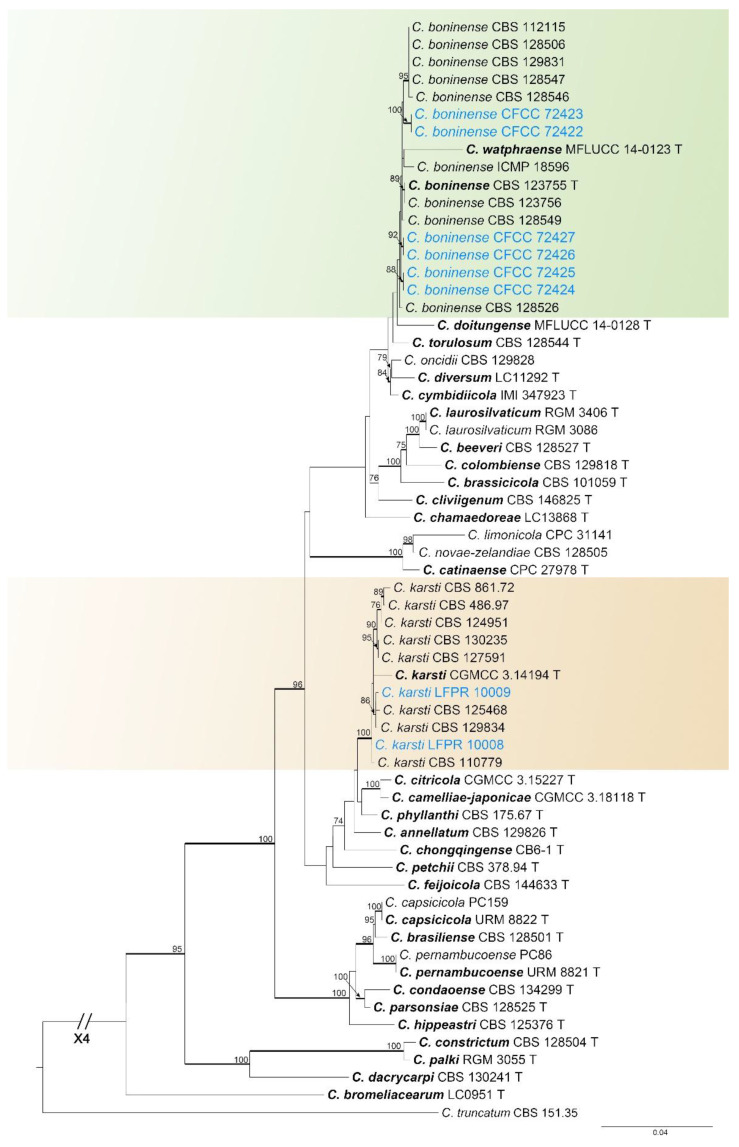
Phylogenetic tree of the *Colletotrichum boninense* species complex resulting from maximum likelihood analysis. *Colletotrichum truncatum* (CBS 151.35) was used as the outgroup. Maximum likelihood bootstrap values (ML-BS ≥ 70%) are shown above the branches, and Bayesian posterior probabilities (BPP ≥ 0.90) are indicated by thickened branches. Ex-type strains are marked with “T” and presented in bold. Strains from this study are marked in blue.

The *Colletotrichum boninense* species complex was analyzed using multiple gene loci (ITS, *gapdh*, *chs-1*, *act*, *tub2*, and *his3*) (Figure 3). *Colletotrichum truncatum* (CBS 151.35) was used as the outgroup. The best likelihood value of the ML tree was −11,896.599473. The matrix had 939 distinct alignment patterns. The proportion of gaps and completely undetermined characters was 14.06%. The estimated base frequencies were as follows: A = 0.230169, C = 0.259637, G = 0.298228, T = 0.211966; substitution rates: AC = 1.062959, AG = 3.732917, AT = 0.904273, CG = 0.735975, CT = 3.422578, GT = 1.000000; the shape parameter of the gamma distribution α = 0.302366. The *C*. *boninense* species complex involved eight strains in this study, which formed four separate clades (Figure 3). The ML values for the three clades were above 90% and the Bayesian posterior probabilities (BYPP) were above 0.9. The six strains were identified as *C*. *boninense*, and two strains were identified as *C*. *karsti* (Figure 3).

#### 3.1.3. *Colletotrichum destructivum* Species Complex Phylogenetic Analysis

**Figure 4:**
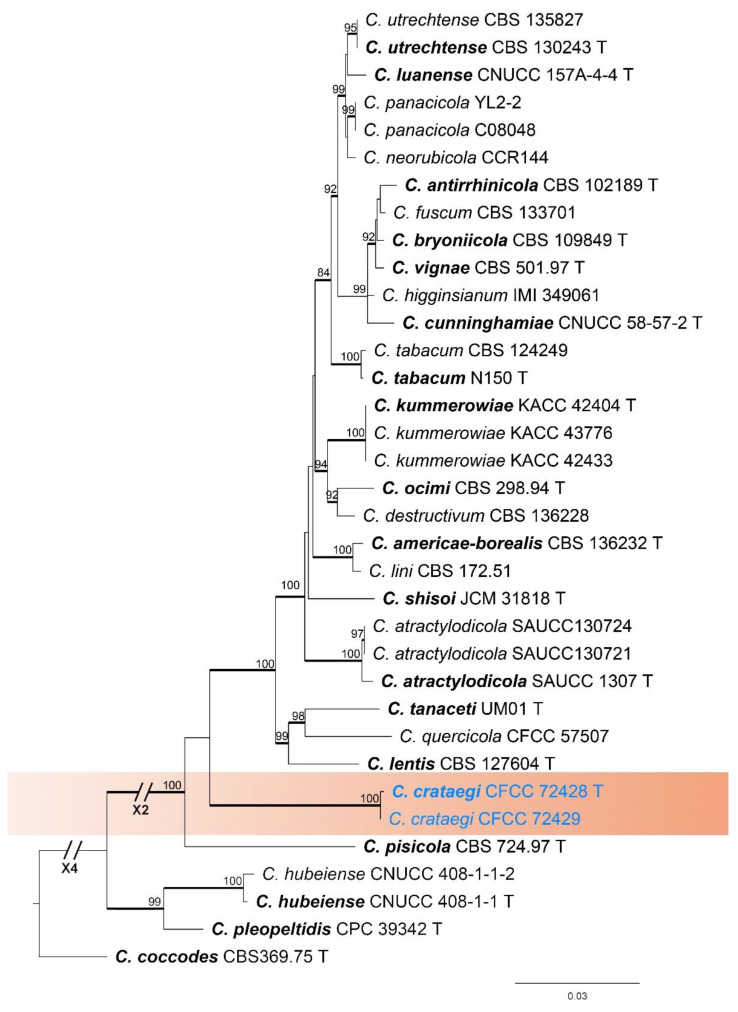
Phylogenetic tree of the *Colletotrichum destructivum* species complex based on maximum likelihood analysis. Maximum likelihood bootstrap support values (ML-BS ≥ 70%) are shown at the nodes, and Bayesian posterior probabilities (BPP ≥ 0.90) are indicated by thickened branches. Ex-type strains are marked with “T” and presented in bold. Strains from this study are shown in blue.

The *Colletotrichum destructivum* species complex was analyzed using multiple gene loci (ITS, *gapdh*, *chs-1*, *act*, *tub2*, and *his3*) (Figure 4). *Colletotrichum coccodes* (CBS 369.75) was selected as an outgroup. The best likelihood value for the *C*. *destructivum* species complex was −8634.934944. The matrix had 651 distinct alignment patterns. The proportion of gaps and completely undetermined characters was 15.49%. The estimated base frequencies were as follows: A = 0.216431, C = 0.268314, G = 0.287624, T = 0.227631; substitution rates: AC = 1.353115, AG = 5.041429, AT = 1.199580, CG = 0.588826, GT = 1.000000, CT = 4.193986; the shape parameter of the gamma distribution α = 0.253411. Two strains in this study formed a single, separate clade, which was identified as one new species (*C*. *crataegi*) within the *C*. *destructivum* species complex.

#### 3.1.4. *Colletotrichum gloeosporioides* Species Complex Phylogenetic Analysis

**Figure 5:**
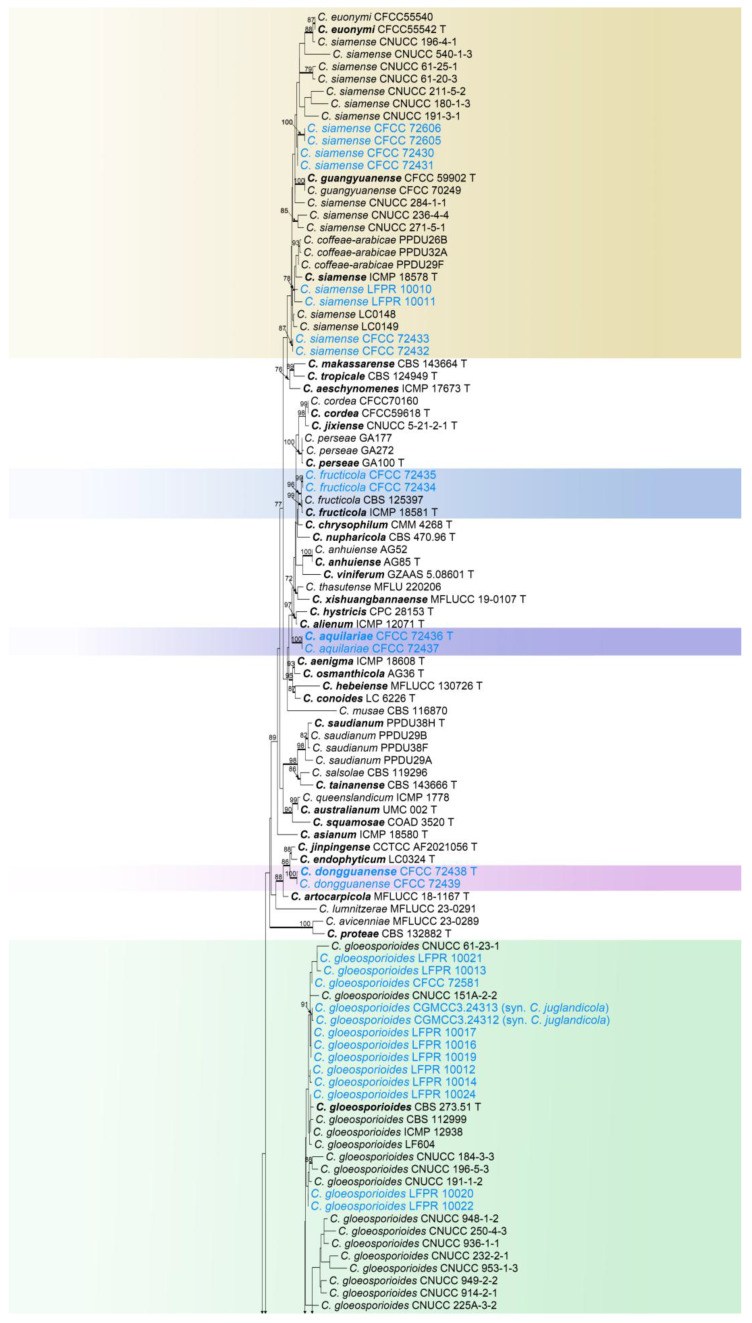

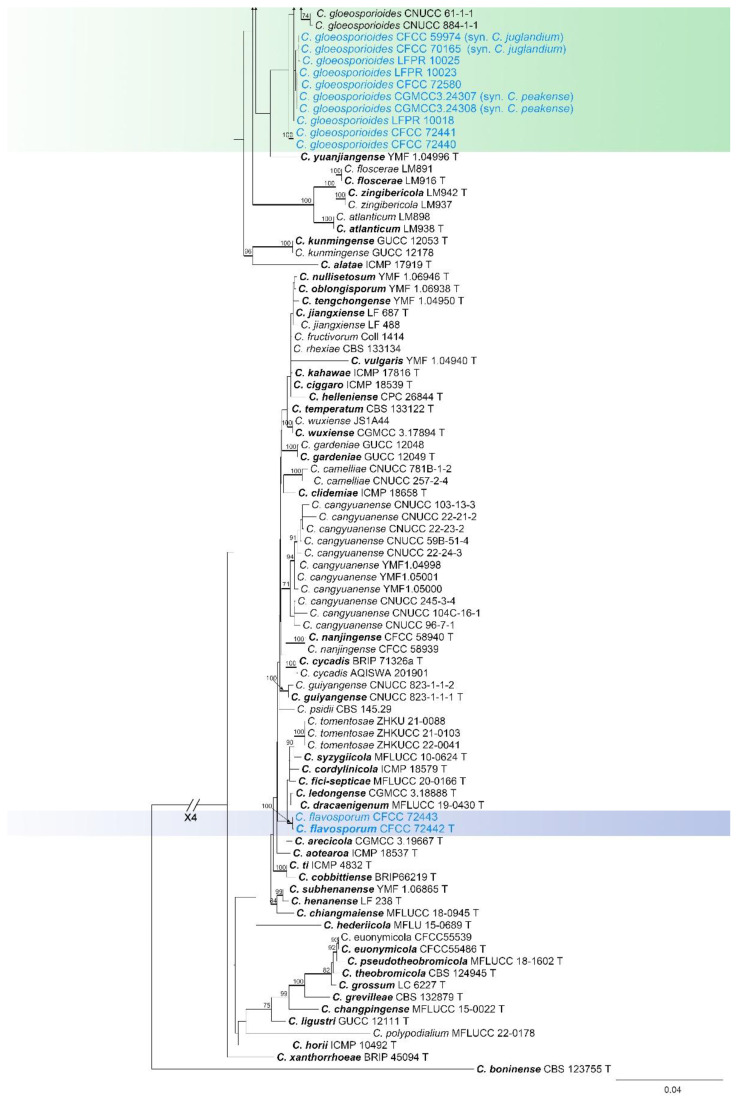
Phylogenetic tree of the *Colletotrichum gloeosporioides* species complex based on maximum likelihood analysis. Numbers at the nodes indicate maximum likelihood bootstrap values (ML-BS ≥ 70%) and Bayesian posterior probabilities (BPP ≥ 0.90) are emphasized by thickened branches. Ex-type strains are indicated with “T” and presented in bold. Strains from this study are marked in blue.

The *Colletotrichum gloeosporioides* species complex was analyzed using multiple gene loci (ITS, *gapdh*, *chs-1*, *act*, *tub2*, and *cal*) (Figure 5). The outgroup was *C*. *boninense* (CBS 123755). The best likelihood value was −21,030.171117. The matrix had 1574 distinct alignment patterns with a 30.93% proportion of gaps and completely undetermined characters. The estimated base frequencies were as follows: A = 0.225648, C = 0.295175, G = 0.251021, T = 0.228155; substitution rates: AC = 1.180654, AG = 3.528904, AT = 1.177066, CG = 0.872057, GT = 1.000000, CT = 3.895772; the shape parameter of the gamma distribution α = 0.404514. 28 strains in this study were identified as three known species (*C*. *fructicola*, *C*. *siamense*, and *C*. *gloeosporioides*) (Figure 5). Six strains were identified as three new species (*C*. *aquilariae*, *C*. *dongguanense*, and *C*. *flavosporum*) within the *C*. *gloeosporioides* species complex (Figure 5). The ML values for these eight species were all above 95% and the Bayesian posterior probabilities (BYPP) were all 1.00.

#### 3.1.5. *Colletotrichum orchidearum* Species Complex Phylogenetic Analysis

**Figure 6:**
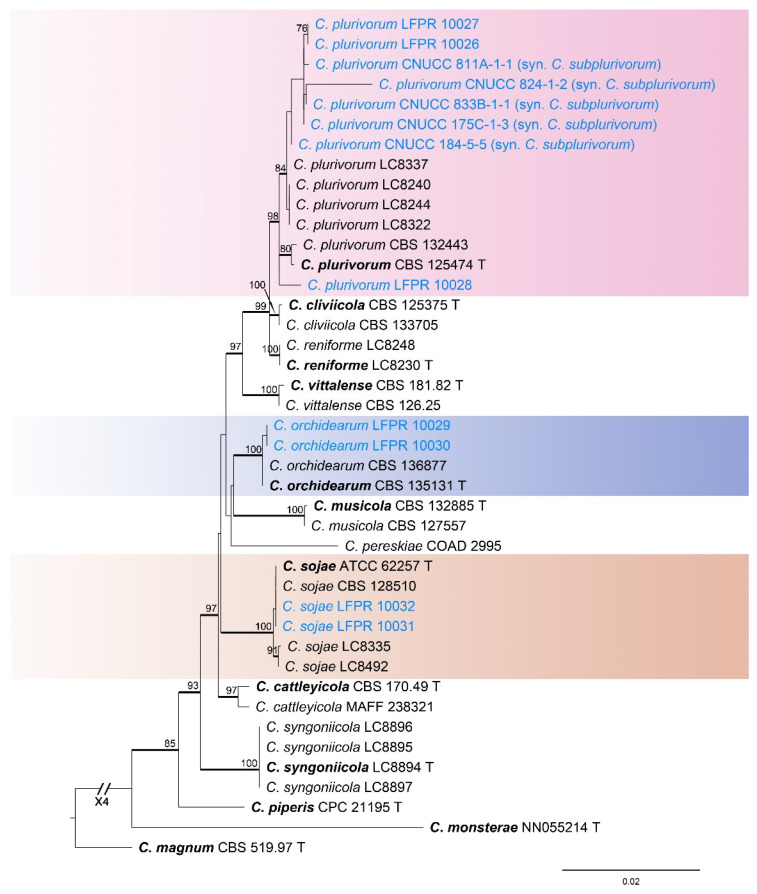
Phylogenetic tree of the *Colletotrichum orchidearum* species complex based on maximum likelihood analysis. Numbers at the nodes indicate maximum likelihood bootstrap values (ML-BS ≥ 70%) and Bayesian posterior probabilities (BPP ≥ 0.90) are emphasized by thickened branches. Ex-type strains are indicated with “T” and presented in bold. Strains from this study are marked in blue.

The *Colletotrichum orchidearum* species complex was analyzed using multiple gene loci (ITS, *gapdh*, *chs-1*, *act*, *tub2*, and *his3*) (Figure 6). The outgroup was *C*. *magnum* (CBS 519.97). The best likelihood value was −5875.108038. The matrix had 434 distinct alignment patterns with an 11.21% proportion of gaps and completely undetermined characters. The estimated base frequencies were as follows: A = 0.212098, C = 0.315800, G = 0.257503, T = 0.214599; substitution rates: AC = 1.108763, AG = 3.317336, AT = 0.795605, CG = 0.686179, GT = 1.000000, CT = 4.883049; the shape parameter of the gamma distribution α = 0.212737. Seven strains in this study were identified as three known species (*C*. *orchidearum*, *C*. *plurivorum*, and *C*. *sojae*) (Figure 6). The ML values for these known species were all above 98% and the Bayesian posterior probabilities (BYPP) were all 1.00.

#### 3.1.6. *Colletotrichum spaethianum* Species Complex Phylogenetic Analysis

**Figure 7:**
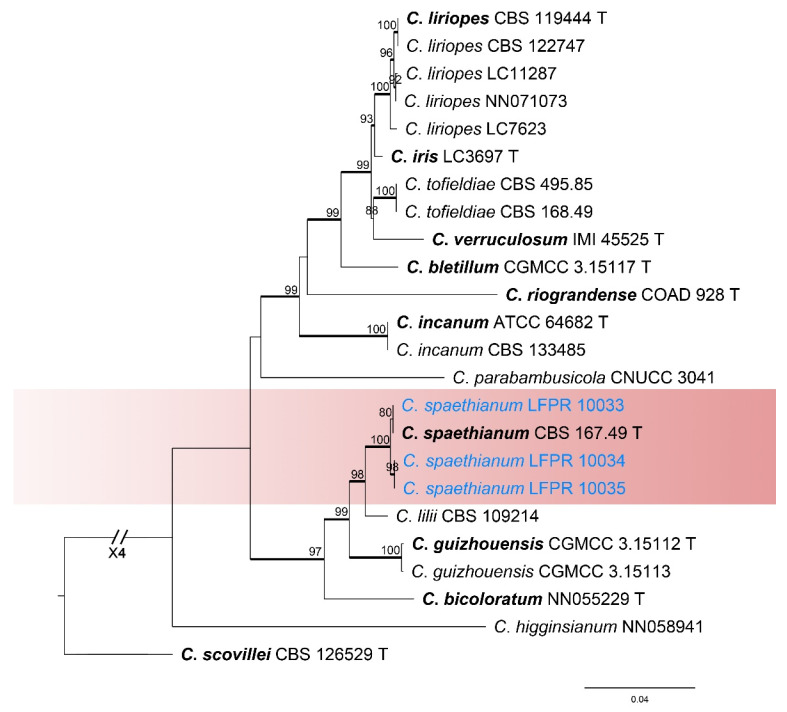
Phylogenetic tree of the *Colletotrichum spaethianum* species complex based on maximum likelihood analysis. Numbers at the nodes indicate maximum likelihood bootstrap values (ML-BS ≥ 70%) and Bayesian posterior probabilities (BPP ≥ 0.90) are emphasized by thickened branches. Ex-type strains are indicated with “T” and presented in bold. Strains from this study are marked in blue.

The *Colletotrichum spaethianum* species complex was analyzed using multiple gene loci (ITS, *gapdh*, *chs-1*, *act*, *tub2*, and *his3*) (Figure 7). The outgroup was *C*. *scovillei* (CBS 126529). The best likelihood value was −8359.303202. The matrix had 675 distinct alignment patterns with a 17.29% proportion of gaps and completely undetermined characters. The estimated base frequencies were as follows: A = 0.219187, C = 0.303015, G = 0.250183, T = 0.227615; substitution rates: AC = 0.958726, AG = 3.796058, AT = 1.123702, CG = 0.680117, GT = 1.000000, CT = 5.575032; the shape parameter of the gamma distribution α = 0.284355. Three strains in this study were identified as *C*. *spaethianum* (Figure 7). The ML values for these known species were all above 100% and the Bayesian posterior probabilities (BYPP) were all 1.00.

### 3.2. Taxonomy

The 67 strains studied were classified into 16 species, including 4 novel species and 12 known species. Detailed morphological descriptions are provided below for all the species studied in culture. Exceptions were made for the 9 known species *C*. *fioriniae*, *C*. *fructicola*, *C*. *godetiae*, *C*. *karsti*, *C*. *nymphaeae*, *C*. *orchidearum*, *C*. *plurivorum*, *C*. *sojae*, and *C*. *spaethianum* as they have already been described in great detail in the studies by Sui et al. [2], Zhang et al. [7], de Silva et al. [22], Damm et al. [38] and Damm et al. [10], and Damm et al. [39].

**Figure 8:**
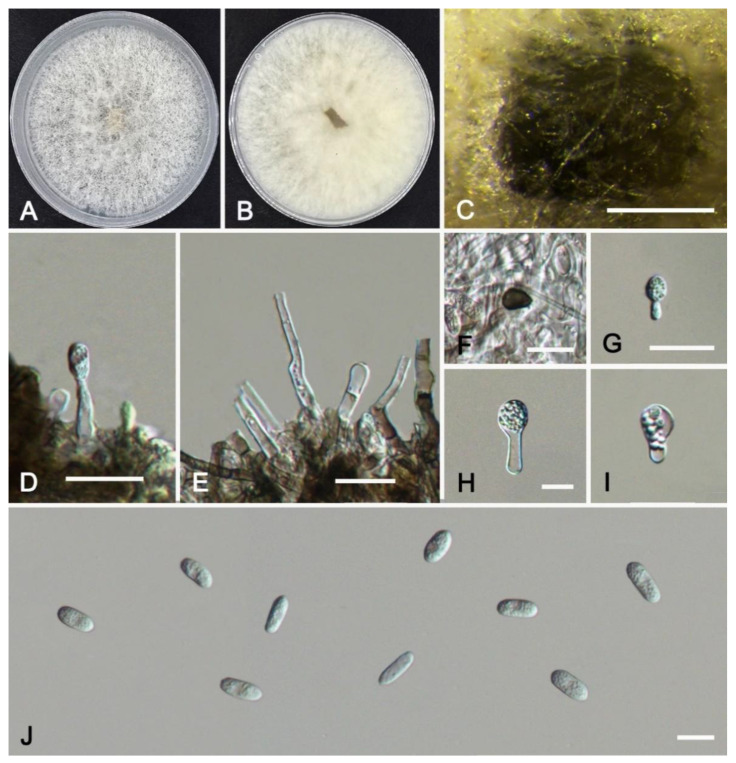
*Colletotrichum aquilariae* from *Aquilaria sinensis* (HMAS 353996): (**A**,**B**) front and reverse colony on PDA (7 d); (**C**) conidial masses formed on PDA; (**D**,**E**) conidiogenous cells; (**F**–**I**) appressoria; (**J**) conidia. Scale bars: (**C**) = 200 μm; (**D**–**J**) = 10 μm.

***Colletotrichum aquilariae*** W.S. Zhang & X.L. Fan, sp. nov. (Figure 8).

*MycoBank*: MB859290

*Etymology*: Named after the host genus of the collected sample, *Aquilaria*.

*Holotype*: HMAS 353996

*Description*: Sexual morph not observed. Asexual morph: Sporulating on PDA. Conidiomata scattered or gregarious, acervular, semi-immersed to immersed, hyaline or dark brown, unbranched, septate. Conidiophores usually reduced to conidiogenous cells, unbranched. Conidiogenous cells hyaline or dark brown, straight or curved, cylindrical or ampulliform, smooth-walled, 8.5–19.5(–47.0) × 1.5–3.1 μm (av. = 12.5 × 2.2 μm, n = 50). Conidia hyaline, cylindrical, aseptate, smooth-walled, no oil droplets, 10.5–12.5 × 3.5–4.8 μm (av. = 11.2 × 4.1 μm, n = 50), L/W ratio = 2.7. Appressoria elliptical to sub-elliptical, hyaline to dark brown, single, 4.5–10.8 × 2.7–8.5 μm (av. = 5.9 × 4.2 μm, n = 20). Setae not observed.

*Culture characteristics*: Colonies on PDA white, spreading, with the center of colonies having abundant white flocculent aerial mycelium and even margin, reaching a diameter of 60 mm after 8 d at 25 °C.

*Typus*: CHINA, Guangdong Province, Dongguan City, Xingtang Xinghua Road, collected from diseased leaves of *Aquilaria sinensis*, 113°44′40″ E, 22°57′33″ N, 7 December 2023, Xinlei Fan (holotype HMAS 353996; ex-holotype culture CFCC 72436); *ibid*. (living culture CFCC 72437).

*Notes*: *Colletotrichum aquilariae* is revealed in the multi-gene phylogram as a distinct clade with full support (BI/ML = 1.00/100) (Figure 5). *Colletotrichum aquilariae* forms a group close to *C*. *alienum* and *C*. *hystricis*, but it differs from *C*. *alienum* in the nucleotide sequence by 6 bp in the ITS, 4 bp in *gapdh*, 6 bp in *act*, 4 bp in *tub2*, and 5 bp in *cal*. It can also differ from *C*. *hystricis* in the nucleotide sequence by 7 bp in the ITS, 6 bp in *gapdh*, and 10 bp in *act*. Morphologically, the conidia of *C*. *aquilariae* are smaller than those of *C*. *alienum* (10.5–12.5 × 3.5–4.8 vs. 15.5–17.5 × 5–5.5 µm) and *C*. *hystricis* (10.5–12.5 × 3.3–4.8 vs. 13–15 × 4–5.5 µm).

**Figure 9:**
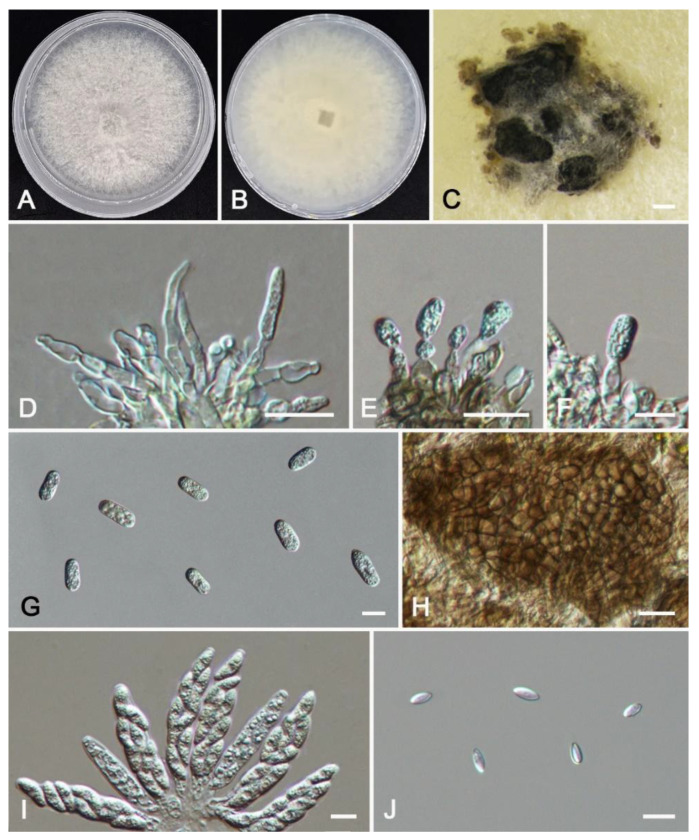
*Colletotrichum boninense* from *Hedera nepalensis* (living culture CFCC 72426): (**A**,**B**) front and reverse colony on PDA (7 d); (**C**) conidial masses formed on PDA; (**D**–**F**) conidiogenous cells; (**G**) conidia; (**H**) ascomata wall; (**I**) Asci; (**J**) conidia. Scale bars: (**C**) = 200 μm; (**D**–**J**) = 10 μm.

***Colletotrichum boninense*** Moriwaki, Toy. Sato & Tsukib., Mycoscience 44 (1): 48. 2003. (Figure 9).

*Description*: See Damm et al. [10].

*Material examined*: CHINA, Guizhou Province, Guiyang City, Aha Lake National Wetland Park, collected from diseased leaves of *Hedera nepalensis*, 106°36′59″ E, 26°33′55″ N, 27 June 2024, Weishan Zhang (HMAS 353991, living culture CFCC 72426); *ibid*. (living culture CFCC 72427); CHINA, Guizhou Province, Bijie City, Dafang County, collected from diseased leaves of *Coriaria napalensis*, 19 June 2024, 105°26′33″ E, 27°12′41″ N, Xinlei Fan (HMAS 353992, living culture CFCC 72424); *ibid*. (living culture CFCC 72425); CHINA, Guizhou Province, Bijie City, Dafang County, collected from diseased leaves of *Fatsia japonica*, 19 June 2024, 105°26′33″ E, 27°12′41″ N, Xinlei Fan (HMAS 353993, living culture CFCC 72422); *ibid*. (living culture CFCC 72423).

*Culture characteristics*: After culturing in the dark at 25 °C for 10 days, the colonies on PDA can reach 90 mm, white in color, with abundant flocculent aerial mycelium on the surface, spreading in appearance, and even margin. Conidial masses are produced after 15 days.

*Notes*: In the phylogenetic tree, the six isolates from this study clustered with *C*. *boninense* (Figure 3). Therefore, the six isolates were identified as *C*. *boninense* (Figure 2), representing one new host from China.

**Figure 10:**
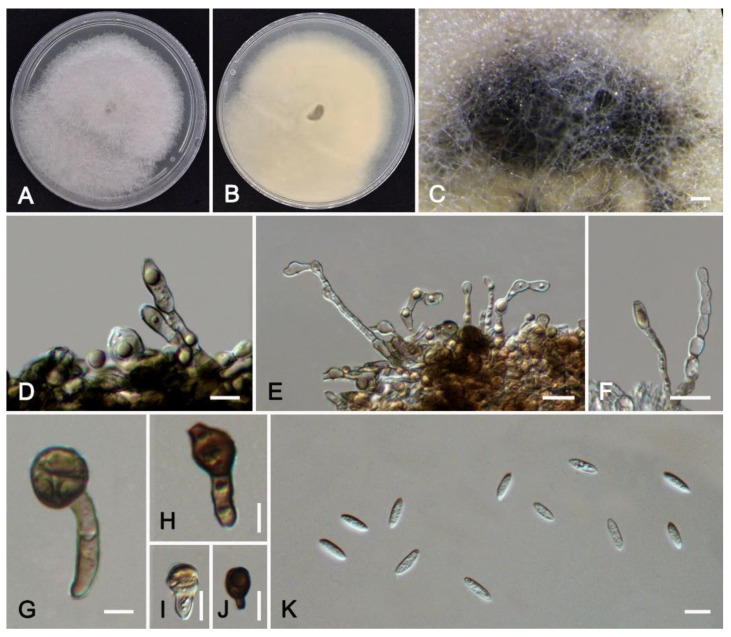
*Colletotrichum crataegi* from *Crataegus pinnatifida* (HMAS 353994): (**A**,**B**) front and reverse colony on PDA (7 d); (**C**) conidial masses formed on PDA; (**D**–**F**) conidiogenous cells; (**G**–**J**) appressoria; (**K**) conidia. Scale bars: (**C**) = 200 μm; (**D**–**K**) = 10 μm.

***Colletotrichum crataegi*** W.S. Zhang & X.L. Fan, sp. nov. (Figure 10).

*MycoBank*: MB859293

*Etymology*: Named after the host genus of the type specimen, *Crataegus*.

*Holotype*: HMAS 353994

*Description*: Sexual morph not observed. Asexual morph: Sporulating on PDA. Conidiomata hyaline to light brown, oil droplets, acervulus, septate, unbranched, irregular. Conidiophores usually reduced to conidiogenous cells, unbranched. Conidiogenous cells (25.0–)29.0–46.5 × 2.5–5.7 μm (av. = 36.1 × 3.9 μm, n = 30), irregular, hyaline or light brown, smooth-walled, oil droplets. Conidia 9.5–13.5 × 3.0–3.8 μm (av. = 11.5 × 3.4 μm, n = 50), L/W ratio = 3.3, fusiform, hyaline, smooth-walled, contents granular. Appressoria circular to elliptical, light brown to dark brown, smooth-walled, 7.6–12.1 × 6.2–9.5(–11.2) μm (av. = 10.1 × 8.3 μm, n = 30). Setae not observed.

*Culture characteristics*: Colonies on PDA white, with even margin and abundant flocculent aerial mycelium, reaching 60 mm in diameter after 8 d at 25 °C. Black conidial masses are formed on the medium after 15 days.

*Typus*: CHINA, Beijing City, Mentougou District, Xiaolongmen Forest Farm, collected from diseased leaves of *Crataegus pinnatifida*, 22 August 2024, 115°25′00″ E, 39°48′34″ N, Xinlei Fan (holotype HMAS 353994; ex-holotype culture CFCC 72428); *ibid*. (living ex-paratype culture CFCC 72429).

*Notes*: *Colletotrichum crataegi* was associated with anthracnose of *Crataegus pinnatifida*. It clusters in a sister phylogenetic clade with *C*. *lentis* (BI/ML = 1.00/100) (Figure 4). However, the conidiogenous cells of *C*. *crataegi* are longer than those of *C*. *lentis* (29.0–46.4 vs. 9–28 µm). The conidia of *C*. *crataegi* are shorter than those of *C*. *lentis* (9.5–13.5 vs. 16–20 µm). The appressoria of *C*. *crataegi* are larger (7.6–12.1 × 6.2–9.5 vs. 5.5–7.5 × 4.5–6 µm). In addition, it differs from *C*. *lentis* in the nucleotide sequence by 9 bp in the ITS, 32 bp in *gapdh*, 14 bp in *chs-1*, 21 bp in *act*, 46 bp in *tub2*, and 34 bp in *his3*.

**Figure 11:**
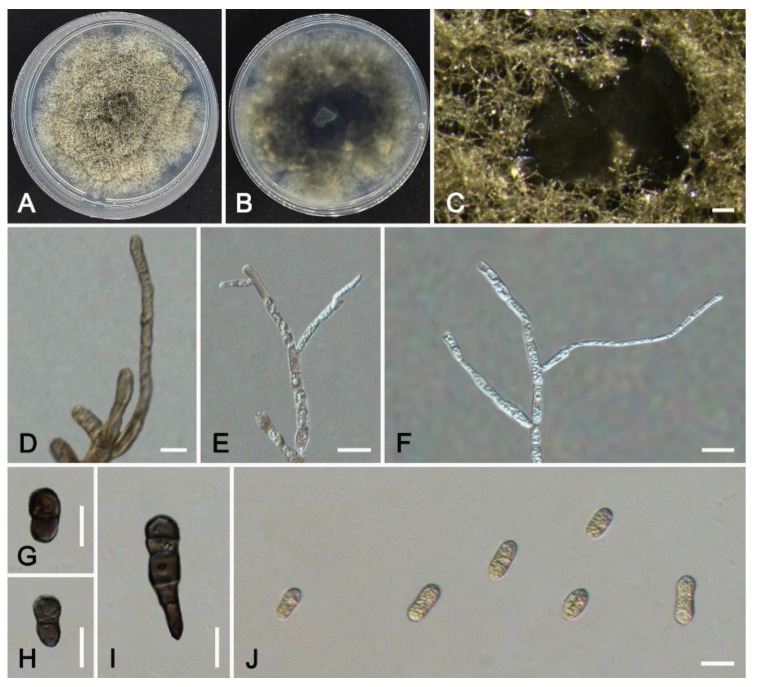
*Colletotrichum dongguanense* from *Bauhinia purpurea* (HMAS 353998): (**A**,**B**) front and reverse colony on PDA (7 d); (**C**) conidial masses formed on PDA; (**D**–**F**) conidiogenous cells; (**G**–**I**) appressoria; (**J**) conidia. Scale bars: (**C**) = 200 μm; (**D**–**J**) = 10 μm.

***Colletotrichum dongguanense*** W.S. Zhang & X.L. Fan, sp. nov. (Figure 11).

*MycoBank*: MB859294

*Etymology*: Named after the collection location of the type specimen, Dongguan.

*Holotype*: HMAS 353998

*Description*: Sexual morph not observed. Asexual morph: Sporulating on PDA. Conidiomata and setae not observed. Conidiophores not developed. Conidiogenous cells formed from mycelium directly, branched. Conidia hyaline or pale brown, cylindrical or sub-cylindrical, aseptate, smooth-walled, no oil droplets, contents granular, 11.5–13.9(–16.2) × 4.5–6.5 μm (av. = 13.0 × 5.5 μm, n = 50), L/W ratio = 2.4. Appressoria single, both ends rounded or one end slightly pointed, medium to dark brown, 12.9–15.5(–23.9) × 5.8–7.7 μm (av. = 14.5 × 6.7 μm, n = 30).

*Culture characteristics*: Colonies on PDA 60 mm diam in 10 d, irregular margins, brown, with dense aerial mycelium, reverse black in the center, brown towards the margin. Black conidial masses are formed on the medium after 30 days.

*Typus*: CHINA, Guangdong Province, Dongguan City, Xingtang Xinghua Road, collected from diseased leaves of Bauhinia purpurea, 113°44′51″ E, 22°58′12″ N, 22 November 2023, Xinlei Fan (holotype HMAS 353998; ex-holotype culture CFCC 72438); *ibid*. (living culture CFCC 72439).

*Notes*: *Colletotrichum dongguanense* was associated with anthracnose of *Bauhinia purpurea*. Phylogenetically, *C*. *dongguanense* is sister to *C*. *endophyticum* and *C*. *jinpingense* (Figure 5). *Colletotrichum dongguanense* differs from *C*. *endophyticum* in the nucleotide sequence by 7 bp in *gapdh*, 2 bp in *chs-1*, 2 bp in *act*, and 3 bp in *tub2*. It also differs from *C*. *jinpingense* in nucleotide sequence by 4 bp in *gapdh*, 4 bp in *chs-1*, 3 bp in *act*, 4 bp in *tub2*, and 4 bp in *cal*. The mycelial color of *C*. *endophyticum* and *C*. *jinpingense* is gray on PDA medium, but *C*. *dongguanense* is brown. The conidial length of *C*. *dongguanense* is shorter than that of *C*. *jinpingense* (11.5–13.9 vs. 13.9–18.5 μm). The appressoria formed by *C*. *dongguanense* are regularly shaped, but those formed by *C*. *endophyticum* and *C*. *jinpingense* are irregularly shaped. Moreover, the appressoria of *C*. *dongguanense* are longer than those of *C*. *endophyticum* (12.9–15.5 vs. 8–12 μm).

***Colletotrichum fioriniae*** (Marcelino & Gouli) Pennycook., Mycotaxon 132 (1): 150. 2017.

*Description*: See Zhang et al. [7].

*Material examined*: CHINA, Shaanxi Province, Ankang City, Hanbin District, Zigou Town, Erlang Village, collected from diseased leaves of *Juglans regia*, 108°57′32″ E, 32°55′24″ N, 14 May 2024, Lu Lin & Xinlei Fan (living culture CFCC 72578 and CFCC 72579); CHINA, Guizhou Province, Bijie City, Dafang County, Jinhai Lake Wetland Park, collected from diseased leaves of *Ulmus parvifolia*, 105°26′30″ E, 27°12′45″ N, 19 June 2024 Xinlei Fan (living culture LFPR 10003 and LFPR 10005); CHINA, Fujian Province, Fuzhou City, Jinan District, Fengchi Baiyun Cave Scenic Area, collected from diseased leaves of *Laurus nobilis*, 119°17′27″ E, 26°6′9″ N, 19 August 2024 Xinlei Fan (living culture LFPR 10004).

*Notes*: In the phylogenetic tree, the five isolates from this study clustered with *C*. *fioriniae* (BI/ML = 1.00/100) (Figure 2). Therefore, the five isolates were identified as *C*. *fioriniae* (Figure 2), representing three new hosts from China.

**Figure 12:**
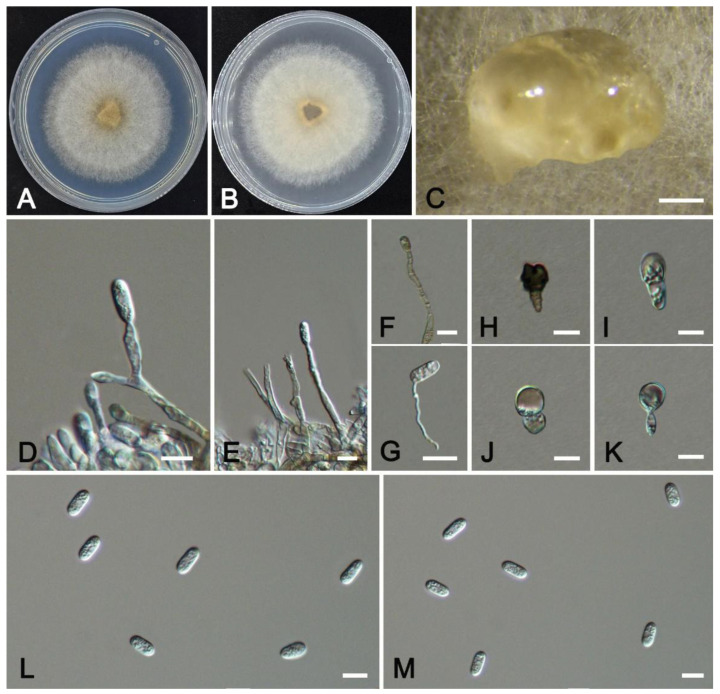
*Colletotrichum flavosporum* from *Bougainvillea glabra* (HMAS 353997): (**A**,**B**) front and reverse colony on PDA (7 d); (**C**) conidial masses formed on PDA; (**D**–**G**) conidiogenous cells; (**H**–**K**) appressoria; (**L**,**M**) conidia. Scale bars: (**C**) = 200 μm; (**D**–**M**) = 10 μm.

***Colletotrichum flavosporum* W.S. Zhang & X.L. Fan, sp. nov**. (Figure 12).

*MycoBank*: MB859297

*Etymology*: Named after the rare characteristic of this new species, *flavosporum*.

*Holotype*: HMAS 353997

*Description*: Sexual morph not observed. Asexual morph: Sporulating on PDA. Conidiomata scattered or gregarious, semi-immersed to immersed, hyaline. Conidiophores usually reduced to conidiogenous cells, branched. Conidiogenous cells hyaline or pale brown, straight or curved, cylindrical or ampulliform, smooth-walled, 19.2–22.8(–33.5) × 2.1–4.3 μm (av. = 21.1 × 3.4 μm, n = 50). Conidia cylindrical or sub-cylindrical, hyaline, contents granular, aseptate, smooth-walled, no oil droplets, 10.5–11.9 × 4.5–5.4 μm (av. = 11.2 × 5.0 μm, n = 50), L/W ratio = 2.2. Appressoria single, circular to irregular, hyaline or dark brown, (13.8–)17.1–20.5 × 7.5–10.5 μm (av. = 18.7 × 9.1 μm, n = 30). Setae not observed.

*Culture characteristics*: Colonies on PDA reach a diameter of 50 mm after 7 d at 25 °C, with regular margins, white in color, flat, and featuring dense aerial mycelium that exhibits a spreading growth habit.

*Typus*: CHINA, Guangdong Province, Dongguan City, Xingtang Xinghua Road, collected from diseased leaves of *Bougainvillea glabra*, 113°44′53″ E, 22°57′38″ N, 19 December 2024, Xinlei Fan (holotype HMAS 353997; ex-holotype culture CFCC 72442); *ibid*. (living culture CFCC 72443).

*Notes*: *Colletotrichum flavosporum* was associated with anthracnose of *Bougainvillea glabra.* Phylogenetically, *C*. *flavosporum* is closely related to *C*. *dracaenigenum* (Figure 5), but it differs from *C*. *dracaenigenum* in nucleotide sequence by 6 bp in ITS and 8 bp in *gapdh.* Morphologically, *C*. *flavosporum* produces branched conidiophores. The conidiogenous cells of *C*. *flavosporum* are longer than those of *C*. *dracaenigenum* (19.2–22.8 vs. 13–15 μm). The appressoria of *C*. *flavosporum* are larger than those of *C*. *dracaenigenum* (17.1–20.5 × 7.5–10.5 vs. 5–12 × 4–7 μm).

***Colletotrichum fructicola*** Prihast. et al., Fungal Diversity 39: 158. 2009.

*Description*: See de Silva et al. [22].

*Material examined*: CHINA, Beijing City, Shunyi District, County Road 408, collected from diseased leaves of *Juglans regia*, 116°39′17″ E, 40°7′49″ N, 10 October 2024, Xinlei Fan (living culture CFCC 72434 and CFCC 72435).

*Notes*: In this study, the two strains clustered with *C*. *fructicola* in a subclade, with 94% ML value and 1.00 BI value (Figure 5). The ITS, *chs-1*, *act*, *tub2*, and *cal* genes of the two strains were identical to those of *C*. *fructicola*, with only a 2 bp difference in *gapdh* (309/311, 99.4%), and they were morphologically similar. Therefore, we identified them as *C*. *fructicola*.

**Figure 13:**
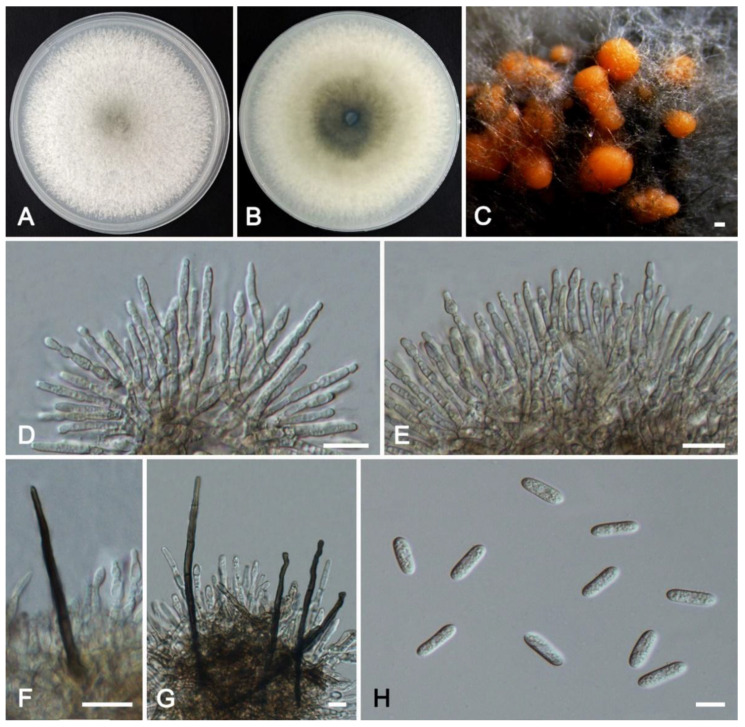
*Colletotrichum gloeosporioides* from *Juglans regia* (CFCC 72580): (**A**,**B**) front and reverse colony on PDA (7 d); (**C**) conidial masses formed on PDA; (**D**,**E**) conidiogenous cells; (**F**,**G**) Setae; (**H**) conidia. Scale bars: (**C**) = 200 μm; (**D**–**H**) = 10 μm.

***Colletotrichum gloeosporioides*** (Penz.) Penz. & Sacc., Atti del Reale Istituto Veneto di Scienze, Lettere ed Arti, 6, 2 (5): 670. 1884. (Figure 13).

New proposed synonyms.

*Colletotrichum peakense* L. Zhang et al. Mycokeys 99: 141 (2023).

*Colletotrichum juglandicola* L. Zhang et al. Mycokeys 99: 139 (2023).

*Colletotrichum juglandium* Y.X. Li et al. Mycokeys 108: 158 (2024).

*Description*: Sexual morph not observed. Asexual morph: Sporulating on PDA. Conidiomata scattered or gregarious, semi-immersed, hyaline. Conidiophores usually reduced to conidiogenous cells, unbranched, aseptate. Conidiogenous cells hyaline, straight, cylindrical, smooth-walled, 23.5–33.5 × 1.7–2.8 μm (av. = 27.3 × 2.2 μm, n = 50). Conidia cylindrical, hyaline, contents granular, smooth-walled, no oil droplets, 14.9–17.5 × 4.0–5.2 μm (av. = 16.1 × 4.6 μm, n = 50), L/W ratio = 3.4. Setae medium to dark brown, single, circular to irregular, 3–4-septate, 36–43 μm. Appressoria not observed.

*Material examined*: CHINA, Beijing City, Shunyi District, County Road 408, collected from diseased leaves of *Juglans regia*, 116°39′15″ E, 40°7′52″ N, 10 July 2024, Xinlei Fan (living culture CFCC 72580). *ibid*. 11 October 2024, Xinlei Fan (living culture CFCC 72581). *ibid*. 116°39′15″ E, 40°7′49″ N, 10 July 2024, Xinlei Fan (living culture LFPR 10014); CHINA, Beijing City, Mentougou District, Xiaolongmen Forest Farm, collected from diseased leaves of *Juglans regia*, 115°25′13″ E, 39°48′45″ N, 22 August 2024, Xinlei Fan (living culture LFPR 10025). CHINA, Beijing City, Haidian District, Cuihu National Urban Wetland Park, collected from diseased leaves of *Prunus cerasifera ‘Atropurpurea’*, 116°39′17″ E, 40°7′49″ N, 18 July 2024, Weishan Zhang (living culture CFCC 72440 and CFCC 72441). *ibid*. collected from diseased leaves of *Robinia pseudoacacia*, 116°10′45″ E, 40°6′10″ N, 18 July 2024, Weishan Zhang (living culture LFPR 10019); *ibid*. collected from diseased leaves of *Fraxinus chinensis*, 116°10′58″ E, 40°6′10″ N, 18 July 2024, Weishan Zhang (living culture LFPR 10020); *ibid*. collected from diseased leaves of *Prunus persica ‘Duplex’*, 116°11′21″ E, 40°6′18″ N, 13 October 2024, Weishan Zhang (living culture LFPR 10022); *ibid*. collected from diseased leaves of *Malus spectabilis*, 116°11′15″ E, 40°6′19″ N, 13 October 2024, Weishan Zhang (living culture LFPR 10023); *ibid*. collected from diseased leaves of *Kerria japonica*, 116°11′17″ E, 40°6′21″ N, 13 October 2024, Weishan Zhang (living culture LFPR 10024); *ibid*. collected from diseased leaves of *Amorpha fruticosa*, 116°11′6″ E, 40°6′9″ N, 18 July 2024, Weishan Zhang (living culture LFPR 10012); *ibid*. 116°11′9″ E, 40°6′9″ N, 18 July 2024, Weishan Zhang (living culture LFPR 10013); *ibid*. 116°11′15″ E, 40°6′20″ N, 18 July 2024, Weishan Zhang (living culture LFPR 10021). CHINA, Guizhou Province, Guiyang City, Qianling Mountain Park, collected from diseased leaves of *Quercus aliena*, 106°41′44″ E, 26°35′27″ N, 19 June 2024, Xinlei Fan (living culture LFPR 10015); *ibid*. Aha Lake National Wetland Park, collected from diseased leaves of *Parthenocissus quinquefolia*, 106°36′59″ E, 26°33′57″ N, 27 June 2024, Xinlei Fan (living culture LFPR 10016); CHINA, Shanxi Province, Ankang City, Hanbin District, Zigou Town, Erlang Village, collected from diseased leaves of *Parthenocissus tricuspidata*, 108°57′52″ E, 32°55′40″ N, 13 June 2024, Xinlei Fan (living culture LFPR 10017); *ibid*. collected from diseased leaves of *Microlepia marginata*, 108°57′50″ E, 32°55′41″ N, 13 June 2024 Xinlei Fan (living culture LFPR 10018).

*Notes*: Phylogenetically, the four strains are closely related to *C. gloeosporioides*, *C. juglandium*, *C. juglandicola*, and *C. peakense*. (BI/ML = 1.00/100) (Figure 5). Strains CFCC 72580 and CFCC 72581 had no base differences in the ITS, *gapdh*, *chs-1*, *act*, *tub2*, and *cal* when compared with *C. gloeosporioides* and *C. peakense*. Strains CFCC 72440 and CFCC 72441 had base differences in *gapdh* (10 bp) compared with *C. gloeosporioides*. The isolates in this study had base differences with *C. juglandicola* (ITS: 1 bp; *chs-1*: 1 bp; *tub2*: 1 bp). Our strains had base differences in *gapdh* (3 bp) compared with *C. juglandium*. Our strains were morphologically similar to *C. gloeosporioides*. Therefore, we identified all four strains as *C. gloeosporioides*. In addition, based on the phylogenetic tree and the lack of sequence variation, we regarded *C. juglandium*, *C. juglandicola*, and *C. peakense* as synonyms of *C. gloeosporioides*. A detailed explanation for the taxonomic treatment of this section is provided in the discussion.

***Colletotrichum godetiae*** Neerg., Friesia 4 (1–2): 72. 1950.

New proposed synonyms.

*Colletotrichum americanum* M. Zapata et al. Mycological Progress 23: 28. 2024.

*Description*: See Damm et al. [9].

*Material examined*: CHINA, Shanxi Province, Ankang City, Hanbin District, Cigou Town, Er Lang Village, collected from diseased leaves of *Juglans regia*, 108°57′57″ E, 32°55′42″ N, 14 June 2024, Xinlei Fan (living culture CFCC 72574 and CFCC 72575). *ibid*. 108°57′52″ E, 32°55′40″ N, 14 June 2024, Xinlei Fan (living culture LFPR 10001). CHINA, Guizhou Province, Bijie City, Dafang County, Jinhai Lake Wetland Park, collected from diseased leaves of *Calystegia hederacea*, 105°26′31″ E, 27°12′44″ N, 19 June 2024 Weishan Zhang (living culture LFPR 10002).

*Notes*: In the phylogenetic tree, the two strains in this study formed a closely related clade with *C. americanum* and *C. godetiae* (BI/ML = 0.99/95) (Figure 2). Our two strains had base differences with *C. godetiae* (ITS: 1 bp; *gapdh*: 1 bp). However, our isolates had no base differences in other gene fragments with *C. godetiae*. Therefore, we identified the two strains in this study as *C. godetiae*. *C. americanum* (RGM 3380T and RGM 3407) and *C. godetiae* CBS 862.70 had no base differences and morphologically similar. Based on this, we regarded *C. americanum* as a synonym of *C. godetiae*. A detailed explanation for the taxonomic treatment of this section is provided in the discussion.

***Colletotrichum karsti*** Y.L. Yang, Z.Y. Liu, K.D. Hyde & L. Cai, Cryptogonia Mycologia 32 (3): 241. 2011.

*Description*: See Zhang et al. [7].

*Material examined*: CHINA, Fujian Province, Fuzhou City, Jinan District, National Wetland Park, collected from diseased leaves of *Acer rubrum*, 119°19′25″ E, 26°5′6″ N, 20 August 2024, Weishan Zhang (living culture LFPR 10008). CHINA, Guizhou Province, Guiyang City, Aha Lake National Wetland Park, collected from diseased leaves of *Parthenocissus quinquefolia*, 106°36′53″ E, 26°33′52″ N, 27 June 2024, Weishan Zhang (living culture LFPR 10009).

*Notes*: In the phylogenetic tree, the two strains in this study clustered with *C*. *karsti* (BI/ML = 1.00/100) (Figure 3). Therefore, these two isolates were identified as *C. karsti* (Figure 3).

***Colletotrichum nymphaeae*** (Pass.) Aa., Netherlands Journal of Plant Pathology 84 (3): 110. 1978.

*Description*: See Damm et al. [9].

*Material examined*: CHINA, Shanxi Province, Ankang City, Hanbin District, Cigou Town, Er Lang Village, collected from diseased leaves of *Juglans regia*, 108°57′21″ E, 32°55′18″ N, 14 May 2024, Xinlei Fan (living culture CFCC 72576 and CFCC 72577). CHINA, Guizhou Province, Bijie City, Dafang County, Jinhai Lake Wetland Park, collected from diseased leaves of *Senecio scandens*, 105°26′37″ E, 27°12′45″ N, 19 June 2024 Weishan Zhang (living culture LFPR 10007). CHINA, Guizhou Province, Guiyang City, Aha Lake National Wetland Park, collected from diseased leaves of *Fatsia japonica*, 106°36′51″ E, 26°33′59″ N, 27 June 2024, Weishan Zhang (living culture LFPR 10006).

*Notes*: In the phylogenetic tree, our two isolates (CFCC 72576 and CFCC 72577) clustered with *C*. *nymphaeae* (BI/ML = 1.00/98) (Figure 2). Therefore, these two isolates were identified as *C*. *nymphaeae* (Figure 2), representing a new host from China.

***Colletotrichum orchidearum*** Allesch., Rabenh. Krypt.-Fl., Edn 2 (Leipzig) 1 (7): 563. 1903.

*Description*: See Damm et al. [38].

*Material examined*: CHINA, Fujian Province, Fuzhou City, Jinan District, National Wetland Park, collected from diseased leaves of *Anthurium andraeanum*, 119°19′25″ E, 26°5′6″ N, 20 August 2024, Weishan Zhang (living culture LFPR 10029); *ibid*. 119°19′21″ E, 26°5′9″ N, 20 August 2024, Weishan Zhang (living culture LFPR 10030).

*Notes*: In the phylogenetic tree, our two isolates (LFPR 10029 and LFPR 10030) clustered with *C*. *orchidearum* (BI/ML = 1.00/100) (Figure 6). Therefore, these two isolates were identified as *C*. *orchidearum* (Figure 6), representing a novel geographic record for China.

***Colletotrichum plurivorum*** U. Damm., Alizadeh & T. Sato., Studies in Mycology 92: 31. 2018.

New proposed synonyms. *Colletotrichum subplurivorum* Sui et al. Mycosphere 15 (1): 4569–4743. 2024.

*Description*: See Zhang et al. [7].

*Material examined*: CHINA, Fujian Province, Fuzhou City, Jinan District, National Wetland Park, collected from diseased leaves of *Nandina domestica*, 119°19′27″ E, 26°5′6″ N, 20 August 2024, Weishan Zhang (living culture LFPR 10026); *ibid*. collected from diseased leaves of *Spathiphyllum*, 119°19′25″ E, 26°5′8″ N, 20 August 2024, Weishan Zhang (living culture LFPR 10027); *ibid*. collected from diseased leaves of *Megaskepasma erythrochlamys*, 119°19′22″ E, 26°5′6″ N, 20 August 2024, Weishan Zhang (living culture LFPR 10028).

*Notes*: In the phylogenetic tree, the three strains in this study formed a closely related clade with *C*. *plurivorum* and *C*. *subplurivorum* (BI/ML = 1.00/98) (Figure 6). Our strain (LFPR 10028) had base differences with *C*. *plurivorum* (LC8337) (ITS: 1 bp; *tub2*: 5 bp). However, our isolates had no base differences in other gene fragments with *C*. *plurivorum* (LC8337). Therefore, we identified the three strains in this study as *C*. *plurivorum*. *C*. *subplurivorum* (CNUCC 833B-1-1 T) had base differences with *C*. *plurivorum* (LC8337) (ITS: 5 bp; *gapdh*:1 bp; *tub2*: 1 bp). Based on the lack of sequence variation, we regarded *C*. *subplurivorum* as a synonym of *C*. *plurivorum*.

**Figure 14:**
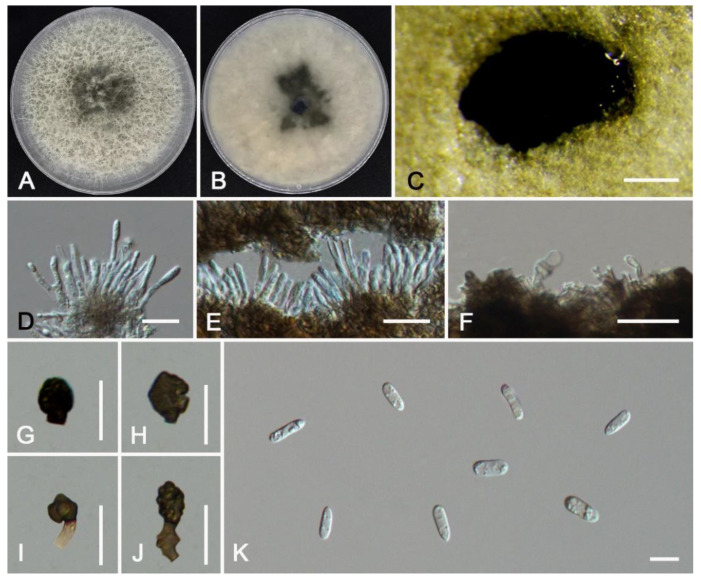
*Colletotrichum siamense* from *Euonymus japonicus* (CFCC 72605): (**A**,**B**) front and reverse colony on PDA (7 d); (**C**) conidial masses formed on PDA; (**D**–**F**) conidiogenous cells; (**G**–**J**) appressoria; (**K**) conidia. Scale bars: (**C**) = 200 μm; (**D**–**M**) = 10 μm.

***Colletotrichum siamense*** Prihast., L. Cai & K.D. Hyde., Fungal Diversity 39: 98. 2009. (Figure 14).

*Description*: See Zhang et al. [7].

*Material examined*: CHINA, Fujian Province, Fuzhou City, Jinan District, National Wetland Park, collected from diseased leaves of *Nandina domestica*, 119°19′27″ E, 26°5′9″ N, 20 August 2024, Weishan Zhang (living culture LFPR 10011); CHINA, Beijing City, Haidian District, Cuihu National Urban Wetland Park, collected from diseased leaves of *Broussonetia papyrifera*, 116°11′60″ E, 40°5′24″ N, 13 October 2024, Weishan Zhang (living culture LFPR 10010). CHINA, Guangdong Province, Dongguan City, Xingtang Xinghua Road, collected from diseased leaves of *Lagerstroemia speciosa*, 113°44′38″ E, 22°59′33″ N, 14 November 2023, Xinlei Fan (HMAS 353995; living culture CFCC 72442); *ibid*. (living culture CFCC 72443). CHINA, Beijing City, Fengtai District, Lotus Pool Park, collected from diseased branch of *Euonymus japonicus*, 2 July 2024, 116°18′49″ E, 39°53′27″ N, Xinlei Fan (BJFC-S2404; living culture CFCC 72605); *ibid*. (BJFC-S2405; living culture CFCC 72606). CHINA, Fujian Province, Fuzhou City, Jinan District, National Wetland Park, collected from diseased leaves of *Chamaedorea pinnatifrons*, 20 August 2024, 119°19′25″ E, 26°5′6″ N, Weishan Zhang (HMAS 353999; living culture CFCC 72430); *ibid*. (living culture CFCC 72431).

*Notes*: In the phylogenetic tree, the eight strains in this study clustered with *C*. *siamense* (Figure 5). The eight strains exhibit morphological similarity to *C. siamense*. Therefore, these eight isolates were identified as *C*. *siamense*.

***Colletotrichum sojae*** Damm., & Alizadeh., Studies in Mycology, 92: 35. 2018.

*Description*: See Sui et al. [2].

*Material examined*: CHINA, Beijing City, Haidian District, Cuihu National Urban Wetland Park, collected from diseased leaves of *Lonicera maackii*, 116°11′59″ E, 40°5′26″ N, 13 October 2024, Weishan Zhang (living culture LFPR 10029); *ibid*. 116°11′60″ E, 40°5′26″ N, 13 October 2024, Weishan Zhang (living culture LFPR 10030).

*Notes*: In the phylogenetic tree, the two strains in this study clustered with *C*. *sojae* (BI/ML = 1.00/100) (Figure 6). Therefore, these two isolates were identified as *C*. *sojae*.

***Colletotrichum spaethianum*** (Allesch.) Damm., P.F. Cannon & Crous, Fungal Diversity. 39: 74. 2009.

*Description*: See Damm et al. [39].

*Material examined*: CHINA, Beijing City, Haidian District, Cuihu National Urban Wetland Park, collected from diseased leaves of *Hosta plantaginea*, 116°11′51″ E, 40°6′5″ N, 18 July 2024, Weishan Zhang (living culture LFPR 10033); *ibid*. collected from diseased leaves of *Malus pumila*, 116°11′50″ E, 40°6′5″ N, 18 July 2024, Weishan Zhang (living culture LFPR 10034); *ibid*. 116°11′50″ E, 40°6′6″ N, 18 July 2024, Weishan Zhang (living culture LFPR 10035).

*Notes*: In the phylogenetic tree, the three strains in this study clustered with *C*. *spaethianum* (BI/ML = 1.00/100) (Figure 7). Therefore, these three isolates were identified as *C*. *spaethianum*.

### 3.3. Correlation Analysis Between Pathogens and Hosts

**Figure 15:**
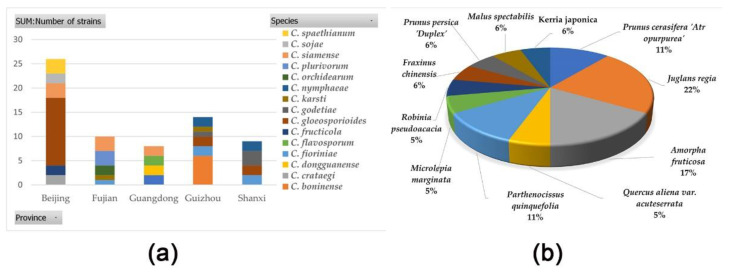
*Colletotrichum* species distribution map: (**a**) stacked bar chart of *Colletotrichum* species distribution by geographic origins; (**b**) pie chart of *Colletotrichum gloeosporioides* distribution on different hosts.

The 67 strains identified in this study belong to 16 *Colletotrichum* species. The species in Beijing are the most diverse (including six *Colletotrichum* species, one of which is a new *Colletotrichum* species) (Figure 15a). The other three new *Colletotrichum* species are all from Guangdong, possibly due to geographic barriers limiting their spread (Figure 15a). *C*. *gloeosporioides* has the highest isolation rate and a wide distribution (Figure 15a). *C*. *gloeosporioides* is concentrated in Beijing, Shaanxi, and Guizhou (Figure 15a). *C*. *gloeosporioides* has a diverse range of hosts, including *Amorpha fruticosa*, *Juglans regia*, *Parthenocissus quinquefolia*, *Quercus aliena* var. *acuteserrata*, and *Prunus cerasifera ‘Atropurpurea’*, showing its broad-host characteristics (Figure 15b). Walnut is dominant among the hosts of *C*. *gloeosporioides*, accounting for 22%, which may be related to its leaf morphology or weaker disease resistance (Figure 15b).

## 4. Discussion

This study has identified 16 *Colletotrichum* species associated with anthracnose diseases in Chinese plants, including three known species within the *C*. *acutatum* complex, four known species within the *C*. *boninense* complex, one new species within the *C*. *destructivum* complex, six new species and three known species within the *C*. *gloeosporioides* complex, three known species within the *C*. *orchidearum*, and one known species within the *C*. *spaethianum*. These findings highlight the remarkable diversity of *Colletotrichum* species causing anthracnose diseases in China.

Jayawardena et al. [40] suggested that *Colletotrichum* species have complex ecological adaptability. The *C. acutatum*, *C. boninense*, and *C. gloeosporioides* complexes exhibit greater species diversity and broader host ranges compared to others [16]. This study also includes ten new host records, such as *Hedera nepalensis* for *C. boninense*, *Aquilaria sinensis* for *C. aquilariae*, *Bauhinia purpurea* for *C. dongguanense*, *Bougainvillea glabra* for *C. flavosporum*, and *Juglans regia* for both *C. fioriniae* and *C. nymphaeae*. The broad host range of these species may be associated with evolutionary processes, ecological niche differentiation, and host interactions.

The research results reveal the ecological characteristics of *Colletotrichum* species from the dimensions of species diversity, geographical distribution, and host preference. In terms of species diversity and geographical differentiation, Beijing has the highest species richness of *Colletotrichum* (including 6 species, one of which is a new species), reflecting that habitat heterogeneity provides a niche basis for fungal differentiation; Guangdong has isolated 3 new species. Combined with the speculation that “geographical barriers limit dispersal”, this confirms that geographical isolation drives the formation of new species—isolated environments reduce gene flow and promote population specialization.

*Colletotrichum gloeosporioides* exhibits prominent ecological dominance: this species has the highest isolation rate and is widely distributed in Beijing, Shaanxi, and Guizhou, reflecting its strong adaptability to different climatic and soil conditions; from the host dimension, its hosts cover 11 plant families, including *Amorpha fruticosa*, *Juglans regia*, *Parthenocissus quinquefolia*, etc., demonstrating broad–spectrum parasitism. However, walnut accounts for 22% of the hosts (significantly higher than other plants). Combined with the speculation that “leaf morphology or weak disease resistance” may be involved, it suggests that the leaf microstructure of walnut (such as stomatal density and cuticle thickness) or defects in its own defense pathways are easily exploited by the pathogen, which provides a core target for walnut disease control. Walnut (*Juglans regia* L.), as a key host, is particularly associated with multiple *Colletotrichum* species. This reveals the susceptibility of walnut due to physiological and environmental factors and the consistency with the strains reported by Li et al. [32]. It further emphasizes the stability and universality of these host–pathogen interactions.

Taxonomic uncertainties were observed within the *C. acutatum*, *C. gloeosporioides*, and *C. orchidearum* species complexes. The strains in this study within the *C. acutatum* complex showed close relationships with *C. americanum* and *C. godetiae*. Although Zapata et al. [41] introduced *C. americanum*, they did not conduct phylogenetic or morphological comparisons with the *C. godetiae* CBS 862.70 strain. Here, we demonstrate that *C. americanum* (RGM 3380T and RGM 3407) and *C. godetiae* CBS 862.70 are morphologically similar and exhibit no significant genetic differences. Therefore, we propose that *C. americanum* is a synonym of *C. godetiae*. The current strains formed a unique clade with *C. gloeosporioides*, *C. juglandicola*, *C. juglandium*, and *C. peakense* within the *C. gloeosporioides* species complex. Previous studies have shown conflicting relationships among these species: Zhang et al. [21] reported that *C. juglandium* and *C. peakense* are closely related to *C. dimorphum*, while Zhang et al. [31] suggested that *C. dimorphum* is a synonym of *C. gloeosporioides*. The current expanded analysis, incorporating additional strains of *C*. *gloeosporioides*, *C. juglandicola*, *C. juglandium*, and *C. peakense,* revealed that they consistently clustered into a stable clade (Figure 5). These three species exhibited less than 1% sequence divergence and shared overlapping morphological features. Based on these findings, we propose that *C. juglandicola*, *C. juglandium*, and *C. peakense* should be considered synonyms of *C. gloeosporioides*. The strains in this study within the *C. orchidearum* complex showed close relationships with *C. subplurivorum* and *C. orchidearum*. Sui et al. [18] introduced *C. subplurivorum*, but they did not conduct a phylogenetic comparison with *C. orchidearum* (LC8337). In this study, we demonstrate that *C. subplurivorum* (CNUCC 833B-1-1T) and *C. orchidearum* (LC8337) exhibit no significant genetic differences. Therefore, we assume that *C. subplurivorum* might be a synonym of *C. orchidearum*.

The identification of Colletotrichum species mostly relies on multigene phylogenetic analysis combined with morphological identification. With the development of molecular techniques, the application of high-throughput sequencing technology has also provided new means for the identification of Colletotrichum species. For example, Liu et al. (2022) [16] conducted high-throughput sequencing of Colletotrichum samples, generating and assembling whole-genome sequences for 30 new species and 18 known species. This demonstrates the great potential of high-throughput sequencing technology in fungal taxonomy. Moreover, although these species were isolated from diseased samples, their exact pathogenic mechanisms remain unclear. Therefore, further research is crucial for elucidating the pathogenic mechanisms of these pathogens.

## Figures and Tables

**Table 1:** Gene fragments and the PCR thermal cycle program used in this study.

Locus	PCR Primers	PCR: Thermal Cycles: (Annealing Temp. in Bold)	Reference
ITS	ITS1/ITS4	(95 °C: 30 s, **51 °C**: 30 s, 72 °C: 1 min) × 35 cycles	[25]
*gapdh*	GDF1/GDR1	(95 °C: 30 s, **58 °C**: 30 s, 72 °C: 1 min) × 35 cycles	[26]
*chs-1*	CHS-79F/CHS-345R	(95 °C: 30 s, **58 °C**: 30 s, 72 °C: 1 min) × 35 cycles	[27]
*act*	ACT-512F/ACT-783R	(95 °C: 45 s, **55 °C**: 45 s, 72 °C: 1 min) × 35 cycles	[28]
*tub2*	T1/Bt-2b	(95 °C: 30 s, **55 °C**: 30 s, 72 °C: 1 min) × 35 cycles	[29]
*his3*	CYLH3F/ CYLH3R	(95 °C: 30 s, **58 °C**: 30 s, 72 °C: 1 min) × 35 cycles	[30]
*cal*	CL1C/CL2C	(95 °C: 30 s, **54 °C**: 20 s, 72 °C: 1 min) × 35 cycles	[12,16]

## Data Availability

All sequence data are available in NCBI GenBank following the accession numbers in the manuscript.

## Supplementary Materials

The following supporting information can be downloaded at: https://www.mdpi.com/article/10.3390/jof11110781/s1, Figure S1. Phylogram of *Colletotrichum acutatum* resulting from a maximum likelihood analysis based on ITS gene. Figure S2. Phylogram of *Colletotrichum acutatum* resulting from a maximum likelihood analysis based on *gapdh* gene. Figure S3. Phylogram of *Colletotrichum acutatum* resulting from a maximum likelihood analysis based on *chs-1* gene. Figure S4. Phylogram of *Colletotrichum acutatum* resulting from a maximum likelihood analysis based on *act* gene. Figure S5. Phylogram of *Colletotrichum acutatum* resulting from a maximum likelihood analysis based on *tub2* gene. Figure S6. Phylogram of *Colletotrichum acutatum* resulting from a maximum likelihood analysis based on *his3* gene. Figure S7. Phylogram of *Colletotrichum boninense* resulting from a maximum likelihood analysis based on ITS gene. Figure S8. Phylogram of *Colletotrichum boninense* resulting from a maximum likelihood analysis based on *gapdh* gene. Figure S9. Phylogram of *Colletotrichum boninense* resulting from a maximum likelihood analysis based on *chs-1* gene. Figure S10. Phylogram of *Colletotrichum boninense* resulting from a maximum likelihood analysis based on *act* gene. Figure S11. Phylogram of *Colletotrichum boninense* resulting from a maximum likelihood analysis based on *tub2* gene. Figure S12. Phylogram of *Colletotrichum boninense* resulting from a maximum likelihood analysis based on *his3* gene. Figure S13. Phylogram of *Colletotrichum destructivum* resulting from a maximum likelihood analysis based on ITS gene. Figure S14. Phylogram of *Colletotrichum destructivum* resulting from a maximum likelihood analysis based on *gapdh* gene. Figure S15. Phylogram of *Colletotrichum destructivum* resulting from a maximum likelihood analysis based on *chs-1* gene. Figure S16. Phylogram of *Colletotrichum destructivum* resulting from a maximum likelihood analysis based on *act* gene. Figure S17. Phylogram of *Colletotrichum destructivum* resulting from a maximum likelihood analysis based on *tub2* gene. Figure S18. Phylogram of *Colletotrichum destructivum* resulting from a maximum likelihood analysis based on *his3* gene. Figure S19. Phylogram of *Colletotrichum gloeosporioides* resulting from a maximum likelihood analysis based on ITS gene. Figure S20. Phylogram of *Colletotrichum gloeosporioides* resulting from a maximum likelihood analysis based on *gapdh* gene. Figure S21. Phylogram of *Colletotrichum gloeosporioides* resulting from a maximum likelihood analysis based on *chs-1* gene. Figure S22. Phylogram of *Colletotrichum destructivum* resulting from a maximum likelihood analysis based on *act* gene. Figure S23. Phylogram of *Colletotrichum gloeosporioides* resulting from a maximum likelihood analysis based on *tub2* gene. Figure S24. Phylogram of *Colletotrichum gloeosporioides* resulting from a maximum likelihood analysis based on *cal* gene. Figure S25. Phylogram of *Colletotrichum orchidearum* resulting from a maximum likelihood analysis based on ITS gene. Figure S26. Phylogram of *Colletotrichum orchidearum* resulting from a maximum likelihood analysis based on *gapdh* gene. Figure S27. Phylogram of *Colletotrichum orchidearum* resulting from a maximum likelihood analysis based on *tub2* gene. Figure S28. Phylogram of *Colletotrichum spaethianum* resulting from a maximum likelihood analysis based on ITS gene. Figure S29. Phylogram of *Colletotrichum spaethianum* resulting from a maximum likelihood analysis based on *gapdh* gene. Figure S30. Phylogram of *Colletotrichum spaethianum* resulting from a maximum likelihood analysis based on *tub2* gene. Table S1: GenBank accession numbers of the sequences used in the phylogenetic analyses of *C*. *acutatum* species in this study; Table S2: GenBank accession numbers of the sequences used in the phylogenetic analyses of *C. boninense* species complex in this study; Table S3: GenBank accession numbers of the sequences used in the phylogenetic analyses of *C. destructivum* species complex in this study; Table S4: GenBank accession numbers of the sequences used in the phylogenetic analyses of *C. gloeosporioides* species complex in this study; Table S5: GenBank accession numbers of the sequences used in the phylogenetic analyses of *C. orchidearum* species complex in this study; Table S6: GenBank accession numbers of the sequences used in the phylogenetic analyses of *C. spaethianum* species complex in this study.
