# Supplementary material for: Diversity and Distribution of Colletotrichum Species Causing Anthracnose in China
Source: J Fungi (Basel). 2025 Oct 30;11(11):781. doi: 10.3390/jof11110781 (PMC12653916; doi:10.3390/jof11110781)
Supplement: Supplementary file 1 [file jof-11-00781-s001.zip › Table S1.pdf]

Supplementary Table S1 GenBank accession numbers of the sequences used in the phylogenetic analyses of *Colletotrichum acutatum* species complex in this study.

Species name	Strains1	Host	Country	ITS	*gapdh*	*chs-1*	*act*	*tub2*	*his3*
C. abscissum	COAD 1877 T	*Citrus sinensis* cv. *Pera*	Brazil	KP843126	KP843129	KP843132	KP843141	KP843135	KP843138
C. acerbum	CBS 128530 = ICMP 12921 = PRJ 1199.3 T	*Malus domestica*	New Zealand	JQ948459	JQ948790	JQ949120	JQ949780	JQ950110	JQ949450
C. acutatum	CBS 112996 = ATCC 56816 =STE-U 5292 T	*Carica papaya*	Australia	JQ005776	JQ948677	JQ005797	JQ005839	JQ005860	JQ005818
C. arboricola	CBS 144795 = SAG 53350-12 T	*Fuchsia magellanica*	Chile	MH817944	MH817950	/	MH817956	MH817962	/
C. australe	CBS 116478 = HKUCC 2616 T	*Trachycarpus fortunei*	South Africa	JQ948455	JQ948786	JQ949116	JQ949776	JQ950106	JQ949446
C. bannaense	YNML 52 = CGMCC 3.18887 T	*Hevea brasiliensis*	China	MG209638	MG242006	MG241996	MG242002	MG209660	/
C. brisbanense	CBS 292.67 = DPI 11711 T	*Capsicum annuum*	Australia	JQ948291	JQ948621	JQ948952	JQ949612	JQ949942	JQ949282
C. cairnsense	BRIP 63642 T	*Capsicum annuum*	Australia	KU923672	KU923704	KU923710	KU923716	KU923688	KU923722
C. carthami	SAPA 100011	*Carthamus tinctorium*	Japan	AB696998	/	/	/	AB696992	/
C. chrysalidocarpi	ZHKUCC 23-0848	*Chrysalidocarpus lutescens*	China	OR287501	OR493925	OR493908	OR493891	OR453377	/
C. chrysalidocarpi	ZHKUCC 23-0847 T	*Chrysalidocarpus lutescens*	China	OR287500	OR493924	OR493907	OR493890	OR453376	/
C. chrysanthemi	IMI 364540 = CPC 18930	*Chrysanthemu mcoronarium*	China	JQ948273	JQ948603	JQ948934	JQ949594	JQ949924	JQ949264
C. cosmi	CBS 853.73 = PD 73/856 T	*Cosmos* sp.	Netherlands	JQ948274	JQ948604	JQ948935	JQ949595	JQ949925	JQ949265
C. costaricense	CBS 330.75 T	*Coffea arabica* cv. *Typica, berry*	Costa Rica	JQ948180	JQ948510	JQ948841	JQ949501	JQ949831	JQ949171
C. cuscutae	IMI 304802 = CPC 18873 T	*Cuscuta* sp.	Dominica	JQ948195	JQ948525	JQ948856	JQ949516	JQ949846	JQ949186
C. eriobotryae	GLMC 1935 T	*Eriobotrya japonica*	China, Taiwan	MF772487	MF795423	MN191653	MN191648	MF795428	/
C. filicis	CBS 101611 T	unidentified fern (Pteridophyta)	Costa Rica	JQ948196	JQ948526	JQ948857	JQ949517	JQ949847	JQ949187
C. fioriniae	CBS 128517 = ARSEF 10222 = ERL 1257 = EHS 58 T	*Fiorinia externa*	USA	JQ948292	JQ948622	JQ948953	JQ949613	JQ949943	JQ949283
C. fioriniae	ATCC 12097 = CPC 19392	*Rhododendron* sp.	USA	JQ948307	JQ948637	JQ948968	JQ949628	JQ949958	JQ949298

Supplementary Table 1 Continued.

Species name	Strains1	Host	Country	ITS	*gapdh*	*chs-1*	*act*	*tub2*	*his3*
C. fioriniae	CBS 167.86	*Myriophyllum spicatum*	USA	JQ948324	JQ948654	JQ948985	JQ949645	JQ949975	JQ949315
C. fioriniae	CBS 200.35	*Rubus* sp.	USA	JQ948293	JQ948623	JQ948954	JQ949614	JQ949944	JQ949284
C. fioriniae	CBS 293.67 = DPI 13120	*Persea americana*	Australia	JQ948310	JQ948640	JQ948971	JQ949631	JQ949961	JQ949301
C. fioriniae	CBS 786.86	*Malus sylvestris*	Italy	JQ948303	JQ948633	JQ948964	JQ949624	JQ949954	JQ949294
C. fioriniae	CBS 112995 = STE-U 5287	*Malus domestica*	USA	JQ948298	JQ948628	JQ948959	JQ949619	JQ949949	JQ949289
C. fioriniae	CNUCC 762B-1- 1	*Camellia oleifera*	China	PP809343	PP832132	PP843066	PP824760	PP832211	PP832172
C. fioriniae	CNUCC 839A-2- 1	*Camellia oleifera*	China	PP809348	PP832137	PP843071	PP824765	PP832216	PP832177
C. fioriniae	CNUCC 90B-1-1	*Camellia sinensis*	China	PP809349	PP832138	PP843072	PP824766	PP832217	PP832178
C. fioriniae	CNUCC 916-1-1	*Camellia sinensis*	China	PP809352	PP832141	PP843075	PP824769	PP832220	PP832181
C. fioriniae	CNUCC 957-2-1	*Paulownia fortunei*	China	PP809350	PP832139	PP843073	PP824767	PP832218	PP832179
C. fioriniae	**CFCC 72578**	***Juglans regia***	**China**	**PV524691**	**PV573481**	**PV573513**	**PV573542**	**PV573573**	**PV573603**
C. fioriniae	**CFCC 72579**	***Juglans regia***	**China**	**PV524692**	**PV573482**	**PV573514**	**PV573543**	**PV573574**	**PV573604**
C. fioriniae	**LFPR 10003**	***Ulmus parvifolia***	**China**	**PV878364**	**PV975937**	/	/	**PV975904**	/
C. fioriniae	**LFPR 10004**	***Laurus nobilis***	**China**	**PV878365**	**PV975938**	/	/	/	/
C. fioriniae	**LFPR 10005**	***Ulmus parvifolia***	**China**	**PV878366**	**PV975939**	/	/	**PV975905**	/
C. godetiae	CBS 133.44 T	*Clarkia hybrida* cv. *Kelvon*	Denmark	JQ948402	JQ948733	JQ949063	JQ949723	JQ950053	JQ949393
C. godetiae	CBS 796.72	*Glory Aeschynomene virginica*	USA	JQ948407	JQ948738	JQ949068	JQ949728	JQ950058	JQ949398
C. godetiae	CBS 126512 = PD 88/958	Bonzai, sunken	Netherlands	JQ948412	JQ948743	JQ949073	JQ949733	JQ950063	JQ949403
C. godetiae	IMI 345026 = CPC 18882	*Fragaria × ananassa*	Spain	JQ948424	JQ948755	JQ949085	JQ949745	JQ950075	JQ949415
C. godetiae	CBS 131332	*Agrimonia eupatoria*	Austria	JQ948429	JQ948760	JQ949090	JQ949750	JQ950080	JQ949420

Supplementary Table 1 Continued.

Species name	Strains1	Host	Country	ITS	*gapdh*	*chs-1*	*act*	*tub2*	*his3*
C. godetiae	CBS 862.70	*Sambucus nigra*	Netherlands	JQ948437	JQ948768	JQ949098	JQ949758	JQ950088	JQ949428
C. godetiae	CBS 129911 = CPC 15124	*Podocarpus sp.*	South Africa	JQ948434	JQ948765	JQ949095	JQ949755	JQ950085	JQ949425
C. godetiae	CBS 126503 = PD 88/859 = BBA 70342	*Fragaria × ananassa*	UK	JQ948420	JQ948751	JQ949081	JQ949741	JQ950071	JQ949411
C. godetiae	CBS 130251 = OL 10 = IMI 398854	*Olea europaea*	Italy	JQ948413	JQ948744	JQ949074	JQ949734	JQ950064	JQ949404
C. godetiae	CBS 130252 = IMI 398855 = OL 20	*Olea europaea*	Italy	JQ948414	JQ948745	JQ949075	JQ949735	JQ950065	JQ949405
C. godetiae	CBS 131331	*Juglans regia, leaf spot*	Austria	JQ948404	JQ948735	JQ949065	JQ949725	JQ950055	JQ949395
C. godetiae	CNUCC 354-2-1	*Camellia sinensis*	China	PP809330	PP832120	PP843053	PP824747	PP832198	PP832159
C. godetiae	CNUCC 363A-1- 1	*Ilex cornuta*	China	PP809332	PP832121	PP843055	PP824749	PP832200	PP832161
C. godetiae	CNUCC 456-2-1	*Lespedeza bicolor*	China	PP809334	PP832123	PP843057	PP824751	PP832202	PP832163
C. godetiae	**CFCC 72574**	***Juglans regia***	**China**	**PV524689**	**PV573479**	**PV573511**	/	**PV573571**	/
C. godetiae	**CFCC 72575**	***Juglans regia***	**China**	**PV524690**	**PV573480**	**PV573512**	/	**PV573572**	/
C. godetiae	**LFPR 10001**	***Juglans regia***	**China**	**PV878362**	**PV975935**	/	/	/	/
C. godetiae	**LFPR 10002**	***Calystegia hederacea***	**China**	**PV878363**	**PV975936**	/	/	**PV975903**	/
C. godetiae* syn. *C. americanum	**RGM 3380 = CCCT 23.24 T**	***Drimys winteri***	**Chile**	**OR644563**	**OR644970**	**OR645022**	**OR645076**	**OR645128**	**OR659700**
C. godetiae* syn. *C. americanum	**RGM 3407**	***Embothrium coccineum***	**Chile**	**OR644564**	**OR644971**	**OR645023**	**OR645077**	**OR645129**	**OR659701**
C. guajavae	IMI 350839 T	*Psidium guajava*	India	JQ948270	JQ948600	JQ948931	JQ949591	JQ949921	JQ949261
C. indonesiense	CBS 127551 T	*Eucalyptus* sp.	Indonesia	JQ948288	JQ948618	JQ948949	JQ949609	JQ949939	JQ949279
C. javanense	CBS 144963a T	*Capsicum annuum*	Indonesia	MH846576	MH846572	MH846573	MH846575	MH846574	MH846571
C. johnstonii	CBS 128532 T	*Solanum lycopersicum*	New Zealand	JQ948444	JQ948775	JQ949105	JQ949765	JQ950095	JQ949435
C. kinghornii	CBS 198.35 T	*Phormium* sp.	UK	JQ948454	JQ948785	JQ949115	JQ949775	JQ950105	JQ949445

Supplementary Table 1 Continued.

Species name	Strains1	Host	Country	ITS	*gapdh*	*chs-1*	*act*	*tub2*	*his3*
C. kniphofiae	CBS 143496 T	*Kniphofia uvaria*	UK	MH107884	MH107998	MH107990	MH107975	MH108037	/
C. kniphofiae	CPC 30168	*Kniphofia uvaria*	UK	MH107885	/	/	/	/	/
C. laticiphilum	CBS 112989 = IMI 383015 = STE-U 5303 T	*Hevea brasiliensis*	India	JQ948289	JQ948619	JQ948950	JQ949610	JQ949940	JQ949280
C. lauri	MFLUCC 17-0205 = IT 2505 1a T	*Laurus nobilis*	Italy	KY514347	KY514344	KY514341	KY514338	KY514350	/
C. limetticola	CBS 114.14	*Citrus aurantifolia*	USA, Florida	JQ948193	JQ948523	JQ948854	JQ949514	JQ949844	JQ949184
C. lupini	CBS 109225 T	*Lupinus albus*	Ukraine	JQ948155	JQ948485	JQ948816	JQ949476	JQ949806	JQ949146
C. melonis	CBS 159.84 T	*Cucumis melo*	Brazil	JQ948194	JQ948524	JQ948855	JQ949515	JQ949845	JQ949185
C. miaoliense	NTUCC 20-001-1 T	*Fragaria × ananassa*	China, Taiwan	MK908419	MK908470	MK908522	MK908573	MK908624	/
C. nagasakiense	TAP19T021 T	*loquat*	Japan	LC718406	LC722603	LC722646	LC722728	LC722769	LC722687
C. nymphaeae	CBS 515.78	*Nymphaea alba*	Netherlands	JQ948197	JQ948527	JQ948858	JQ949518	JQ949848	JQ949188
C. nymphaeae	CBS 126504 = C 105	*Fragaria × ananassa*	South Africa	JQ948265	JQ948595	JQ948926	JQ949586	JQ949916	JQ949256
C. nymphaeae	CBS 112202	*Fragaria* sp.	Spain	JQ948234	JQ948564	JQ948895	JQ949555	JQ949885	JQ949225
C. nymphaeae	CBS 173.51	*Mahonia aquifolium*	Italy	JQ948200	JQ948530	JQ948861	JQ949521	JQ949851	JQ949191
C. nymphaeae	CBS 119294 = MEP 1534	*Leucaena* sp.	Mexico	JQ948205	JQ948535	JQ948866	JQ949526	JQ949856	JQ949196
C. nymphaeae	IMI 360386 = CPC 18925	*Pelargonium graveolens*	India	JQ948206	JQ948536	JQ948867	JQ949527	JQ949857	JQ949197
C. nymphaeae	**CFCC 72576**	***Juglans regia***	**China**	**PV524693**	**PV573483**	**PV573515**	**PV573544**	**PV573575**	**PV573605**
C. nymphaeae	**CFCC 72577**	***Juglans regia***	**China**	**PV524694**	**PV573484**	**PV573516**	**PV573545**	**PV573576**	**PV573606**
C. nymphaeae	**LFPR 10006**	***Fatsia japonica***	**China**	**PV878367**	**PV975940**	**/**	**/**	**PV975906**	**/**
C. nymphaeae	**LFPR 10007**	***Senecio scandens***	**China**	**PV878368**	**PV975941**	**/**	**/**	**PV975907**	**/**

Supplementary Table 1 Continued

Species name	Strains1	Host	Country	ITS	*gapdh*	*chs-1*	*act*	*tub2*	*his3*
C. paranaense	CBS 134729 T	*Malus domestica*	Paraná	KC204992	KC205026	KC205043	KC205077	KC205060	KC205004
C. paxtonii	IMI 165753 = CPC 18868 T	*Musa* sp.	Saint Lucia	JQ948285	JQ948615	JQ948946	JQ949606	JQ949936	JQ949276
C. pedunculi	MYJ 201 = BCRC FU31310 T	*Mangifera indica*	China	MN809388	MN820684	MN820676	MN820668	MN810342	MN820692
C. perseicola	RGM 3376 = CCCT 23.27 T	*Persea lingue*	Chile	OR644585	OR644992	OR645045	OR645098	OR645150	OR659723
C. phormii	CBS 118194 = AR 3546	*Phormium* sp.	Germany	JQ948446	JQ948777	JQ949107	JQ949767	JQ950097	JQ949437
C. pyricola	CBS 128531 = ICMP 12924 =PRJ 977.1 T	*Pyrus communis*	New Zealand	JQ948445	JQ948776	JQ949106	JQ949766	JQ950096	JQ949436
C. orchidophilum	CBS 632.80 T	*Dendrobium* sp.	USA	JQ948151	JQ948481	JQ948812	JQ949472	JQ949802	JQ949142
C. orchidophilum	CBS 631.80	*Ascocenda* sp.	USA	JQ948152	JQ948482	JQ948813	JQ949473	JQ949803	JQ949143
C. rhombiforme	CBS 129953 = PT 250 = RB 011 T	*Olea europaea*	Portugal	JQ948457	JQ948788	JQ949118	JQ949778	JQ950108	JQ949448
C. roseum	CBS 145754 T	*Lapageria rosea*	Chile	MK903611	MK903603	/	MK903604	MK903607	/
C. salicis	CBS 607.94	*Salix* sp.	Netherlands	JQ948460	JQ948791	JQ949121	JQ949781	JQ950111	JQ949451
C. schimae	LC 13880 = NN 046984 T	*Schima* sp.	China	MZ595885	MZ664105	MZ799347	MZ66418	MZ674003	MZ673905
C. schimae	LC 13881 = NN 047247	*Schima* sp.	China	MZ595887	MZ664106	MZ799348	MZ664185	MZ674005	MZ673907
C. scovillei	CBS 126529 = PD 94/921-3 = BBA 70349 T	*Capsicum* sp.	Indonesia	JQ948267	JQ948597	JQ948928	JQ949588	JQ949918	JQ949258
C. simmondsii	CBS 122122 = BRIP 28519 T	*Carica papaya*	Australia	JQ948276	JQ948606	JQ948937	JQ949597	JQ949927	JQ949267
C. sloanei	IMI 364297 = CPC 18929 T	*Theobroma cacao*	Malaysia	JQ948287	JQ948617	JQ948948	JQ949608	JQ949938	JQ949278
C. subsalicis	CQ 1168 = LC 13863 T	*Populus alba*	China	MZ852849	/	MZ799346	MZ664128	MZ673953	MZ673836
C. tamarilloi	CBS 129814 = T.A.6 T	*Solanum betaceum*	Colombia	JQ948184	JQ948514	JQ948845	JQ949505	JQ949835	JQ949175
C. walleri	CBS 125472 = BMT(HL) 19 T	*Coffea* sp.	Vietnam	JQ948275	JQ948605	JQ948936	JQ949596	JQ949926	JQ949266
C. wanningense	CGMCC 3.18936 T	Rubber tree	China	MG830462	MG830318	MG830302	MG830270	MG830286	/

Supplementary Table 2 GenBank accession numbers of the sequences used in the phylogenetic analyses of *C. boninense* species complex in this study.

Species name	Strains1	Host	Country	ITS	*gapdh*	*chs-1*	*act*	*tub2*	*his3*
C. annellatum	CBS 129826 = CH 1 T	*Hevea indica*	Colombia	JQ005222	JQ005309	JQ005396	JQ005570	JQ005656	JQ005483
C. beeveri	CBS 128527 = ICMP 18594 T	*Brachyglottis repanda*	New Zealand	JQ005171	JQ005258	JQ005345	JQ005519	JQ005605	JQ005432
C. boninense	CBS 123755 = MAFF 305972 T	*Crinum asiaticum* var.	Japan	JQ005153	JQ005240	JQ005327	JQ005501	JQ005588	JQ005414
C. boninense	CBS 123756 = MAFF 306094	*sinicum Crinum asiaticum* var.	Japan	JQ005154	JQ005241	JQ005328	JQ005502	JQ005589	JQ005415
C. boninense	CBS 128547 = ICMP 10338	*Camellia* sp.	New Zealand	JQ005159	JQ005246	JQ005333	JQ005507	JQ005593	JQ005420
C. boninense	MAFF 306162 = ICMP 18596	*Crinum asiaticum* var. *sinicum*	Japan	JQ005155	JQ005242	JQ005329	JQ005503	/	JQ005416
C. boninense	CBS 128526 = ICMP 18591	*Dacrycarpus dacrydioides*	New Zealand	JQ005162	JQ005249	JQ005336	JQ005510	JQ005596	JQ005423
C. boninense	CBS 112115 = STE-U 2966	*Leucospermum* sp.	Australia	JQ005160	JQ005247	JQ005334	JQ005508	JQ005594	JQ005421
C. boninense	CBS 129831 = STE-U 2965	*Leucospermum* sp.	Australia	JQ005161	JQ005248	JQ005335	JQ005509	JQ005595	JQ005422
C. boninense	CBS 128549 = ICMP 15444	*Solanum betaceum*	New Zealand	JQ005156	JQ005243	JQ005330	JQ005504	JQ005590	JQ005417
C. boninense	CBS 128506 = ICMP 12950	*Solanum lycopersicum*	New Zealand	JQ005157	JQ005244	JQ005331	JQ005505	JQ005591	JQ005418
C. boninense	CBS 128546 = ICMP 18595	*Tecomanthe speciosa*	New Zealand	JQ005158	JQ005245	JQ005332	JQ005506	JQ005592	JQ005419
C. boninense	**CFCC 72426**	***Hedera nepalensis* var. *sinensis***	**China**	**PV524695**	**PV573485**	**PV573517**	**PV573546**	**PV573577**	**PV573607**
C. boninense	**CFCC 72427**	***Hedera nepalensis* var. *sinensis***	**China**	**PV524696**	**PV573486**	**PV573518**	**PV573547**	**PV573578**	**PV573608**
C. boninense	**CFCC 72424**	***Coriaria napalensis***	**China**	**PV524697**	**PV573487**	**PV573519**	**PV573548**	**PV573579**	**PV573609**
C. boninense	**CFCC 72425**	***Coriaria napalensis***	**China**	**PV524698**	**PV573488**	**PV573520**	**PV573549**	**PV573580**	**PV573610**
C. boninense	**CFCC 72422**	***Fatsia japonica***	**China**	**PV524699**	**PV573489**	**PV573521**	**PV573550**	**PV573581**	**PV573611**
C. boninense	**CFCC 72423**	***Fatsia japonica***	**China**	**PV524700**	**PV573490**	**PV573522**	**PV573551**	**PV573582**	**PV573612**
C. brasiliense	CBS 128501 = ICMP 18607 = PAS 12 T	*Passiflora edulis*	Brazil	JQ005235	JQ005322	JQ005409	JQ005583	JQ005669	JQ005496

Supplementary Table 2 Continued

Species name	Strains1	Host	Country	ITS	*gapdh*	*chs-1*	*act*	*tub2*	*his3*
C. brassicicola	CBS 101059 = LYN 16331 T	*Brassica oleracea* var. *gemmifera*	New Zealand	JQ005172	JQ005259	JQ005346	JQ005520	JQ005606	JQ005433
C. bromeliacearum	LC 0951 T	Bromeliad	China	MZ595832	MZ664077	MZ799267	MZ664130	MZ673956	MZ673843
C. camelliae-japonicae	CGMCC 3.18118 = LC 6416 T	*Camellia japonica*	China	KX853165	KX893584	MZ799271	KX893576	KX893580	MZ673859
C. capsicicola	PC159	*Capsicum annuum*	Brazil	OR505853	OR599648	OR599656	OR599652	/	/
C. capsicicola	URM 8822 T	*Capsicum annuum*	Brazil	OR505852	OR599647	OR599655	OR599651	/	/
C. catinaense	CBS 142417 = CPC 27978 T	*Citrus reticulata*	Italy	KY856400	KY856224	KY856136	KY855971	KY856482	KY856307
C. chamaedoreae	LC 13868 = NN 052885 T	*Chamaedorea erumpens*	China	MZ595890	MZ664084	MZ799274	MZ664188	MZ674008	MZ673910
C. chongqingense	CS 0612 (CB6- 1) T	*Camellia sinensis*	China	MG602060	MG602022	MT976117	MT976107	MG602044	/
C. citricola	ZJUC 34 = CBS 134228 = CGMCC 3.15227 T	*Citrus unshiu*	China	KC293576	KC293736	KY856140	KC293616	KC293656	/
C. cliviigenum	CPC 38800 = CBS 146825 T	*Clivia* sp.	South Africa	MZ064415	MZ078178	MZ078161	MZ078143	MZ078260	MZ078180
C. colombiense	CBS 129818 T	*Passiflora edulis*	Colombia	JQ005174	JQ005261	JQ005348	JQ005522	JQ005608	JQ005435
C. condaoense	CBS 134299 T	*Ipomoea pes-caprae*	Vietnam	MH229914	MH229920	MH229926	/	MH229923	MH229927
C. constrictum	CBS 128504 = ICMP 12941 T	*Citrus limon*	New Zealand	JQ005238	JQ005325	JQ005412	JQ005586	JQ005672	JQ005499
C. cymbidiicola	IMI 347923 T	*Cymbidium* sp.	Australia	JQ005166	JQ005253	JQ005340	JQ005514	JQ005600	JQ005427
C. dacrycarpi	CBS 130241 = ICMP 19107 T	*Dacrycarpus dacrydioides*	New Zealand	JQ005236	JQ005323	JQ005410	JQ005584	JQ005670	JQ005497
C. diversum	LC 11292 = CQ 775 T	*Philodendron selloum*	China	MZ595844	MZ664081	MZ799272	MZ664142	MZ673965	MZ673864
C. doitungense	MFLUCC 14- 0128 T	*Dendrobium* sp.	Thailand	MF448524	MH049480	/	MH376385	MH351277	/
C. feijoicola	CBS 144633 T	*Acca sellowiana*	Portugal	MK876413	MK876475	/	MK876466	MK876507	/
C. hippeastri	CBS 125376 = CSSG 1 T	*Hippeastrum vittatum*	China	JQ005231	JQ005318	JQ005405	JQ005579	JQ005665	JQ005492
C. karsti	CGMCC 3.14194 = CORCG 6 T	*Vanda* sp.	China	HM585409	HM585391	HM582023	HM581995	HM585428	/

Supplementary Table 2 Continued

Species name	Strains1	Host	Country	ITS	*gapdh*	*chs-1*	*act*	*tub2*	*his3*
C. karsti	CBS 486.97	*Lupinus albus*, cv. *Lu Blanc*	Germany	JQ005182	JQ005269	JQ005356	JQ005530	JQ005616	JQ005443
C. karsti	CBS 861.72	*Bombax aquaticum*	Brazil	JQ005184	JQ005271	JQ005358	JQ005532	JQ005618	JQ005445
C. karsti	CBS 110779	*Eucalyptus grandis*	South Africa	JQ005198	JQ005285	JQ005372	JQ005546	JQ005632	JQ005459
C. karsti	CBS 124951	*Theobroma*	Panama	JQ005180	JQ005267	JQ005354	JQ005528	JQ005614	JQ005441
C. karsti	CBS 125468	*Coffea* sp.	Vietman	JQ005197	JQ005284	JQ005371	JQ005545	JQ005631	JQ005458
C. karsti	CBS 127591	*Sapium integerrimium*	Australia	JQ005186	JQ005273	JQ005360	JQ005534	JQ005620	JQ005447
C. karsti	CBS 129834	*Musa* sp.	Mexico	JQ005176	JQ005263	JQ005350	JQ005524	JQ005610	JQ005437
C. karsti	CBS 130235	*Gossypium hirsutum*	Germany	JQ005190	JQ005277	JQ005364	JQ005538	JQ005624	JQ005451
C. karsti	**LFPR 10008**	***Acer rubrum***	**China**	**PV878369**	**PV975942**	/	/	**PV975908**	/
C. karsti	**LFPR 10009**	***Parthenocissus quinquefolia***	**China**	**PV878370**	**PV975943**	/	/	**PV975909**	/
C. laurosilvaticum	RGM 3406 = CCCT 23.11 T	*Laurelia sempervirens*	Chile	OR644582	OR644989	OR645042	OR645095	OR645147	OR659720
C. laurosilvaticum	RGM 3086 = CCCT 23.06		Chile	OR644581	OR644988	OR645041	OR645094	OR645146	OR659719
C. limonicola	CBS 142410 = CPC 31141	*Citrus limon*	Malta, Gozo	KY856472	KY856296	KY856213	KY856045	KY856554	KY856388
C. novae-zelandiae	CBS 128505 = ICMP 12944	*Capsicum annuum*	New Zealand	JQ005228	JQ005315	JQ005402	JQ005576	JQ005662	JQ005489
C. oncidii	CBS 129828	*Oncidium* sp.	Germany	JQ005169	JQ005256	JQ005343	JQ005517	JQ005603	JQ005430
C. palki	RGM 3055 = CCCT 23.04 T	*Cestrum parqui*	Chile	OR644584	OR644991	OR645044	OR645097	OR645149	OR659722
C. parsonsiae	CBS 128525 = ICMP 18590 T	*Parsonsia capsularis*	New Zealand	JQ005233	JQ005320	JQ005407	JQ005581	JQ005667	JQ005494
C. pernambucoense	URM 8821 T	*Capsicum annuum*	Brazil	OR505854	OR599649	OR599657	OR599653	/	/
C. pernambucoense	PC86	*Capsicum annuum*	Brazil	OR505855	OR599650	OR599658	OR599654	/	/
C. petchii	CBS 378.94 T	*Dracaena marginata*	Italy	JQ005223	JQ005310	JQ005397	JQ005571	JQ005657	JQ005484

Supplementary Table 2 Continued

Species name	Strains1	Host	Country	ITS	*gapdh*	*chs-1*	*act*	*tub2*	*his3*
C. phyllanthi	CBS 175.67 = MACS 271 T	*Phyllanthus acidus*	India	JQ005221	JQ005308	JQ005395	JQ005569	JQ005655	JQ005482
C. torulosum	CBS 128544 = ICMP 18586 T	*Solanum melongena*	New Zealand	JQ005164	JQ005251	JQ005338	JQ005512	JQ005598	JQ005425
C. truncatum	CBS 151.35	*Phaseolus lunatus*	USA	GU227862	GU228254	GU228352	GU227960	GU228156	GU228058
C. watphraense	MFLUCC 14- 0123 T	*Dendrobium* sp.	Thailand	MF448523	MH049479	/	MH376384	MH351276	/

Supplementary Table 3 GenBank accession numbers of the sequences used in the phylogenetic analyses of *C. destructivum* species complex in this study.

Species name	Strains1	Host	Country	ITS	*gapdh*	*chs-1*	*act*	*tub2*	*his3*
C. americae-borealis	CBS 136232 T	*Medicago sativa*	USA	KM105224	KM105579	KM105294	KM105434	KM105504	KM105364
C. antirrhinicola	CBS 102189 T	*Antirrhinum majus*	New Zealand	KM105180	KM105531	KM105250	KM105390	KM105460	KM105320
C. atractylodicola	CGMCC 3.18761 = SAUCC 1307 T	*Atractylodes chinensis*	China	KR149280	KR259334	KR259333	KR132243	KU058178	/
C. atractylodicola	SAUCC130721	*Atractylodes chinensis*	China	KU289190	KU289205	KU289200	KU289195	KU289210	/
C. atractylodicola	SAUCC130724	*Atractylodes chinensis*	China	KU289191	KU289206	KU289201	KU289196	KU289211	/
C. bryoniicola	CBS 109849 T	*Bryonia dioica*	Netherlands	KM105181	KM105532	KM105251	KM105391	KM105461	KM105321
C. crataegi	**CFCC 72428 T**	***Crataegus pinnatifida***	**China**	**PV524701**	**PV573491**	**PV573523**	**PV573552**	**PV573583**	**PV573613**
C. crataegi	**CFCC 72429**	***Crataegus pinnatifida***	**China**	**PV524702**	**PV573492**	**PV573524**	**PV573553**	**PV573584**	**PV573614**
C. cunninghamiae	CNUCC 58-57-2 T	*Cunninghamia lanceolata*	China	PP812213	PP831925	PP820670	PP820664	PP831937	PP831931
C. dematium	CBS 125.25	*Eryngium campestre*	France	GU227819	GU228211	GU228309	GU227917	GU228113	GU228015
C. destructivum	CBS 136228	*Trifolium hybridum*	USA	KM105207	KM105561	KM105277	KM105417	KM105487	KM105347
C. fructi	CBS 346.37	*Malus sylvestris*	USA	GU227844	GU228236	GU228334	GU227942	GU228138	GU228040
C. fuscum	CBS 133701	*Digitalis lanata*	Germany	KM105174	KM105524	KM105244	KM105384	KM105454	KM105314
C. higginsianum	IMI 349061 = CPC 19379	*Brassica chinensis*	Trinidad	KM105184	KM105535	KM105254	KM105394	KM105464	KM105324
C. hubeiense	CNUCC 408-1-1 T	*Lindera glauca*	China	PP812212	PP831924	PP820669	PP820663	PP831936	PP831930

Supplementary Table 3 Continued

Species name	Strains1	Host	Country	ITS	*gapdh*	*chs-1*	*act*	*tub2*	*his3*
C. hubeiense	CNUCC 408-1-1- 2	*Lindera glauca*	China	PP812216	PP831928	PP820673	PP820667	PP831940	PP831934
C. kummerowiae	KACC 43776	*Kummerowia striata*	Korea, Hongcheon	OR431672	OR449471	OR449445	OR449419	OR449501	OR449482
C. kummerowiae	KACC 42433	*Kummerowia striata*	Korea, Hoengseong	OR431668	OR449468	OR449442	OR449420	OR449503	OR449485
C. kummerowiae	KACC 42404 T	*Kummerowia striata*	Korea, Hoengseong	OR431667	OR449464	OR449452	OR449411	OR449507	OR449498
C. lentis	CBS 127604 = DAOM 235316 = CT21 T	*Lens culinaris*	Canada	JQ005766	KM105597	JQ005787	JQ005829	JQ005850	JQ005808
C. lini	CBS 172.51	*Linum usitatissimum*	Netherlands	JQ005765	KM105581	JQ005786	JQ005828	JQ005849	JQ005807
C. luanense	CNUCC 157A-4-4 T	*Camellia sinensis*	China	PP812211	PP831923	PP820668	PP820662	PP831935	PP831929
C. neorubicola	CCR144	*Bubus idaeus*	China	MK529906	MK547520	MK547526	MK547523	MN186400	/
C. ocimi	CBS 298.94 T	*Ocimum basilicum*	Italy	KM105222	KM105577	KM105292	KM105432	KM105502	KM105362
C. panacicola	C08048	*Panax ginseng*	China	GU935867	GU935847	/	GU944757	GU935887	/
C. panacicola	YL2-2	*Panax ginseng*	China	MN685228	MN894870	MN894862	MN894854	MN894877	/
C.pisicola	CBS 724.97= LARS 60 T	*Pisum sativum*	USA	KM105172	KM105522	KM105242	KM105382	KM105452	KM105312
C. pleopeltidis	CBS 147082 (CPC 39342) T	*Pleopeltis* sp.	South Africa	MW883412	/	MW890035	MW890024	/	/
C. quercicola	CFCC 57507	*Quercus variabilis*	China	ON692811	ON755053	ON755049	ON755045	ON755057	/
C. shisoi	JCM 31818 T	*Perilla frutescens* var.	Japan	MH660930	MH660931	MH660929	MH660928	MH660932	/
C. tabacum	CPC 18945 = N150 T	*Crispa Nicotiana tabacum*	Canada	KM105204	KM105557	KM105274	KM105414	KM105484	KM105344
C. tabacum	CBS 124249 = MUCL 44942	*Centella asiatica*	Madagascar	KM105206	KM105560	KM105276	KM105416	KM105486	KM105346
C. tanaceti	CBS 132693 = UM01 = BRIP 57314 T	*Tanacetum cinerariifolium*	Ulverstone	JX218228	JX218243	JX259268	JX218238	JX218233	/
C. utrechtense	CBS 130243 T	*Trifolium pratense*	Netherlands	KM105201	KM105554	KM105271	KM105411	KM105481	KM105341
C. utrechtense	CBS 135827	*Trifolium pratense*	Netherlands	KM105202	KM105555	KM105272	KM105412	KM105482	KM105342
C. vignae	CBS 501.97 = LARS 56 T	*Vigna unguiculata*	Nigeria	KM105183	KM105534	KM105253	KM105393	KM105463	KM105323

Supplementary Table 4 GenBank accession numbers of the sequences used in the phylogenetic analyses of *C. gloeosporioides* species complex in this study.

Species name	Strain	Host	Country	ITS	*gapdh*	*chs-1*	*act*	*tub2*	*cal*
C. aenigma	ICMP 18608 T	*Persea americana*	Israel	JX010244	JX010044	JX009774	JX009443	JX010389	JX009683
C. aeschynomenes	ICMP 17673 = ATCC 201874 T	*Aeschynomene virginica*	USA	JX010176	JX009930	JX009799	JX009483	JX010392	JX009721
C. alatae	CBS 304.67 = ICMP 17919 T	*Dioscorea alata*	India	JX010190	JX009990	JX009837	JX009471	JX010383	JX009738
C. alienum	ICMP 12071 T	*Malus domestica*	New Zealand	JX010251	JX010028	JX009882	JX009572	JX010411	JX009654
C. anhuiense	AG 52	*Osmanthus fragrans*	China	OL772737	OL854172	OL854158	OM100918	OL854186	OL854147
C. anhuiense	AG 85 T	*Osmanthus fragrans*	China	OL772736	OL854171	OL854157	OM100917	OL854185	OL854146
C. aotearoa	ICMP 18537 T	*Coprosma* sp.	New Zealand	JX010205	JX010005	JX009853	JX009564	JX010420	JX009611
C. arecicola	CGMCC 3.19667 T	*Areca catechu*	China	MK914635	MK935455	MK935541	MK935374	MK935498	/
C. artocarpicola	MFLUCC 18-1167 T	*Artocarpus heterophyllus*	Thailand	MN415991	MN435568	MN435569	MN435570	MN435567	/
C. asianum	ICMP 18580 = CBS 130418 T	*Coffea arabica*	Thailand	FJ972612	JX010053	JX009867	JX009584	JX010406	FJ917506
C. atlanticum	LM898	*Etlingera elatior*	Brazil	/	MZ264093	/	/	MZ270516	/
C. atlanticum	LM938 T	*Etlingera elatior*	Brazil	/	MZ264107	/	/	MZ270523	/
C. aquilariae	**CFCC 72436 T**	***Aquilaria sinensis***	**China**	**PV524711**	**PV573501**	**PV573532**	**PV573561**	**PV573593**	**PV573623**
C. aquilariae	**CFCC 72437**	***Aquilaria sinensis***	**China**	**PV524712**	**PV573502**	**PV573533**	**PV573562**	**PV573594**	**PV573624**
C. australianum	VPRI 43075 = UMC 002 T	*Citrus sinensis*	Australia	MG572138	MG572127	MW091987	MN442109	MG572149	/
C. avicenniae	MFLUCC23-0289	*Avicennia marina*	Thailand	OR856121	OR886390	OR886402	OR886393	OR886396	/
C. camelliae	CNUCC 257-2-4	*Camellia sinensis*	China	PP843285	PP849785	PP841693	PP922865	PP890425	PP854762
C. camelliae	CNUCC 781B-1-2	*Camellia sinensis*	China	PP843307	PP849807	PP841715	PP922887	PP870977	PP854783
C. cangyuanense	YMF 1.04998	*Ageratina adenophora*	China	OK030865	OK513668	OK513564	OK513604	OK513634	/
C. cangyuanense	YMF 1.05000	*Ageratina adenophora*	China	OK030863	OK513666	OK513562	OK513602	OK513632	/
C. cangyuanense	YMF 1.05001	*Ageratina adenophora*	China	OK030864	OK513667	OK513563	OK513603	OK513633	/

Supplementary Table 4 Continued

Species name	Strain	Host	Country	ITS	*gapdh*	*chs-1*	*act*	*tub2*	*cal*
C. cangyuanense	CNUCC 103-13-3	*Camellia oleifera*	China	PP843243	PP849743	PP841651	PP922823	PP886092	PP854806
C. cangyuanense	CNUCC 104C-16-1	*Camellia oleifera*	China	PP843244	PP849744	PP841652	PP922824	PP886093	PP854721
C. cangyuanense	CNUCC 22-21-2	*Cunninghamia lanceolata*	China	PP843269	PP849769	PP841677	PP922849	PP890409	PP854746
C. cangyuanense	CNUCC 22-23-2	*Cunninghamia lanceolata*	China	PP843270	PP849770	PP841678	PP922850	PP890410	PP854747
C. cangyuanense	CNUCC 22-24-3	*Cunninghamia lanceolata*	China	PP843271	PP849771	PP841679	PP922851	PP890411	PP854748
C. cangyuanense	CNUCC 245-3-4	*Cunninghamia lanceolata*	China	PP843283	PP849783	PP841691	PP922863	PP890423	PP854760
C. cangyuanense	CNUCC 59B-51-4	*Cunninghamia lanceolata*	China	PP843292	PP849792	PP841700	PP922872	PP890430	PP854807
C. cangyuanense	CNUCC 96-7-1	*Cunninghamia lanceolata*	China	PP843260	PP849827	PP841735	PP922907	PP870997	PP854803
C. changpingense	CGMCC 3.17582 = SA 0016 = MFLUCC 15-0022 T	*Fragaria* × *ananass*	China	KP683152	KP852469	KP852449	KP683093	KP852490	/
C. chiangmaiense	MFLUCC 18-0945 T	*Magnolia garrettii*	Thailand	MW346499	MW548592	MW623653	MW655578	/	/
C. chrysophilum	URM 7368 = CMM 4268 T	*Musa* sp.	Brazil	KX094252	KX094183	KX094083	KX093982	KX094285	KX094063
C. ciggaro	ICMP 18539 T	*Olea europaea*	Australia	JX010230	JX009966	JX009800	JX009523	JX010434	JX009635
C. clidemiae	ICMP 18658 T	*Clidemia hirta*	USA, Hawaii	JX010265	JX009989	JX009877	JX009537	JX010438	JX009645
C. cobbittiense	BRIP 66219 T	*Cordyline stricta* × *C. australis*	Australia	MH087016	MH094133	MH094135	MH094134	MH094137	/
C. coffeae-arabicae	PPDU26B	*Coffea arabica*	Saudi Arabia	OR048779	OR050760	OR050742	OR050690	OR050787	/
C. coffeae-arabicae	PPDU29F	*Coffea arabica*	Saudi Arabia	OR048768	OR050749	OR050731	OR050679	OR050776	/
C. coffeae-arabicae	PPDU29F	*Coffea arabica*	Saudi Arabia	OR048768	OR050749	OR050731	OR050679	OR050776	/
C. coffeae-arabicae	PPDU32A	*Coffea arabica*	Saudi Arabia	OR048764	OR050745	OR050727	OR050675	OR050772	/
C. conoides	CGMCC 3.17615 = CAUG 17 = LC 6226 T	*Chili pepper*	China	KP890168	KP890162	KP890156	KP890144	KP890174	KP890150

Supplementary Table 4 Continued

Species name	Strain	Host	Country	ITS	gapdh	chs-1	act	tub2	*cal*
C. cordea	CFCC 70160	*Juglans regia*	China	PP397135	PP425086	PP425045	PP425002	PP425127	/
C. cordea	CFCC 59618 T	*Juglans regia*	China	PP397136	PP425087	PP425046	PP425003	PP425128	/
C. cordylinicola	MFLUCC 09-0551 = ICMP 18579 T	*Cordyline fruticosa*	Thailand	JX010226	JX009975	JX009864	HM470235	JX010440	HM470238
C. cycadis	BRIP 71326a T	*Cycas revoluta*	China	MT439915	MT439919	MT439917	/	MT439921	/
C. cycadis	AQISWA 201901	*Cycas revoluta*	China	MT439916	MT439920	MT439918	/	MT439922	/
C. dongguanense	**CFCC 72438 T**	***Bauhinia purpurea***	**China**	**PV524715**	**PV573505**	**PV573536**	**PV573565**	**PV573597**	**PV573627**
C. dongguanense	**CFCC 72439**	***Bauhinia purpurea***	**China**	**PV524716**	**PV573506**	**PV573537**	**PV573566**	**PV573598**	**PV573628**
C. dracaenigenum	MFLUCC 19-0430 T	*Dracaena* sp.	Thailand	MN921250	MT215577	MT215575	MT313686	/	/
C. endophyticum	MFLUCC 130418 = LC 0324 T	*Pennisetum purpureum*	Thailand	KC633854	KC832854	MZ799261	KF306258	MZ673954	KC810018
C. euonymi	CFCC 55542 T	*Euonymus japonicus*	China	OQ344718	OQ410567	/	/	OQ410547	/
C. euonymi	CFCC 55540	*Euonymus japonicus*	China	OQ344715	OQ410564	/	/	OQ410544	/
C. euonymicola	CFCC 55486 T	*Euonymus japonicus*	China	OQ344723	OQ410572	/	/	OQ410552	/
C. euonymicola	CFCC 55539	*Euonymus japonicus*	China	OQ344724	OQ410573	/	/	OQ410553	/
C. fici-septicae	MFLUCC 20-0166 T	*Ficus septica*	China, Taiwan	MW114367	MW183774	MW177701	MW151585	/	/
C. flavosporum	**CFCC 72442 T**	***Bougainvillea glabra***	**China**	**PV524713**	**PV573503**	**PV573534**	**PV573563**	**PV573595**	**PV573625**
C. flavosporum	**CFCC 72443**	***Bougainvillea glabra***	**China**	**PV524714**	**PV573504**	**PV573535**	**PV573564**	**PV573596**	**PV573626**
C. floscerae	LM891	*Etlingera elatior*	Brazil	/	MZ264092	/	/	MZ270515	/
C. floscerae	LM916 T	*Etlingera elatior*	Brazil	/	MZ264099	/	/	MZ270518	/
C. fructicola	ICMP 18581 = BPDI 16 = CBS 130416 T	*Coffea arabica*	Thailand	JX010165	JX010033	JX009866	FJ907426	JX010405	FJ917508
C. fructicola	ICMP 18646 = CBS 125397 = MTCC 10906	*Tetragastris panamensis*	Panama	JX010173	JX010032	JX009874	JX009581	JX010409	JX009674
C. fructicola	**CFCC 72434**	***Juglans regia***	**China**	**PV524707**	**PV573497**	**PV573528**	**PV573557**	**PV573589**	**PV573619**

Supplementary Table 4 Continued

Species name	Strain	Host	Country	ITS	*gapdh*	*chs-1*	*act*	*tub2*	*cal*
C. fructicola	**CFCC 72435**	***Juglans regia***	**China**	**PV524708**	**PV573498**	**PV573529**	**PV573558**	**PV573590**	**PV573620**
C. fructivorum	Coll 1414 = BPI 884103 = CBS 133125 T	*Vaccinium macrocarpon*	Burling-ton	JX145145	MZ664047	MZ799259	MZ664126	JX145196	/
C. gardeniae	GUCC 12048	*Gardenia jasminoides*	China	OP722989	OP737962	OP715765	OP715800	OP720857	/
C. gardeniae	GUCC 12049 T	*Gardenia jasminoides*	China	OP722995	OP737963	OP715766	OP715801	OP720858	/
C. gloeosporioides	CBS 112999 = IMI 356878 = ICMP 17821	*Citrus sinensis*	Italy	JQ005152	JQ005239	JQ005326	JQ005500	JQ005587	JQ005673
C. gloeosporioides	ICMP 12938	*Citrus sinensis*	New Zealand	JX010147	JX009935	JX009746	JX009560	/	JX009732
C. gloeosporioides	CBS 273.51 = ICMP 19121 T	*Citrus limon*	Italy	JX010148	JX010054	JX009903	JX009558	/	JX009745
C. gloeosporioides	CNUCC 151A-2-2	*Osmanthus fragrans*	China	PP843251	PP849751	PP841659	PP922831	PP886100	PP854728
C. gloeosporioides	CNUCC 191-1-2	*Parthenocissus*	China	PP843258	PP849758	PP841666	PP922838	PP886107	PP854735
C. gloeosporioides	CNUCC 232-2-1	*Cinnamomum camphora*	China	PP843279	PP849779	PP841687	PP922859	PP890419	PP854756
C. gloeosporioides	CNUCC 250-4-3	*Cunninghamia lanceolata*	China	PP843284	PP849784	PP841692	PP922864	PP890424	PP854761
C. gloeosporioides	CNUCC 61-23-1	*Cunninghamia lanceolata*	China	PP843295	PP849795	PP841703	PP922875	PP890433	PP854771
C. gloeosporioides	CNUCC 936-1-1	*Paulownia fortunei*	China	PP843322	PP849822	PP841730	PP922902	PP870992	PP854798
C. gloeosporioides	CNUCC 948-1-2	*Cercis chinensis*	China	PP843324	PP849824	PP841732	PP922904	PP870994	PP854800
C. gloeosporioides	CNUCC 953-1-3	*Cercis chinensis*	China	PP843326	PP849826	PP841734	PP922906	PP870996	PP854802
C. gloeosporioides	LC 3382 = LF 604	*Camellia sinensis*	China	KJ955176	KJ954877	/	KJ954450	KJ955323	KJ954728
C. gloeosporioides	CNUCC 184-3-3	*Diospyros kaki* var. *silvestris*	China	PP843257	PP849757	PP841665	PP922837	PP886106	PP854734
C. gloeosporioides	CNUCC 196-5-3	*Litsea cubeba*	China	PP843262	PP849762	PP841670	PP922842	PP890402	PP854739
C. gloeosporioides	CNUCC 225A-3- 2	*Camellia oleifera*	China	PP843273	PP849773	PP841681	PP922853	PP890413	PP854750
C. gloeosporioides	CNUCC 884-1-1	*Camellia oleifera*	China	PP843314	PP849814	PP841722	PP922894	PP870984	PP854790

Supplementary Table 4 Continued

Species name	Strain	Host	Country	ITS	gapdh	chs-1	act	tub2	*cal*
C. gloeosporioides	CNUCC 914-2-1	*Dalbergia hupeana*	China	PP843242	PP849742	PP841650	PP922822	PP886091	PP854720
C. gloeosporioides	CNUCC 949-2-2	*Paulownia fortunei*	China	PP843325	PP849825	PP841733	PP922905	PP870995	PP854801
C. gloeosporioides	CNUCC 61-1-1	*Cunninghamia lanceolata*	China	PP843293	PP849793	PP841701	PP922873	PP890431	PP854769
C. gloeosporioides* syn. *C. juglandicola	**CGMCC3.24312 T**	***Juglans regia***	**China**	**OQ263015**	**OQ282973**	**OR004793**	**OQ282966**	**OQ282980**	**/**
C. gloeosporioides* syn. *C. juglandicola	**CGMCC3.24313**	***Juglans regia***	**China**	**OQ263018**	**OQ282977**	**OR004797**	**OQ282970**	**OQ282984**	**/**
C. gloeosporioides* syn. *C. juglandium	**CFCC 59974 T**	***Juglans regia***	**China**	**PP397130**	**PP425082**	**PP425040**	**PP424997**	**/**	**/**
C. gloeosporioides* syn. *C. juglandium	**CFCC 70165**	***Juglans regia***	**China**	**PP397131**	**PP425083**	**PP425041**	**PP424998**	**/**	**/**
C. gloeosporioides* syn. *C. peakense	**CGMCC3.24308 T**	***Juglans regia***	**China**	**OQ263017**	**OQ282975**	**OR004795**	**OQ282968**	**OQ282982**	**/**
C. gloeosporioides* syn. *C. peakense	**CGMCC3.24307**	***Juglans regia***	**China**	**OQ263016**	**OQ282974**	**OR004794**	**OQ282967**	**OQ282981**	**/**
C. gloeosporioides	**CFCC 72580**	***Juglans regia***	**China**	**PV524703**	**PV573493**	**PV573525**	**PV573554**	**PV573585**	**/**
C. gloeosporioides	**CFCC 72581**	***Juglans regia***	**China**	**PV524704**	**PV573494**	**/**	**/**	**PV573586**	**/**
C. gloeosporioides	**CFCC 72440**	***Prunus cerasifera 'Atropurpurea'***	**China**	**PV524705**	**PV573495**	**PV573526**	**PV573555**	**PV573587**	**PV573617**
C. gloeosporioides	**CFCC 72441**	***Prunus cerasifera 'Atropurpurea'***	**China**	**PV524706**	**PV573496**	**PV573527**	**PV573556**	**PV573588**	**PV573618**
C. gloeosporioides	**LFPR 10012**	***Amorpha fruticosa***	**China**	**PV878373**	**PV975946**	**/**	**/**	**PV975912**	**/**
C. gloeosporioides	**LFPR 10013**	***Amorpha fruticosa***	**China**	**PV878374**	**PV975947**	**/**	**/**	**PV975913**	**/**
C. gloeosporioides	**LFPR 10014**	***Juglans regia***	**China**	**PV878375**	**PV975948**	**/**	**/**	**PV975914**	**/**
C. gloeosporioides	**LFPR 10015**	***Quercus aliena***	**China**	**PV878376**	**PV975949**	**/**	**/**	**PV975915**	**/**
C. gloeosporioides	**LFPR 10016**	***Parthenocissus quinquefolia***	**China**	**PV878377**	**PV975950**	**/**	**/**	**PV975916**	**/**
C. gloeosporioides	**LFPR 10017**	***Parthenocissus tricuspidata***	**China**	**PV878378**	**PV975951**	**/**	**/**	**PV975917**	**/**
C. gloeosporioides	**LFPR 10018**	***Microlepia marginata***	**China**	**PV878379**	**PV975952**	**/**	**/**	**PV975918**	**/**

Supplementary Table 4 Continued

Species name	Strain	Host	Country	ITS	*gapdh*	*chs-1*	*act*	*tub2*	*cal*
C. gloeosporioides	**LFPR 10019**	***Robinia pseudoacacia***	**China**	**PV878380**	**PV975953**	/	/	**PV975919**	/
C. gloeosporioides	**LFPR 10020**	***Fraxinus chinensis***	**China**	**PV878381**	**PV975954**	/	/	**PV975920**	/
C. gloeosporioides	**LFPR 10021**	***Amorpha fruticosa***	**China**	**PV878382**	**PV975955**	/	/	**PV975921**	/
C. gloeosporioides	**LFPR 10022**	***Prunus persica 'Duplex'***	**China**	**PV878383**	**PV975956**	/	/	**PV975922**	/
C. gloeosporioides	**LFPR 10023**	***Malus spectabilis***	**China**	**PV878384**	**PV975957**	/	/	**PV975923**	/
C. gloeosporioides	**LFPR 10024**	***Kerria japonica***	**China**	**PV878385**	**PV975958**	/	/	**PV975924**	/
C. gloeosporioides	**LFPR 10025**	***Juglans regia***	**China**	**PV878386**	**PV975959**	/	/	**PV975925**	/
C. grevilleae	CBS 132879 = CPC 15481 T	*Grevillea* sp.	Italy	KC297078	KC297010	KC296987	KC296941	KC297102	KC296963
C. grossum	CGMCC 3.17614 = CAUG 7 = LC 6227 T	Chili pepper	China	KP890165	KP890159	KP890153	KP890141	KP890171	KP890147
C. guangyuanense	CFCC 59902 T	*Juglans regia*	China	PP397133	PP425084	PP425043	PP425000	PP425125	/
C. guangyuanense	CFCC 70249	*Juglans regia*	China	PP397134	PP425085	PP425044	PP425001	PP425126	/
C. guiyangense	CNUCC 823-1-1-1 T	*Camellia sinensis*	China	PP812207	PP819551	PP819547	PP830762	PP830770	PP830766
C. guiyangense	CNUCC 823-1-1-2	*Camellia sinensis*	China	PP812208	PP819552	PP819548	PP830763	PP830771	PP830767
C. hebeiense	MFLUCC 130726 T	*Vitis vinifera*	China	KF156863	KF377495	KF289008	KF377532	KF288975	/
C. hedericola	MFLU 15-0689 T	*Hedera helix*	Italy	MN631384	/	MN635794	MN635795	/	/
C. helleniense	CBS 142418 = CPC 26844 T	*Poncirus trifoliata*	Greece, Arta	KY856446	KY856270	KY856186	KY856019	KY856528	KY856099
C. henanense	LC 3030 = CGMCC 3.17354 = LF 238 T	*Camellia sinensis*	China	KJ955109	KJ954810	MZ799256	KM023257	KJ955257	KJ954662
C. horii	ICMP 10492 = NBRC 7478 T	*Diospyros kaki*	Japan	GQ329690	GQ329681	JX009752	JX009438	JX010450	JX009604
C. hystricis	CBS 142411 = CPC 28153 T	*Citrus hystrix*	Italy, Catania	KY856450	KY856274	KY856190	KY856023	KY856532	KY856103
C. jiangxiense	LC 3463 = CGMCC 3.17363 = LF 687 T	*Camellia sinensis*	China	KJ955201	KJ954902	/	KJ954471	KJ955348	KJ954752
C. jiangxiense	LC 3266 = CGMCC 3.17361 = LF 488	*Camellia sinensis*	China	KJ955149	KJ954850	MZ799257	KJ954427	OK236389	KJ954701
C. jinpingense	CCTCC AF2021056 T	*Hevea brasiliensis*	China	MZ165534	MZ361085	MZ352021	MZ277632	MZ277688	/

Supplementary Table 4 Continued

Species name	Strain	Host	Country	ITS	gapdh	chs-1	act	tub2	*cal*
C. jixiense	CNUCC 5-21-2-1 T	*Cunninghamia lanceolata*	China	PP812209	PP819553	PP819549	PP830764	PP830772	PP830768
C. kahawae	IMI 319418 = ICMP 17816 T	*Coffea arabica*	Kenya	JX010231	JX010012	JX009813	JX009452	JX010444	JX009642
C. kunmingense	GUCC 12053 T	*Ophiopogon japonicus*	China	OP722975	OP737965	OP715769	OP715804	OP720861	/
C. kunmingense	GUCC 12178	*Ophiopogon japonicus*	China	OP722974	OP784058	OP715770	OP715805	/	/
C. ledongense	CGMCC 3.18888 T	*Hevea brasiliensis*	China	MG242009	MG242017	MG242019	MG242015	MG242011	MG242013
C. ligustri	GUCC 12111 T	*Ilex chinensis*	China	OP722988	OP737968	OP715773	OP740216	OP720864	/
C. lumnitzerae	MFLUCC 23-0291	*mangroves*	Thailand	OR856122	OR886391	OR886403	OR886394	OR886397	/
C. makassarense	CBS 143664 T	*Capsicum annuum*	Indone- sia	MH728812	MH728820	MH805850	MH781480	MH846563	/
C. musae	CBS 116870 = ICMP 19119	*Musa* sp.	USA	HQ596292	HQ596299	JX009896	HQ596284	HQ596280	JX009742
C. nanjingense	CFCC 58939	*J. mesnyi*	China	OQ456154	OQ507143	OQ507140	OQ507150	OQ507146	/
C. nanjingense	CFCC 58940 T	*J. mesnyi*	China	OQ456155	OQ507144	OQ507141	OQ507149	OQ507147	/
C. nullisetosum	YMF 1.06946 T	*Mangifera indica*	China	OK030872	OK513675	OK513571	OK513611	OK513641	/
C. nupharicola	CBS 470.96 = ICMP 18187 T	*Nuphar lutea* subsp. *polysepala*	USA	JX010187	JX009972	JX009835	JX009437	JX010398	JX009663
C. oblongisporum	YMF 1.06938 T	*Ageratina adenophora*	China	OK030874	OK513677	OK513573	/	OK513643	/
C. osmanthicola	AG 36 T	*Osmanthus fragrans*	China	OL772733	OL854168	OL854154	OM100914	OL854182	OL854143
C. perseae	CBS 141365 = GA100 T	Avocado	Israel	KX620308	KX620242	MZ799260	KX620145	KX620341	KX620206
C. perseae	GA 177	Avocado	Israel	KX620311	KX620245	/	KX620148	KX620344	KX620209
C. perseae	GA 272 = CBS 141366	Avocado	Israel	KX620321	KX620255	/	KX620158	KX620354	KX620220
C. polypodialium	MFLUCC 22-0178	*Nephrolepis* sp.	Thailand	OP802361	OP801720	OP801702	OP801685	OP801739	/
C. proteae	CBS 132882 = CPC 14859 T	*Protea* sp.	South Africa	KC297079	KC297009	KC296986	KC296940	KC297101	KC296960
C. pseudotheobromicola	MFLUCC 181602 T	*Prunus avium*	China	MH817395	MH853675	MH853678	MH853681	MH853684	/
C. psidii	CBS 145.29 = ICMP 19120	*Psidium* sp.	Italy	JX010219	JX009967	JX009901	JX009515	JX010443	JX009743

Supplementary Table 4 Continued

Species name	Strain	Host	Country	ITS	gapdh	chs-1	act	tub2	*cal*
C. queenslandicum	ICMP 1778	*Carica papaya*	Australia	JX010276	JX009934	JX009899	JX009447	JX010414	JX009691
C. rhexiae	Coll 1026 = BPI 884112 = CBS 133134	*Rhexia virginica*	USA	JX145128	MZ664046	MZ799258	MZ664127	JX145179	/
C. salsolae	CBS 119296 = ICMP 18693	*Glycine max*	Hungary	JX010241	JX009917	JX009791	JX009559	/	JX009695
C. saudianum	PPDU38H T	*Coffea arabica*	Saudi Arabia	OR048759	/	OR050722	OR050670	OR050767	/
C. saudianum	PPDU38F	*Coffea arabica*	Saudi Arabia	OR048760	/	OR050723	OR050671	OR050768	/
C. saudianum	PPDU29B	*Coffea arabica*	Saudi Arabia	OR048769	OR050750	OR050732	OR050680	OR050777	/
C. saudianum	PPDU29A	*Coffea arabica*	Saudi Arabia	OR048770	OR050751	OR050733	OR050681	OR050778	/
C. siamense	ICMP 18578 = CBS 130417 T	*Coffea arabica*	Thailand	JX010171	JX009924	JX009865	FJ907423	JX010404	/
C. siamense	LC 0148	*Camellia* sp.	China	KJ955078	KJ954779	/	KJ954360	KJ955227	KJ954631
C. siamense	LC 0149	*Camellia* sp.	China	KJ955079	KJ954780	/	KJ954361	KJ955228	KJ954632
C. siamense	CNUCC 180-1-3	*Lindera glauca*	China	PP843256	PP849756	PP841664	PP922836	PP886105	PP854733
C. siamense	CNUCC 191-3-1	*Partheno-cissus*	China	PP843259	PP849759	PP841667	PP922839	/	PP854736
C. siamense	CNUCC 196-4-1	*Litsea cubeba*	China	PP843261	PP849760	PP841669	PP922912	PP886109	PP854738
C. siamense	CNUCC 211-5-2	*Vernicia fordii*	China	PP843263	PP849763	PP841671	PP922843	PP890403	PP854740
C. siamense	CNUCC 236-4-4	*Machilus nanmu*	China	PP843282	PP849782	PP841690	PP922862	PP890422	PP854759
C. siamense	CNUCC 271-5-1	*Firmiana simplex*	China	PP843286	PP849786	PP841694	PP922866	PP890426	PP854763
C. siamense	CNUCC 284-1-1	*Lauraceae* sp.	China	PP843287	PP849787	PP841695	PP922867	/	PP854764
C. siamense	CNUCC 540-1-3	*Euonymus alatus*	China	PP843291	PP849791	PP841699	PP922871	/	PP854768
C. siamense	CNUCC 61-20-3	*Cunninghamia lanceolata*	China	PP843328	PP849828	PP841736	PP922908	PP870998	PP854804
C. siamense	CNUCC 61-25-1	*Cunninghamia lanceolata*	China	PP843329	PP849829	PP841737	PP922909	PP870999	PP854805
C. siamense	**CFCC 72430**	***Chamaedorea pinnatifrons***	**China**	**PV524717**	**PV573507**	**PV573538**	**PV573567**	**PV573599**	**PV573629**
C. siamense	**CFCC 72431**	***Chamaedorea pinnatifrons***	**China**	**PV524718**	**PV573508**	**PV573539**	**PV573568**	**PV573600**	**PV573630**
C. siamense	**CFCC 72605**	***Euonymus japonicus***	**China**	**PV524719**	**PV573509**	**PV573540**	**PV573569**	**PV573601**	**/**

Supplementary Table 4 Continued

Species name	Strain	Host	Country	ITS	gapdh	chs-1	act	tub2	*cal*
C. siamense	**CFCC 72606**	***Euonymus japonicus***	**China**	**PV524720**	**PV573510**	**PV573541**	**PV573570**	**PV573602**	**/**
C. siamense	**CFCC 72432**	***Lagerstroemia speciosa***	**China**	**PV524709**	**PV573499**	**PV573530**	**PV573559**	**PV573591**	**PV573621**
C. siamense	**CFCC 72433**	***Lagerstroemia speciosa***	**China**	**PV524710**	**PV573500**	**PV573531**	**PV573560**	**PV573592**	**PV573622**
C. siamense	**LFPR 10010**	***Broussonetia papyrifera***	**China**	**PV878371**	**PV975944**	**/**	**/**	**PV975910**	**/**
C. siamense	**LFPR 10011**	***Nandina domestica***	**China**	**PV878372**	**PV975945**	**/**	**/**	**PV975911**	**/**
C. subhenanense	YMF 1.07324	*camphora Ageratina adenophora*	China	OK030884	OK513685	OK513582	OK513619	OK513648	/
C. subhenanense	YMF 1.06865	*Ageratina adenophora*	China	OK030883	OK513684	OK513581	OK513618	OK513647	/
C. syzygicola	DNCL 021 = MFLUCC 10-0624	*Syzygium samarangense*	Thailand	KF242094	KF242156	/	KF157801	KF254880	KF254859
C. tainanense	CBS 143666	*Capsicum annuum*	Taiwan	MH728818	MH728823	MH805845	MH781475	MH846558	/
C. temperatum	CBS 133122 = Coll883 = BPI 884100	*Vaccinium macrocarpon*	USA	JX145159	MZ664045	MZ799254	MZ664125	JX145211	/
C. tengchongense	YMF 1.04950	*Isoetes sinensis*	China	OL842169	OL981264	OL981290	PP498771	PP498785	PP498784
C. theobromicola	ICMP 18649 = CBS 124945	*Theobroma cacao*	Panama	JX010294	JX010006	JX009869	JX009444	JX010447	JX009591
C. ti	ICMP 4832	*Cordyline sp.*	New Zealand	JX010269	JX009952	JX009898	JX009520	JX010442	JX009649
C. tropicale	CBS 124949 = ICMP 18653	*Theobroma cacao*	Panama	JX010264	JX010007	JX009870	JX009489	JX010407	JX009719
C. vulgaris	CGMCC 3.18940 = YMF 1.04940 T	*Hippuris vulgaris*	China	OL842170	OL981265	OL981291	OL981239	/	/
C. wuxiense	CGMCC 3.17894 = JS1A32 T	*Camellia sinensis*	China	KU251591	KU252045	KU251939	KU251672	KU252200	KU251833
C. wuxiense	JS1A44	*Camellia sinensis*	China	KU251592	KU252046	KU251940	KU251673	KU252201	KU251834
C. xanthorrhoeae	BRIP 45094 = ICMP 17903 = CBS 127831 T	*Xanthorrhoea preissii*	Australia	JX010261	JX009927	JX009823	JX009478	JX010448	JX009653
C. xishuangbannaense	MFLUCC 190107 T	*Magnolia liliifera*	China	MW346469	MW537586	MW660832	MW652294	/	/
C. yunajiangensis	YMF 1.04996 = CGMCC 3.18964 T	*Ageratina adenophora*	China	OK030885	OK513686	OK513583	OK513620	OK513649	/
C. zingibericola	LM937	*Etlingera elatior*	Brazil	/	MZ264106	/	/	MZ270522	/
C. zingibericola	LM942 T	*Etlingera elatior*	Brazil	/	MZ264104	/	/	MZ270520	/

Supplementary Table 5 GenBank accession numbers of the sequences used in the phylogenetic analyses of *C. orchidearum* species complex in this study.

Species name	Strains1	Host	Country	ITS	*gapdh*	*chs-1*	*act*	*tub2*	*his3*
C. cattleyicola	CBS 170.49 T	*Cattleya sp.*	Belgium	MG600758	MG600819	MG600866	MG600963	MG601025	MG600905
C. cattleyicola	MAFF 238321	*Cattleya sp.*	Japan	MG600759	/	/	/	MG601026	/
C. cliviicola	CBS 125375 T	*Clivia miniata*	China	MG600733	MG600795	MG600850	MG600939	MG601000	MG600892
C. cliviicola	CBS 133705	*Clivia* sp.	South Africa	MG600732	MG600794	MG600849	MG600938	MG600999	MG600891
C. monsterae	NN055214 T	*Monstera deliciosa*	China	MZ595897	MZ664121	MZ799351	MZ664195	MZ674015	MZ673917
C. musicola	CBS 132885 T	*Musa* sp.	Mexico	MG600736	MG600798	MG600853	MG600942	MG601003	MG600895
C. musicola	CBS 127557	*Musa* sp.	Mexico	MG600737	MG600799	MG600854	MG600943	MG601004	MG600896
C. orchidearum	CBS 136877	*Dendrobium nobile*	Netherlands	MG600739	MG600801	MG600856	MG600945	MG601006	MG600898
C. orchidearum	CBS 135131 T	*Dendrobium nobile*	Netherlands	MG600738	MG600800	MG600855	MG600944	MG601005	MG600897
C. orchidearum	**LFPR 10029**	***Anthurium andraeanum***	**China**	**PV878390**	**PV975962**	/	/	**PV975929**	/
C. orchidearum	**LFPR 10030**	***Anthurium andraeanum***	**China**	**PV878391**	**PV975963**	/	/	**PV975930**	/
C. pereskiae	COAD 2995 T	*Pereskia*	Brazil	MZ262421	MZ265337	MZ265335	MZ265333	MZ265341	MZ265339
C. piperis	IMI 71397 T	*Piper nigrum*	Malaysia	MG600760	MG600820	MG600867	MG600964	MG601027	MG600906
C. plurivorum	LC8322	*Paederia foetida*	China	MZ595853	MZ664114	MZ799293	MZ664151	MZ673974	MZ673873
C. plurivorum	LC8244	*Paederia foetida*	China	MZ595849	MZ772868	MZ799292	MZ664147	MZ673970	MZ673869
C. plurivorum	LC8240	*Paederia foetida*	China	MZ595848	MZ664113	MZ799291	MZ664146	MZ673969	MZ673868
C. plurivorum	LC8337	*Vigna unguiculata*	Vigna unguiculata	MZ595855	MZ664115	MZ799294	MZ664153	MZ673976	MZ673875
C. plurivorum	CBS 132443	*Gossypium* sp.	Brazil	MG600719	MG600782	MG600842	MG600926	MG600986	MG600888
C. plurivorum	CBS 125474 T	*Coffea* sp.	Vietnam	MG600718	MG600781	MG600841	MG600925	MG600985	MG600887
C. plurivorum	**LFPR 10026**	***Nandina domestica***	**China**	**PV878387**	**PV975960**	/	/	**PV975926**	/
C. plurivorum	**LFPR 10027**	***Spathiphyllum***	**China**	**PV878388**	/	/	/	**PV975927**	/

Supplementary Table 5 Continued

Species name	Strains1	Host	Country	ITS	*gapdh*	*chs-1*	*act*	*tub2*	*his3*
C. plurivorum	**LFPR 10028**	***Megaskepasma erythrochlamys***	**China**	**PV878389**	**PV975961**	/	/	**PV975928**	/
C. plurivorum* syn. *C. subplurivorum	**CNUCC 184-5-5**	***Diospyros kaki var. silvestris***	**China**	**PP840578**	**PP841300**	**PP841285**	**PP841280**	**PP841295**	**PP841290**
C. plurivorum* syn. *C. subplurivorum	**CNUCC 175C-1-3**	***Camellia sinensis***	**China**	**PP840579**	**PP841301**	**PP841286**	**PP841281**	**PP841296**	**PP841291**
C. plurivorum* syn. *C. subplurivorum	**CNUCC 811A-1-1**	***Camellia sinensis***	**China**	**PP840580**	**PP841302**	**PP841287**	**PP841282**	**PP841297**	**PP841292**
C. plurivorum* syn. *C. subplurivorum	**CNUCC 824-1-2**	***Camellia sinensis***	**China**	**PP840581**	**PP841303**	**PP841288**	**PP841283**	**PP841298**	**PP841293**
C. plurivorum* syn. *C. subplurivorum	**CNUCC 833B-1-1**	***Camellia oleifera***	**China**	**PP840582**	**PP841304**	**PP841289**	**PP841284**	**PP841299**	**PP841294**
C. reniforme	LC8230 T	*Smilax cocculoides*	China	MZ595847	MZ664110	MZ799290	MZ664145	MZ673968	MZ673867
C. reniforme	LC8248	*Paederia foetida*	China	MZ595850	MZ664111	MZ799295	MZ664148	MZ673971	MZ673870
C. sojae	CBS 128510	*Medicago sativa*	USA	MG600751	MG600812	MG600862	MG600956	MG601018	MG600901
C. sojae	ATCC 62257 T	*Glycine max*	USA	MG600749	MG600810	MG600860	MG600954	MG601016	MG600899
C. sojae	M316	*Phaseolus vulgaris*	China	MZ595858	MZ664116	MZ799301	MZ664156	MZ673979	MZ673878
C. sojae	M149	*Patrinia villosa*	China	MZ595854	MZ664112	MZ799300	MZ664152	MZ673975	MZ673874
C. sojae	**LFPR 10031**	***Lonicera maackii***	**China**	**PV878392**	**PV975964**	/	/	**PV975931**	/
C. sojae	**LFPR 10032**	***Lonicera maackii***	**China**	**PV878393**	**PV975965**	/	/	**PV975932**	/
C. syngoniicola	LC8895	*Syngonium* sp.	China	MZ595864	MZ664118	MZ799297	MZ664162	MZ673983	MZ673884
C. syngoniicola	LC8894 T	*Syngonium* sp.	China	MZ595863	MZ664117	MZ799296	MZ664161	MZ673982	MZ673883
C. syngoniicola	LC8896	*Syngonium* sp.	China	MZ595865	MZ664119	MZ799298	MZ664163	MZ673984	MZ673885
C. syngoniicola	LC8897	*Syngonium* sp.	China	MZ595866	MZ664120	MZ799299	MZ664164	MZ673985	MZ673886
C. vittalense	CBS 181.82 T	*Theobroma cacao*	India	MG600734	MG600796	MG600851	MG600940	MG601001	MG600893
C. vittalense	CBS 126.25	*Orchid*	unknown	MG600735	MG600797	MG600852	MG600941	MG601002	MG600894

Supplementary Table 6 GenBank accession numbers of the sequences used in the phylogenetic analyses of *C. spaethianum* species complex in this study.

Species name	Strains1	Host	Country	ITS	*gapdh*	*chs-1*	*act*	*tub2*	*his3*
C. bicoloratum	NN055229 T	*Ophiopogon japonicus*	China	MZ595899	MZ664100	MZ799332	MZ673919	MZ664197	MZ674017
C. bletillae	CGMCC 3.15117 T	*Bletilla ochracea*	China	JX625178	KC843506	MZ799322	MZ673854	KC843542	JX625207
C. guizhouensis	CGMCC 3.15113	*Bletilla ochracea*	China	JX625164	KC843508	/	/	KC843537	JX625192
C. guizhouensis	CGMCC 3.15112 T	*Bletilla ochracea*	China	JX625158	KC843507	MZ799321	MZ673850	KC843536	JX625185
C. incanum	ATCC 64682 T	*Phaseolus vulgaris*	Canada	KC110789	KC110807	/	KC110798	KC110825	KC110816
C. incanum	CBS 133485	*Glycine max*	USA	KC110787	KC110805	/	KC110796	KC110823	KC110814
C. iris	LC3697	*Iris japonica*	China	MZ595837	MZ664090	MZ799323	MZ673856	MZ664135	MZ673958
C. lilii	CBS 109214	*Lilium sp.*	Japan	GU227810	GU228202	GU228300	GU228006	GU227908	GU228104
C. liriopes	CBS 119444 T	*Liriope muscari*	Mexico	GU227804	GU228196	GU228294	GU228000	GU227902	GU228098
C. liriopes	CBS 122747	*Liriope muscari*	Mexico	GU227805	GU228197	GU228295	GU228001	GU227903	GU228099
C. liriopes	NN071073	*Osmanthus fragrans*, dead leaves	China	MZ595908	MZ664093	MZ799326	MZ673928	MZ664206	MZ674026
C. liriopes	LC11287	*Liriope spicata*	China	MZ595843	MZ664092	MZ799325	MZ673863	MZ664141	MZ673964
C. liriopes	LC7623	*Poaceae*	China	MZ595842	MZ664091	MZ799324	MZ673862	MZ664140	MZ673963
C. riograndense	COAD 928 T	*Tradescantia fluminensis*	Brazil	KM655299	KM655298	KM655297	/	KM655295	KM655300
C. spaethianum	CBS 167.49 T	*Funkia sieboldiana*, dead stem	Germany	GU227807	GU228199	GU228297	GU228003	GU227905	GU228101
C. spaethianum	**LFPR 10033**	***Hosta plantaginea***	**China**	**PV878394**	**PV975966**	/	/	/	/
C. spaethianum	**LFPR 10034**	***Malus pumila***	**China**	**PV878395**	**PV975967**	/	/	**PV975933**	/
C. spaethianum	**LFPR 10035**	***Malus pumila***	**China**	**PV878396**	**PV975968**	/	/	**PV975934**	/
C. tofieldiae	CBS 495.85	*Tofieldia calyculata*	Switzerland	GU227801	GU228193	GU228291	GU227997	GU227899	GU228095
C. tofieldiae	CBS 168.49	*Lupinus polyphyllus*	Germany	GU227802	GU228194	GU228292	GU227998	GU227900	GU228096
C. verruculosum	IMI 45525 T	*Crotalaria juncea*	Zimbabwe	GU227806	GU228198	GU228296	GU228002	GU227904	GU228100
