# Supplementary figures and images for: Diversity and Distribution of Colletotrichum Species Causing Anthracnose in China
Source: J Fungi (Basel). 2025 Oct 30;11(11):781. doi: 10.3390/jof11110781 (PMC12653916; doi:10.3390/jof11110781)

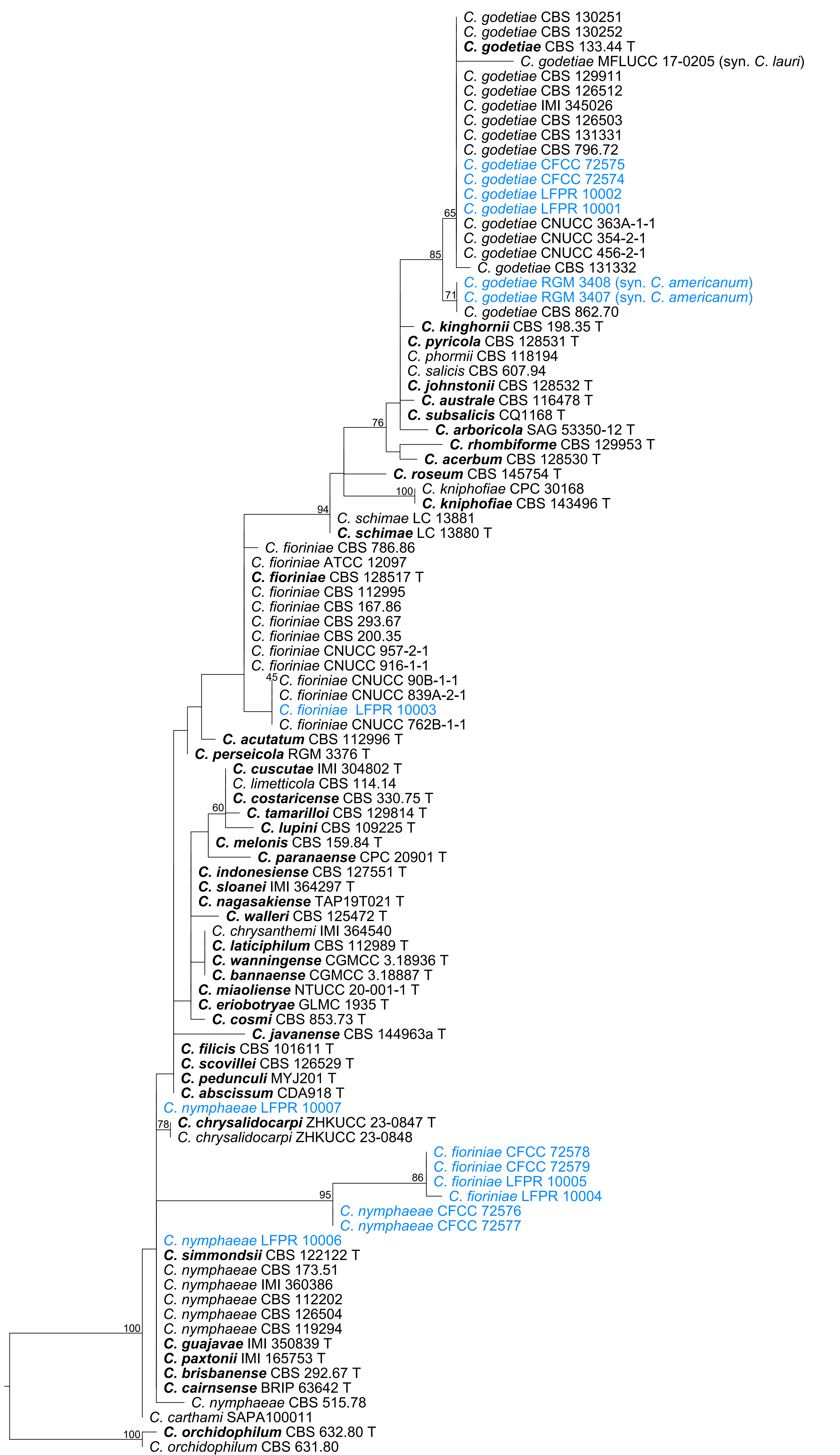

Supplement: Supplementary file 1 [file jof-11-00781-s001.zip › Figure S1.pdf]

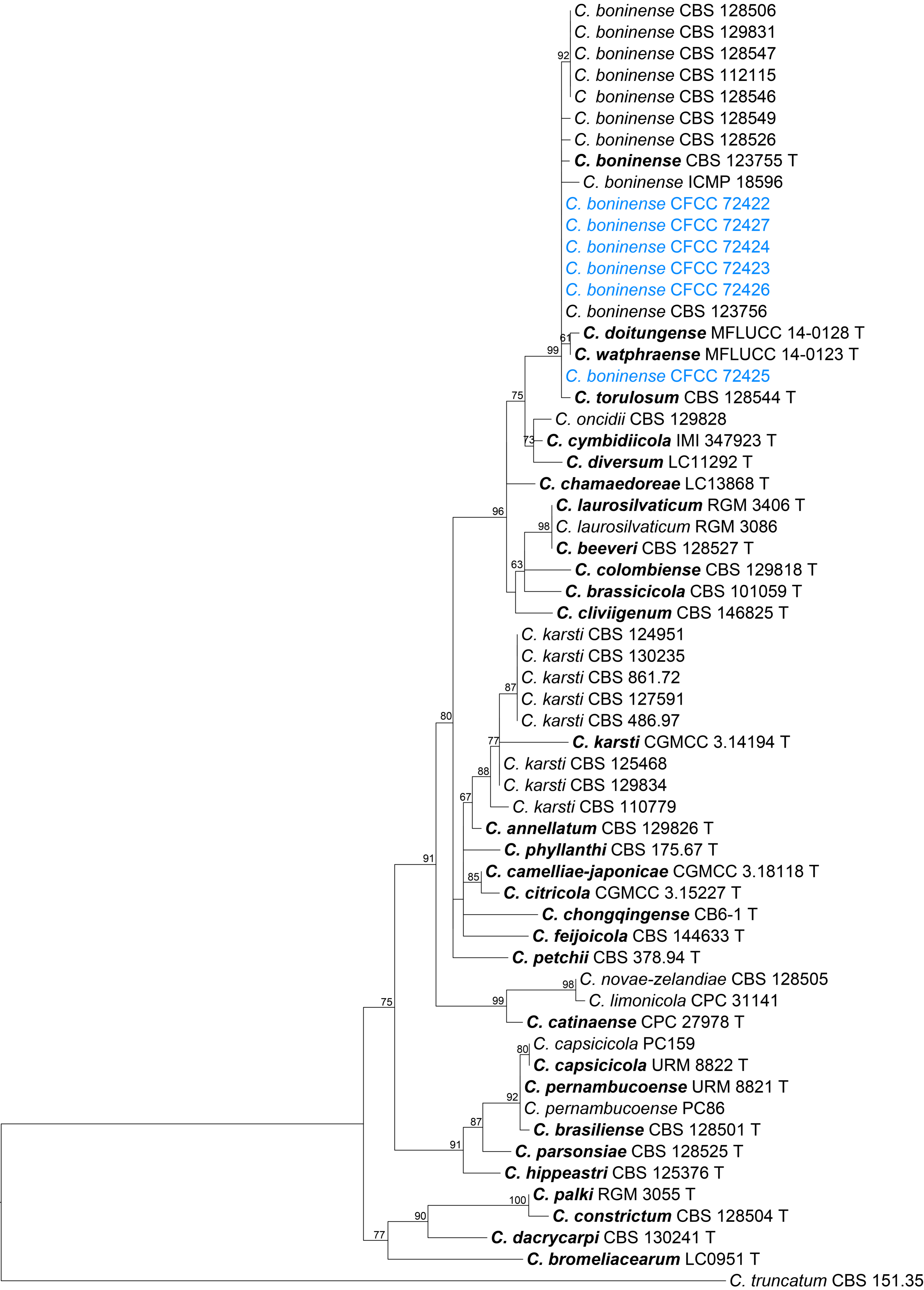

Supplement: Supplementary file 1 [file jof-11-00781-s001.zip › Figure S10.PDF]

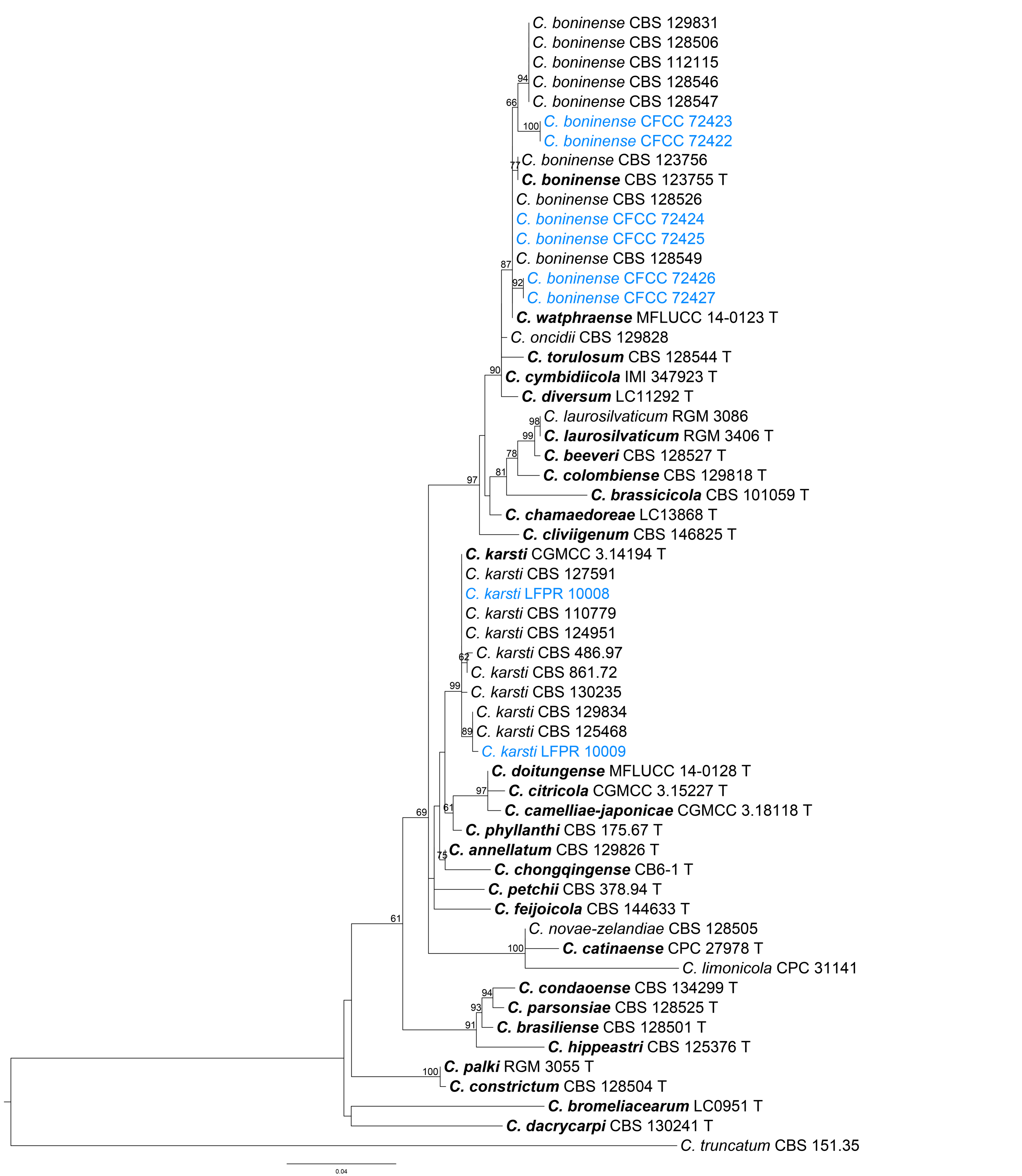

Supplement: Supplementary file 1 [file jof-11-00781-s001.zip › Figure S11.PDF]

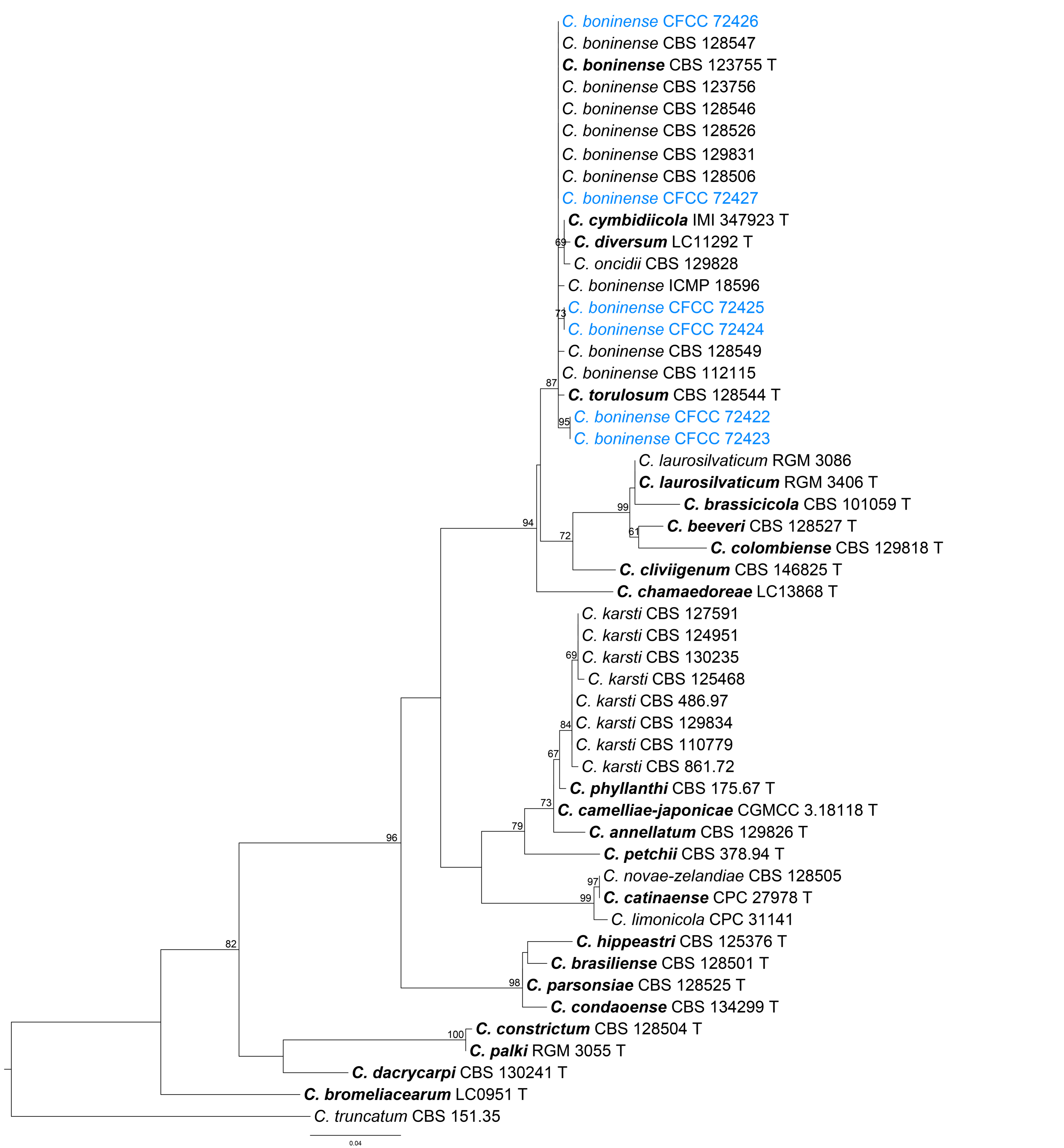

Supplement: Supplementary file 1 [file jof-11-00781-s001.zip › Figure S12.PDF]

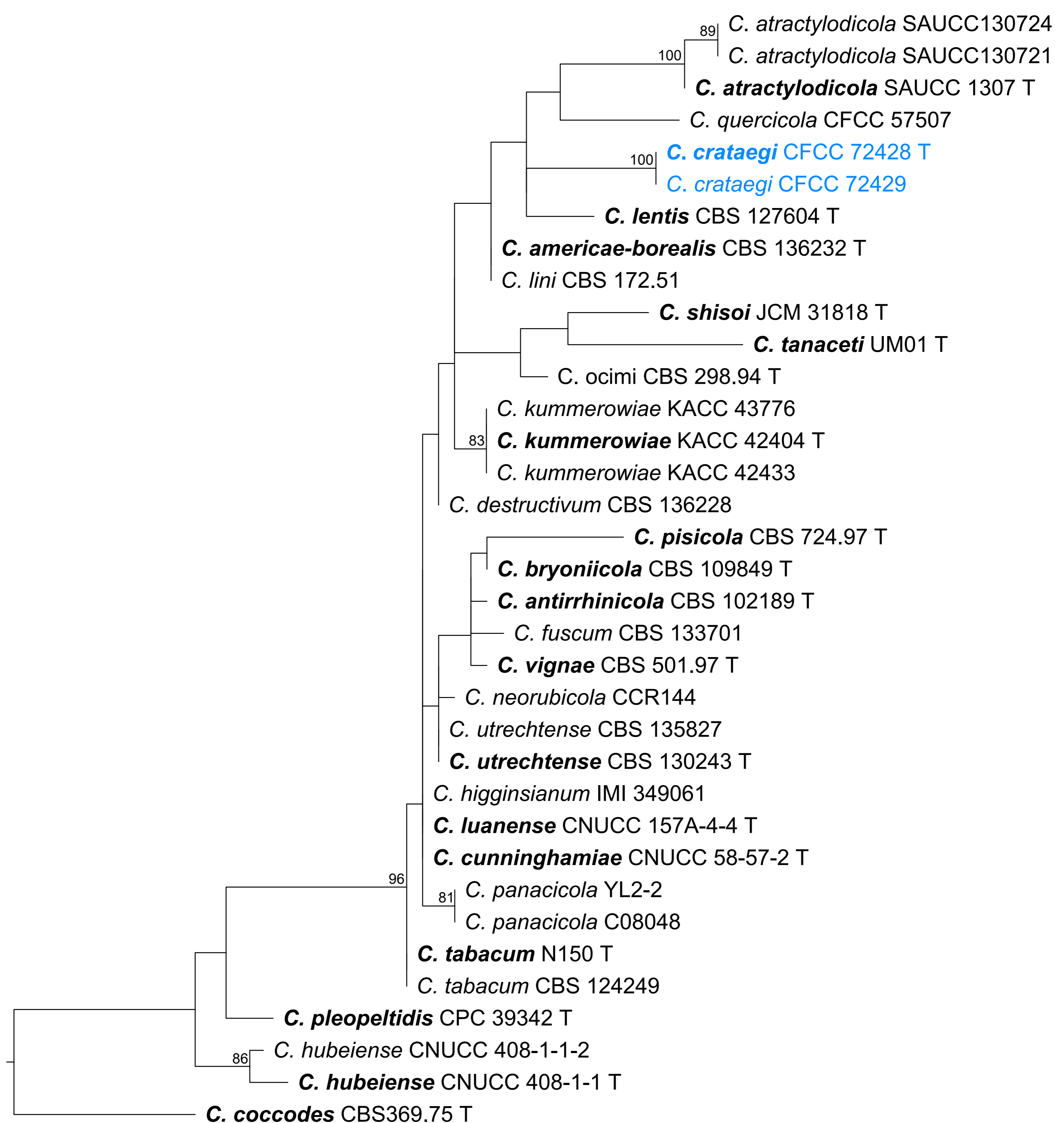

Supplement: Supplementary file 1 [file jof-11-00781-s001.zip › Figure S13.PDF]

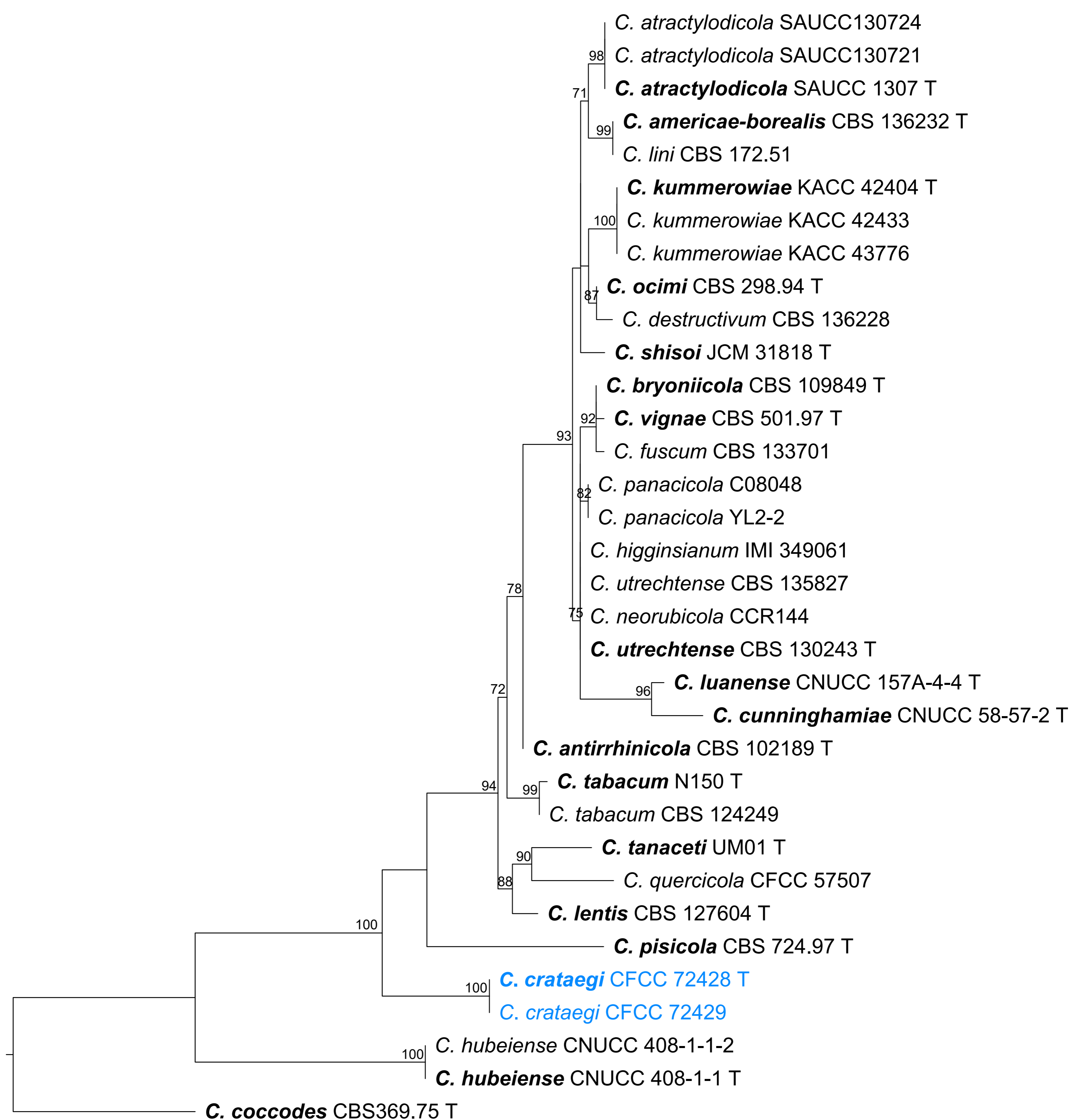

Supplement: Supplementary file 1 [file jof-11-00781-s001.zip › Figure S14.PDF]

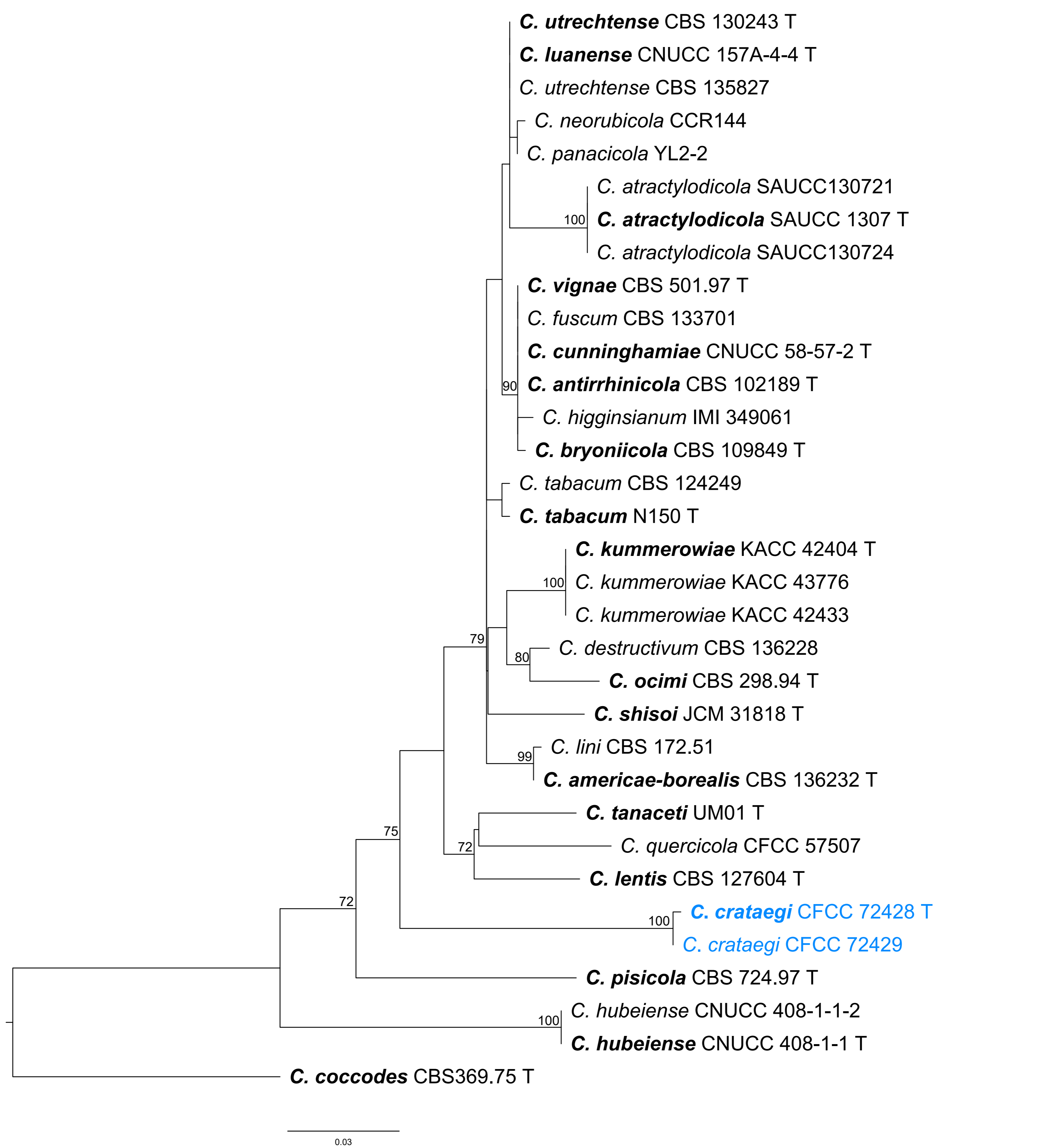

Supplement: Supplementary file 1 [file jof-11-00781-s001.zip › Figure S15.PDF]

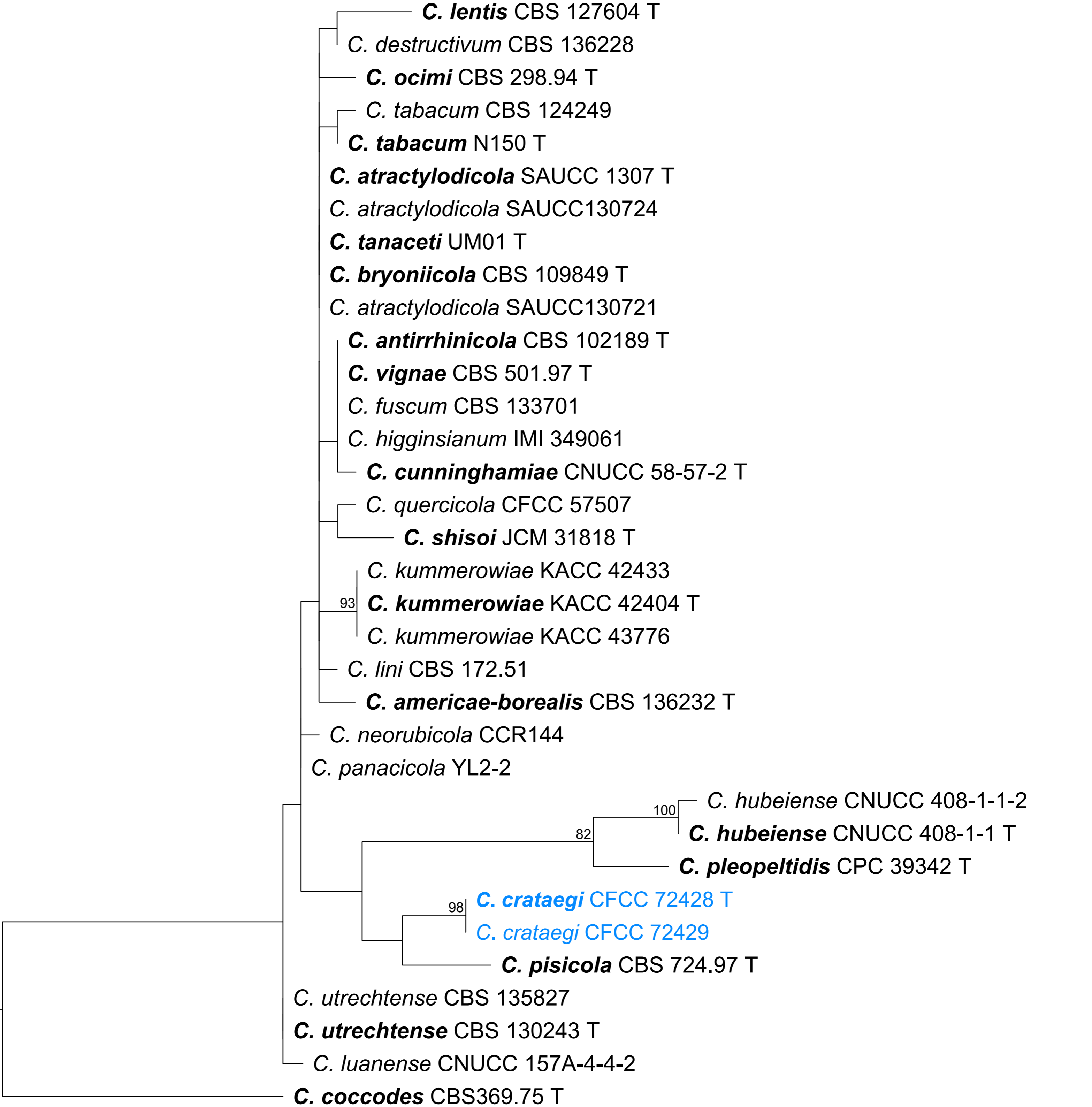

Supplement: Supplementary file 1 [file jof-11-00781-s001.zip › Figure S16.PDF]

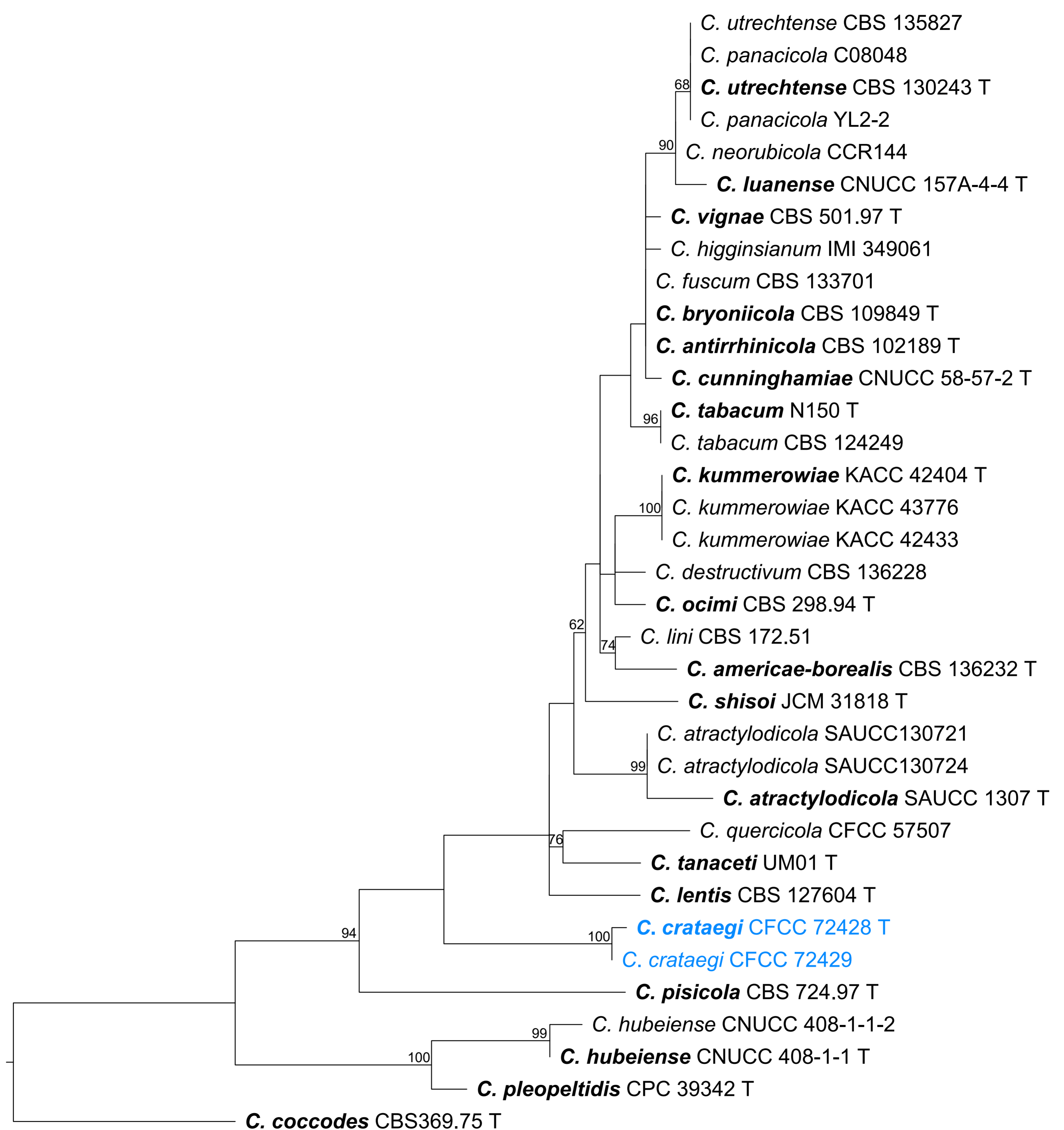

Supplement: Supplementary file 1 [file jof-11-00781-s001.zip › Figure S17.PDF]

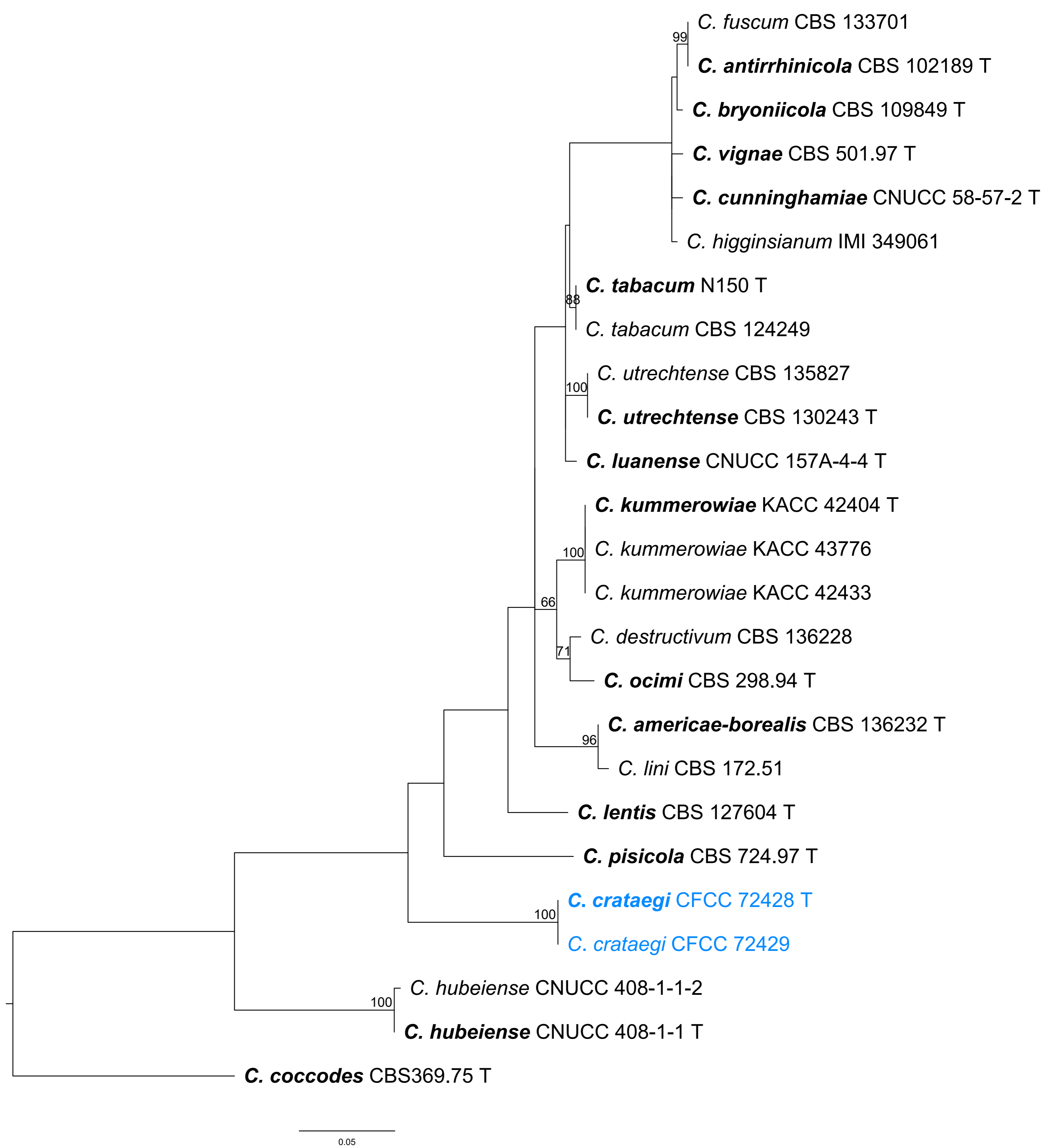

Supplement: Supplementary file 1 [file jof-11-00781-s001.zip › Figure S18.PDF]

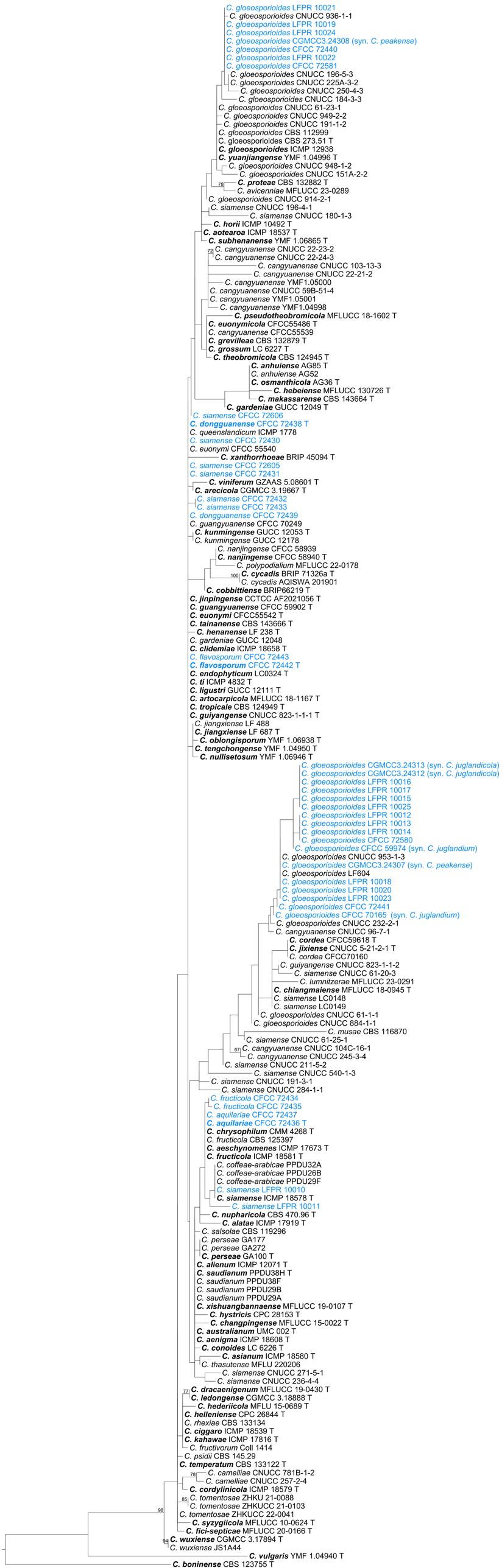

Supplement: Supplementary file 1 [file jof-11-00781-s001.zip › Figure S19.PDF]

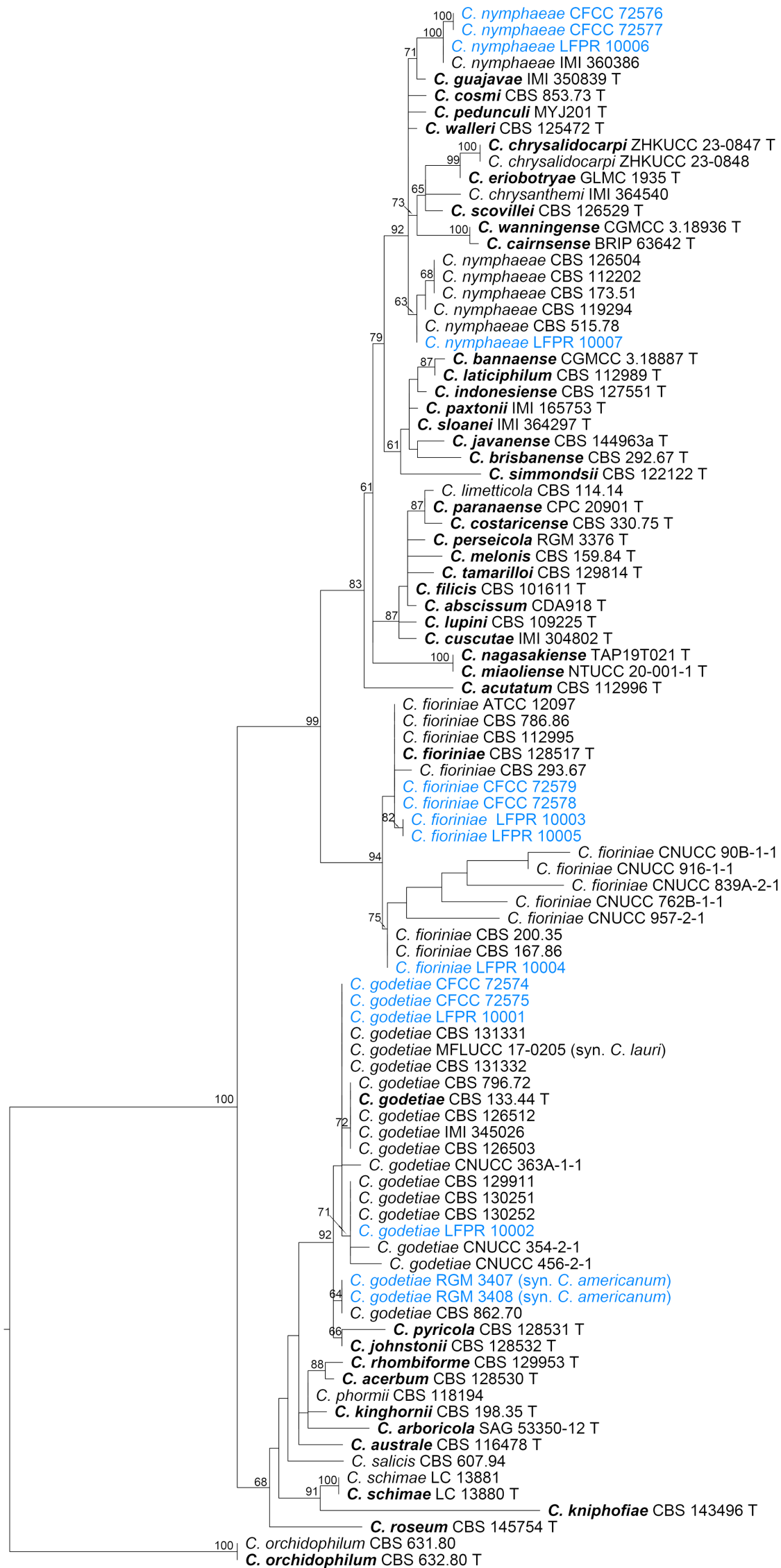

Supplement: Supplementary file 1 [file jof-11-00781-s001.zip › Figure S2.pdf]

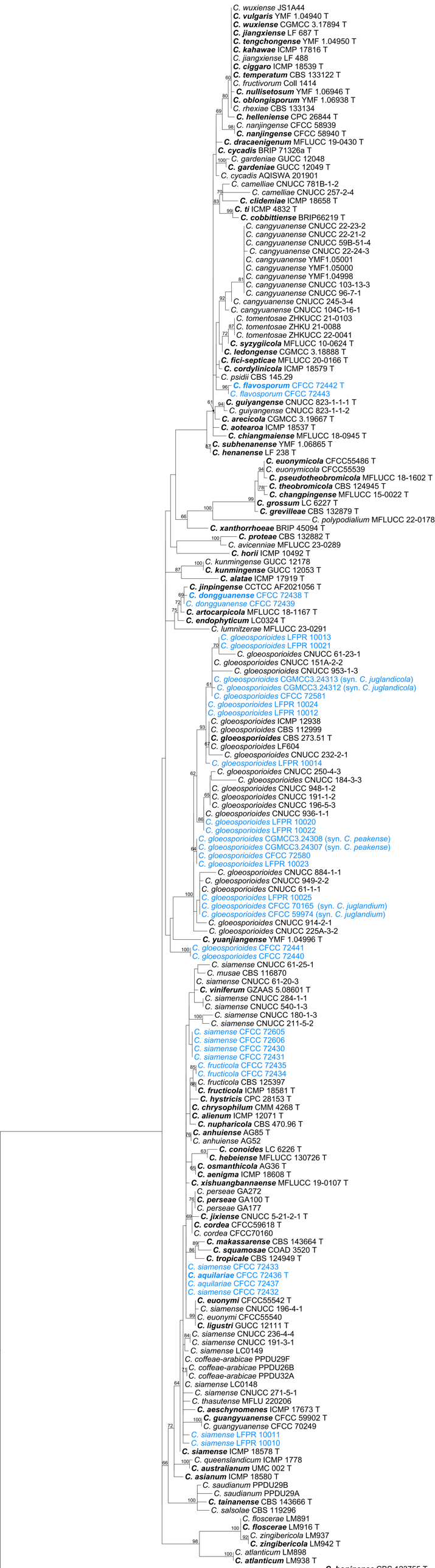

Supplement: Supplementary file 1 [file jof-11-00781-s001.zip › Figure S20.PDF]

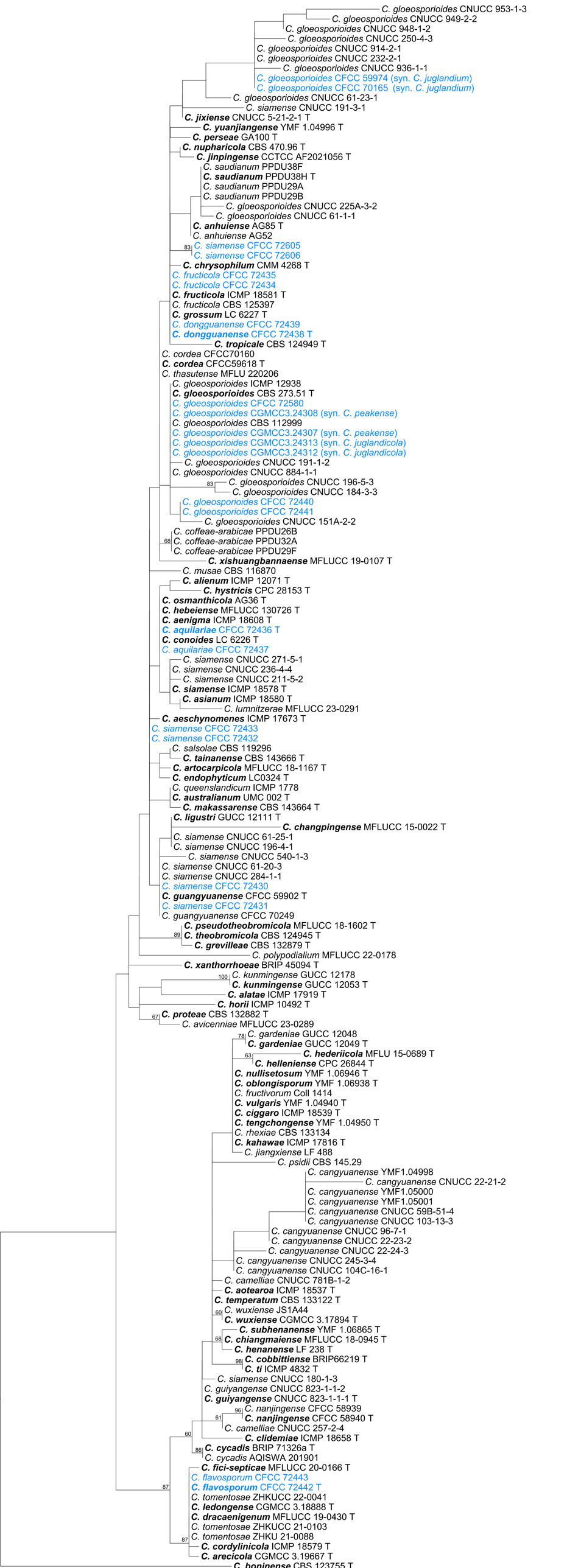

Supplement: Supplementary file 1 [file jof-11-00781-s001.zip › Figure S21.PDF]

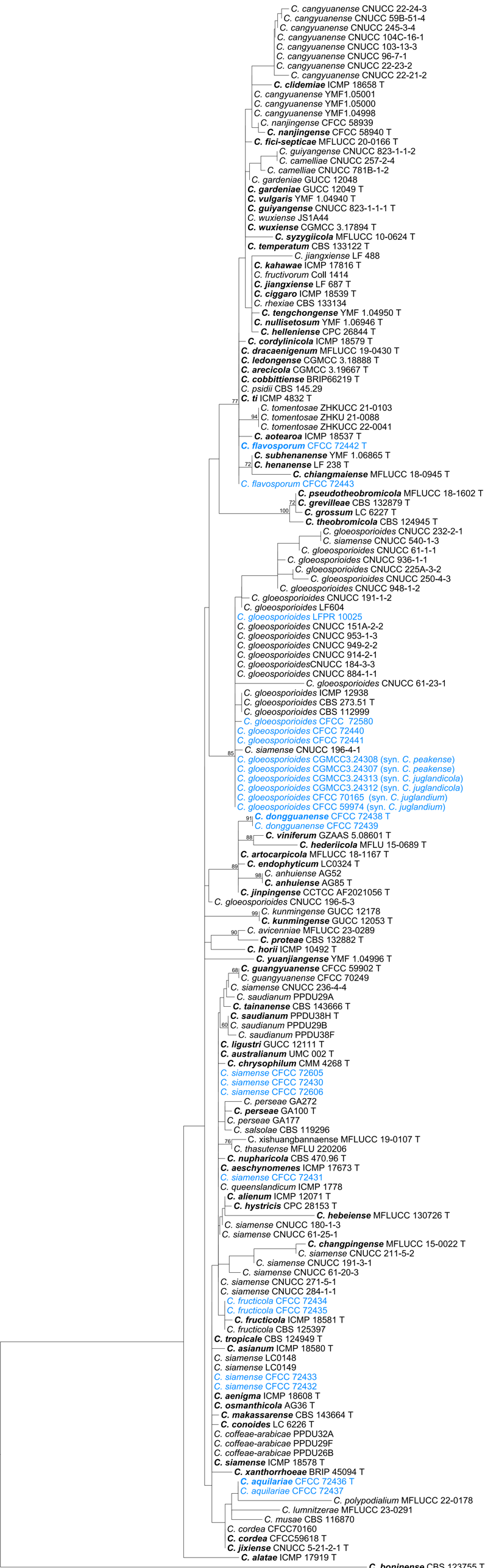

Supplement: Supplementary file 1 [file jof-11-00781-s001.zip › Figure S22.PDF]

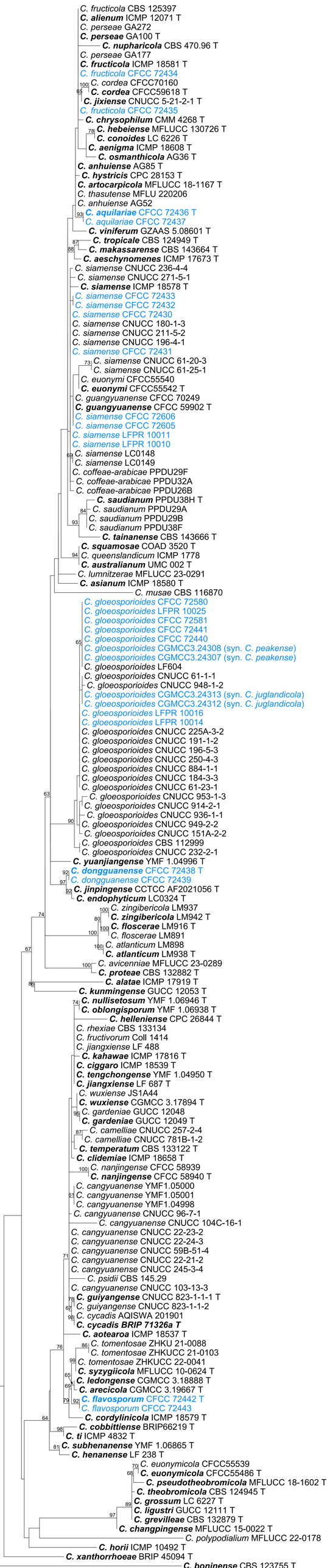

Supplement: Supplementary file 1 [file jof-11-00781-s001.zip › Figure S23.PDF]

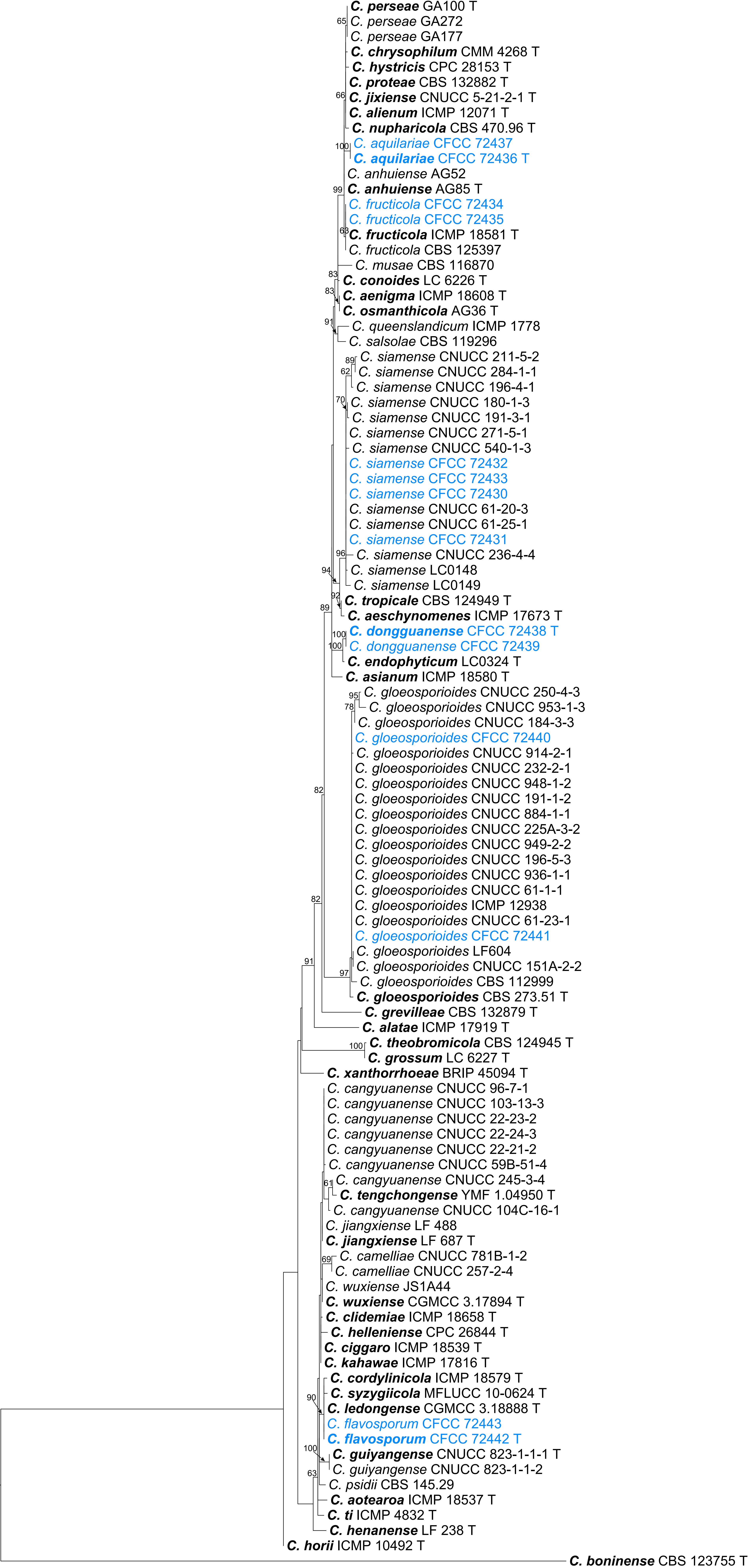

Supplement: Supplementary file 1 [file jof-11-00781-s001.zip › Figure S24.PDF]

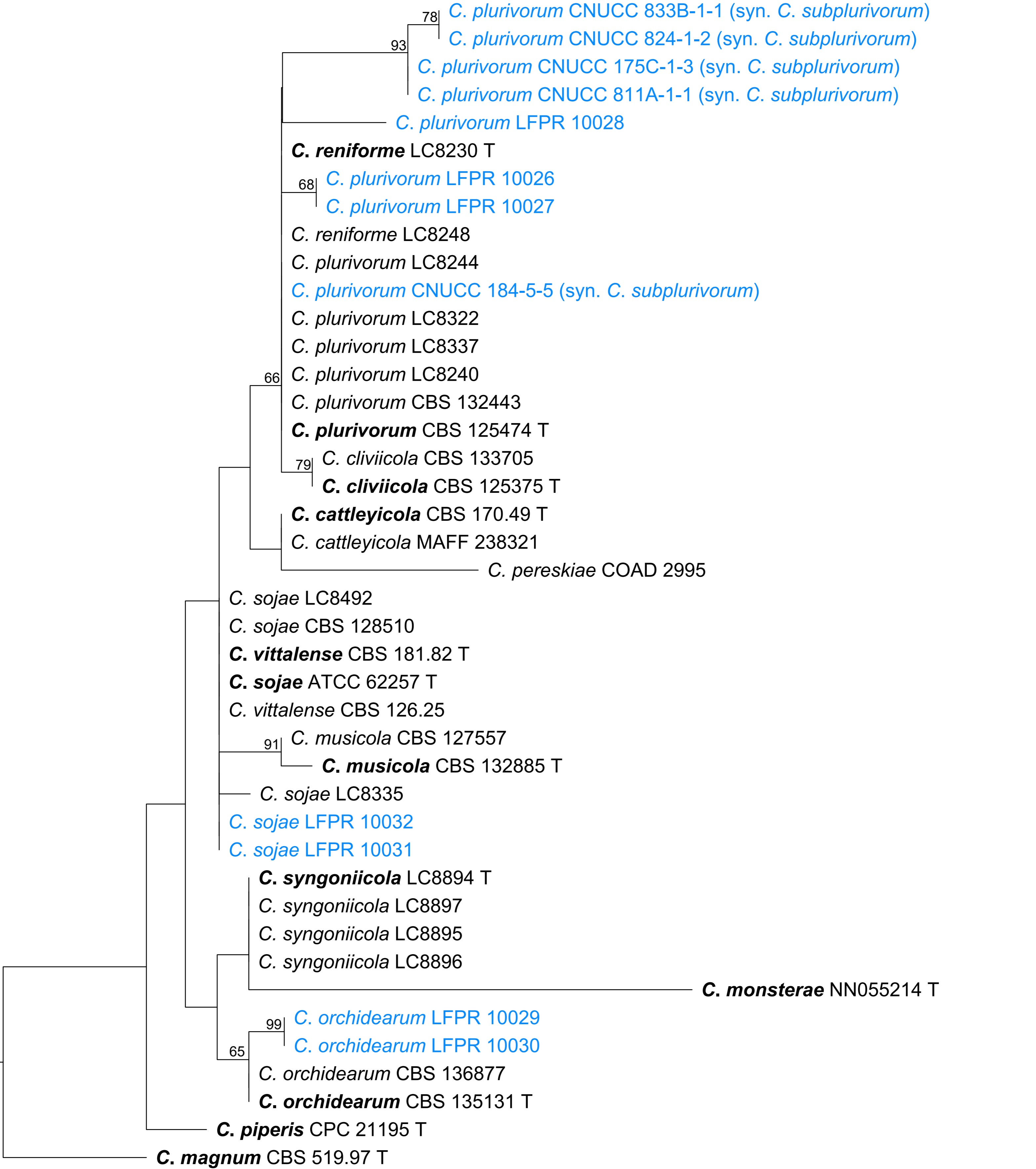

Supplement: Supplementary file 1 [file jof-11-00781-s001.zip › Figure S25.pdf]

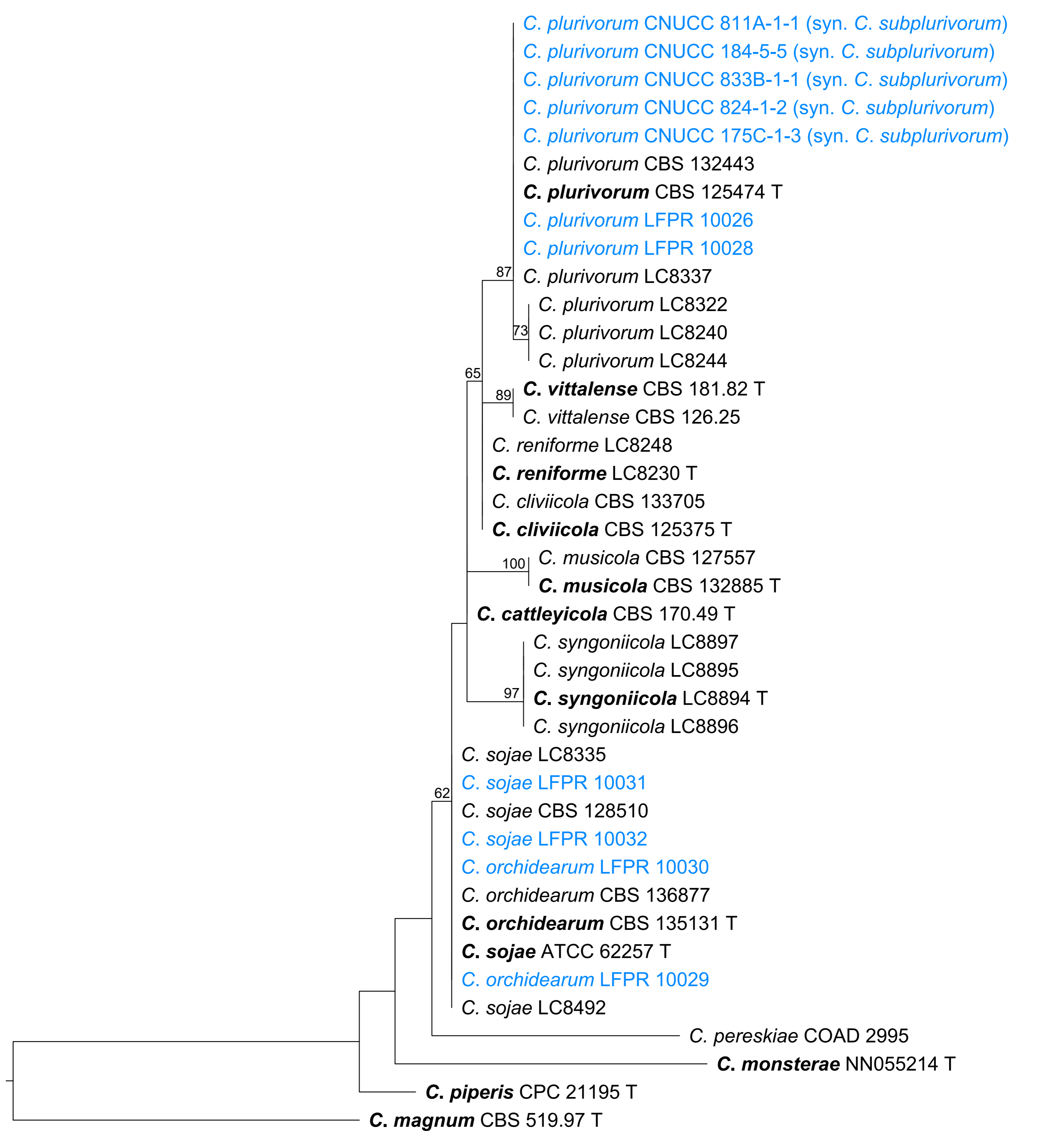

Supplement: Supplementary file 1 [file jof-11-00781-s001.zip › Figure S26.pdf]

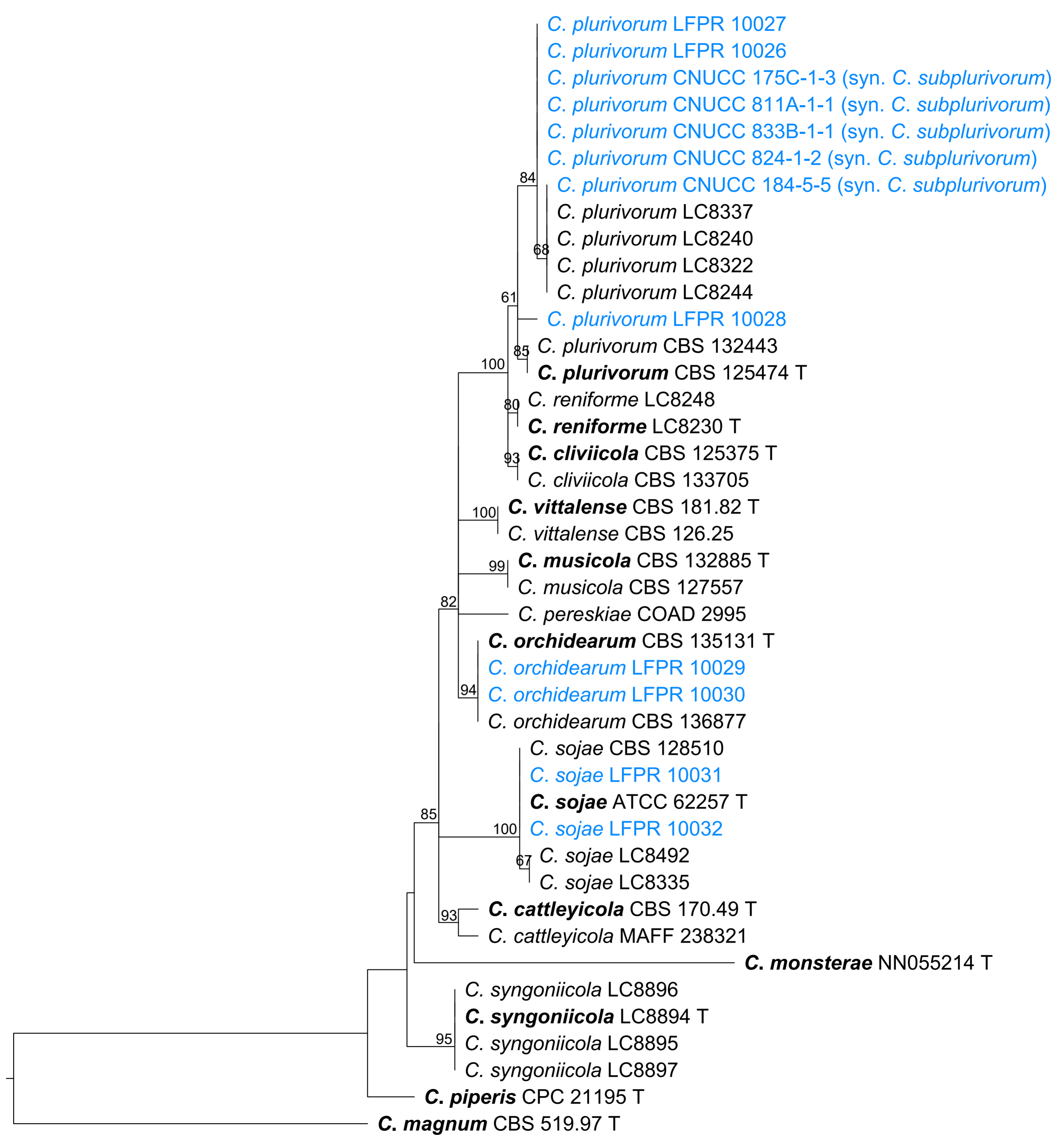

Supplement: Supplementary file 1 [file jof-11-00781-s001.zip › Figure S27.PDF]

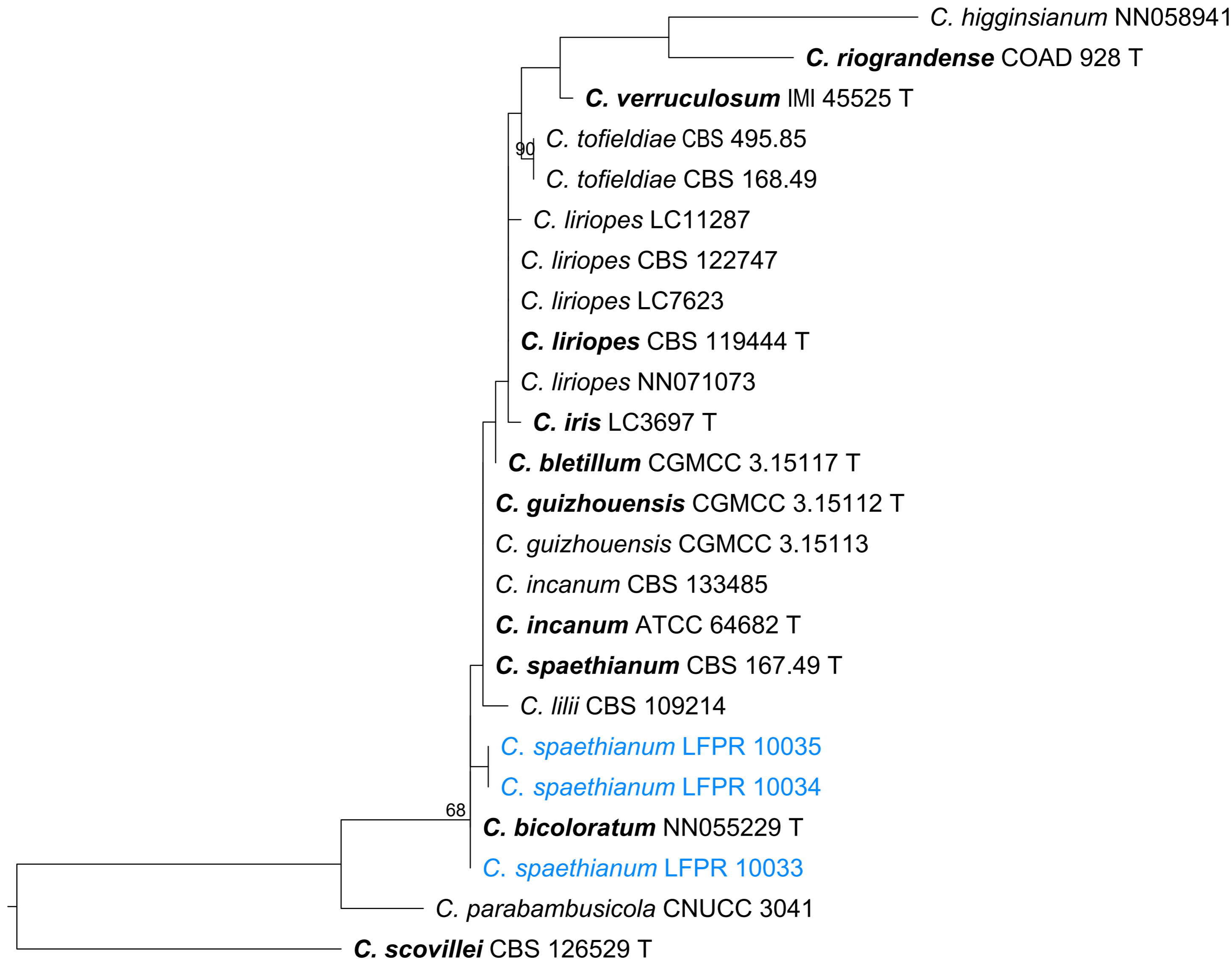

Supplement: Supplementary file 1 [file jof-11-00781-s001.zip › Figure S28.pdf]

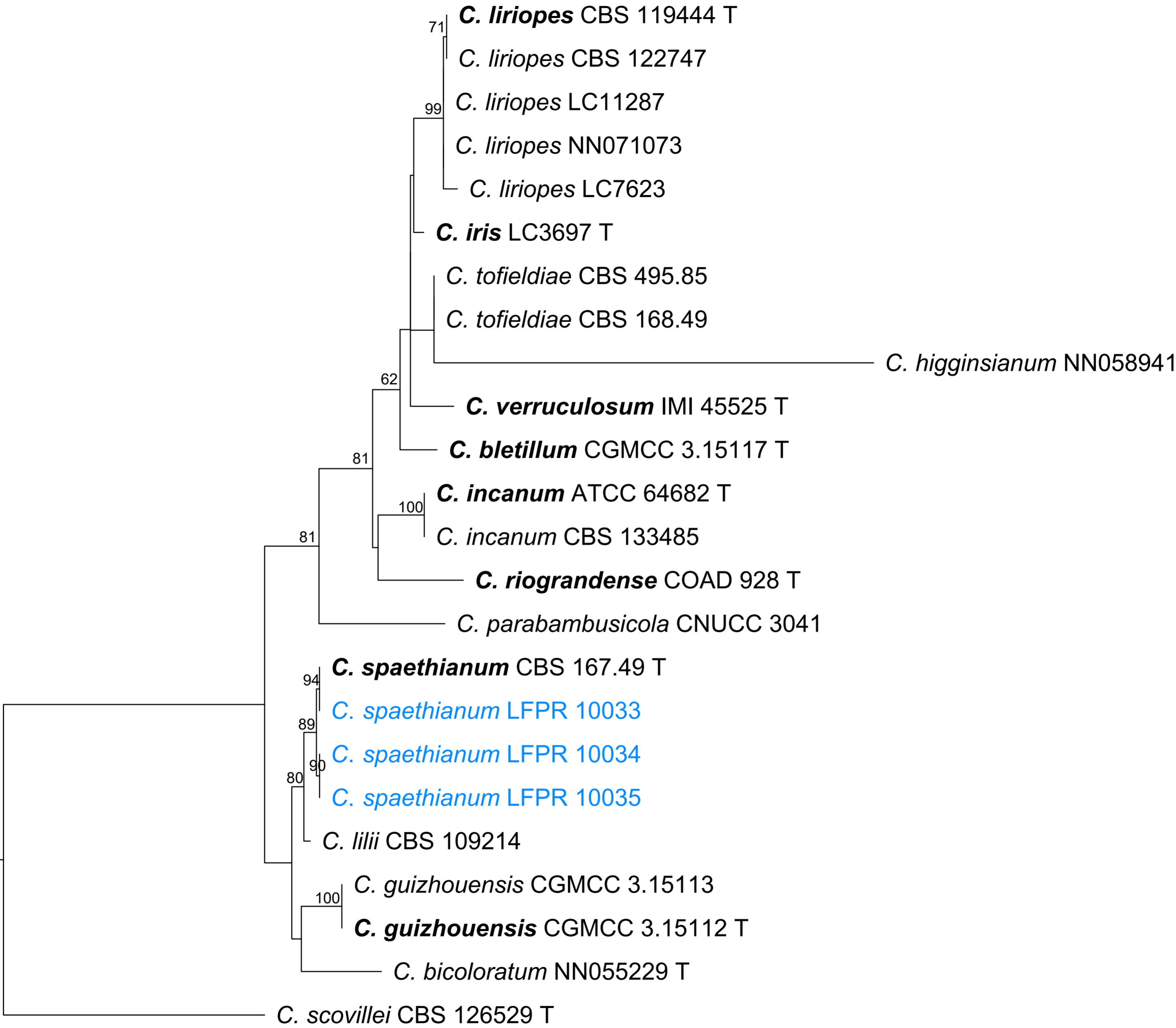

0.09

Supplement: Supplementary file 1 [file jof-11-00781-s001.zip › Figure S29.pdf]

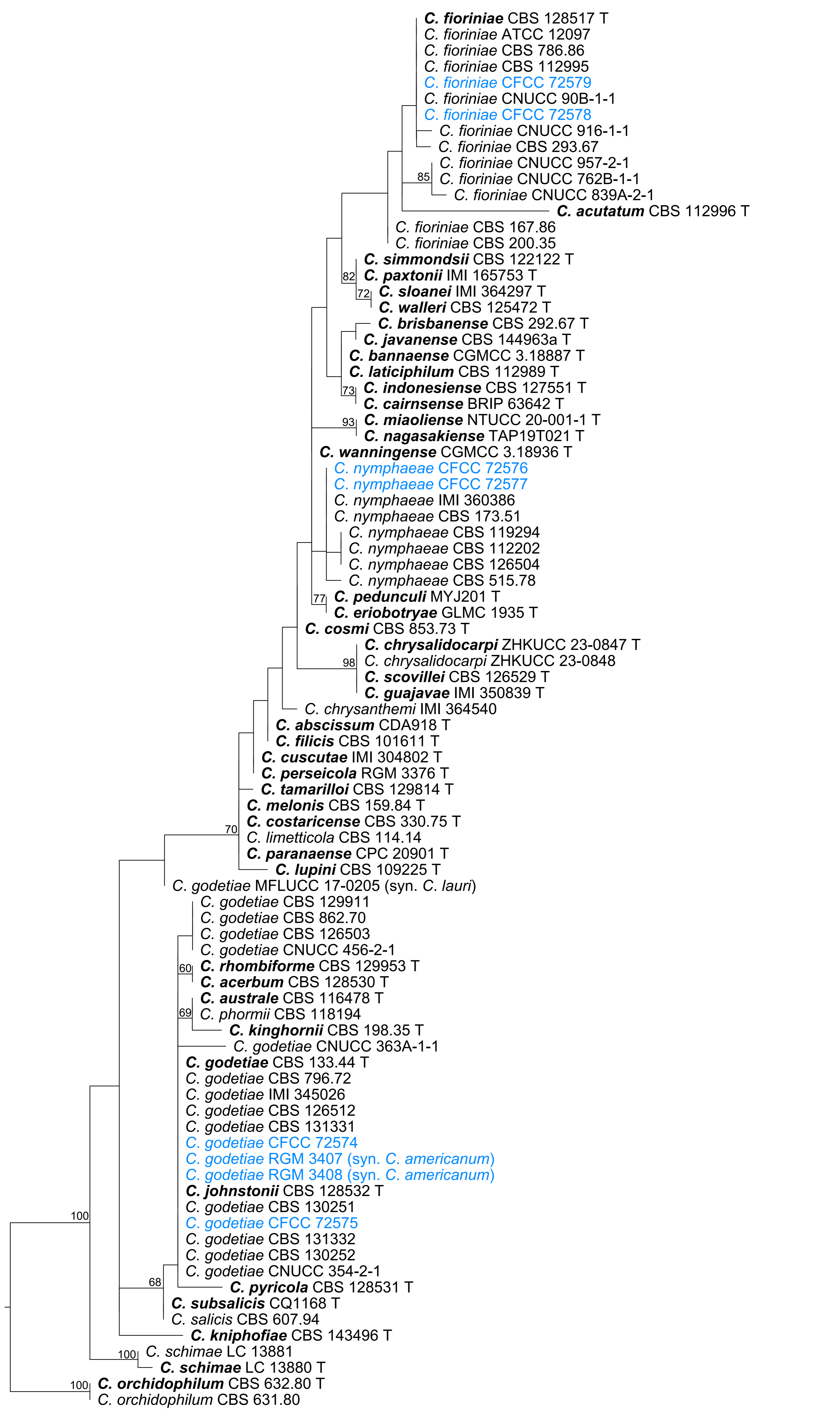

Supplement: Supplementary file 1 [file jof-11-00781-s001.zip › Figure S3.pdf]

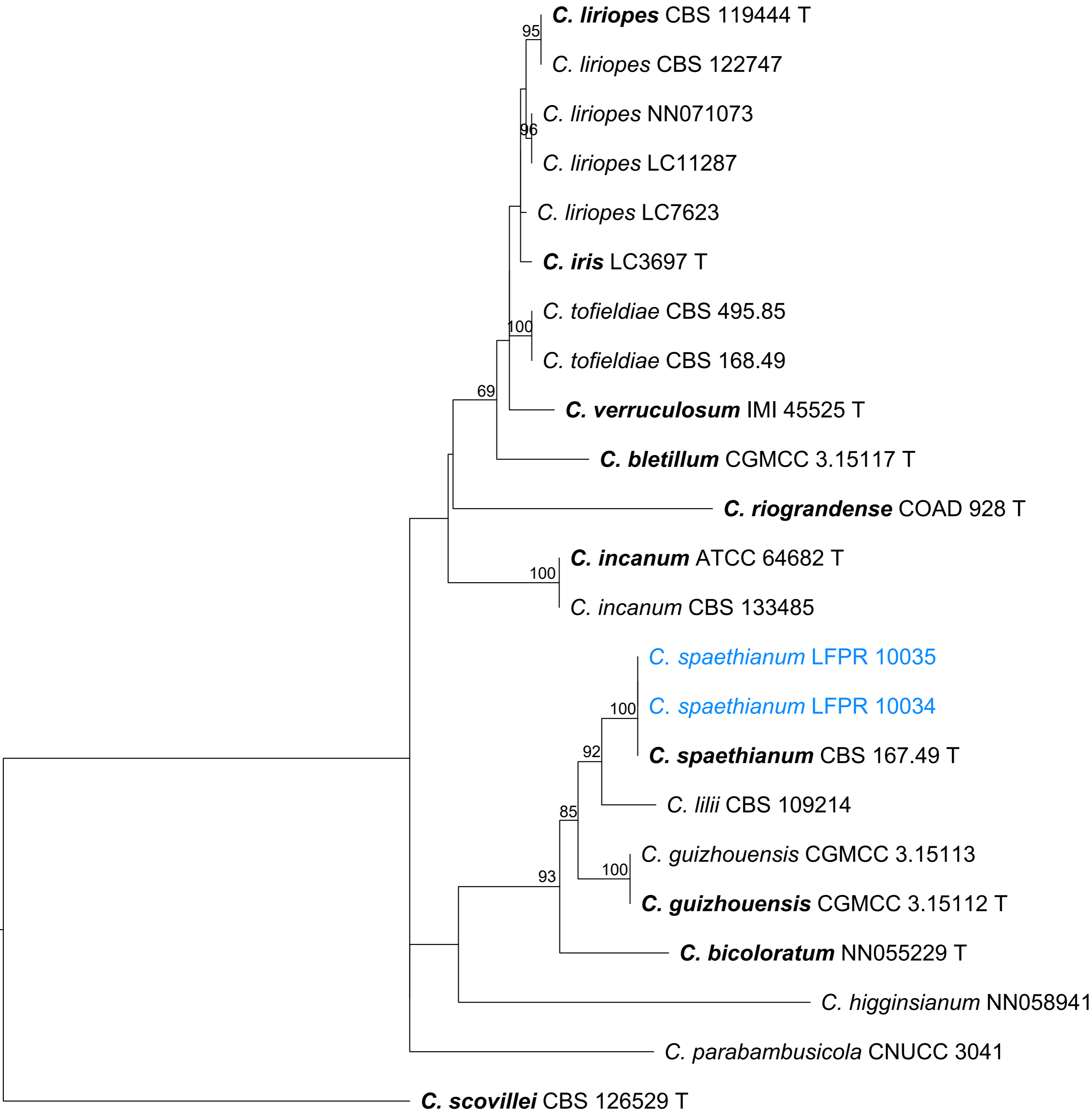

0.03

Supplement: Supplementary file 1 [file jof-11-00781-s001.zip › Figure S30.pdf]

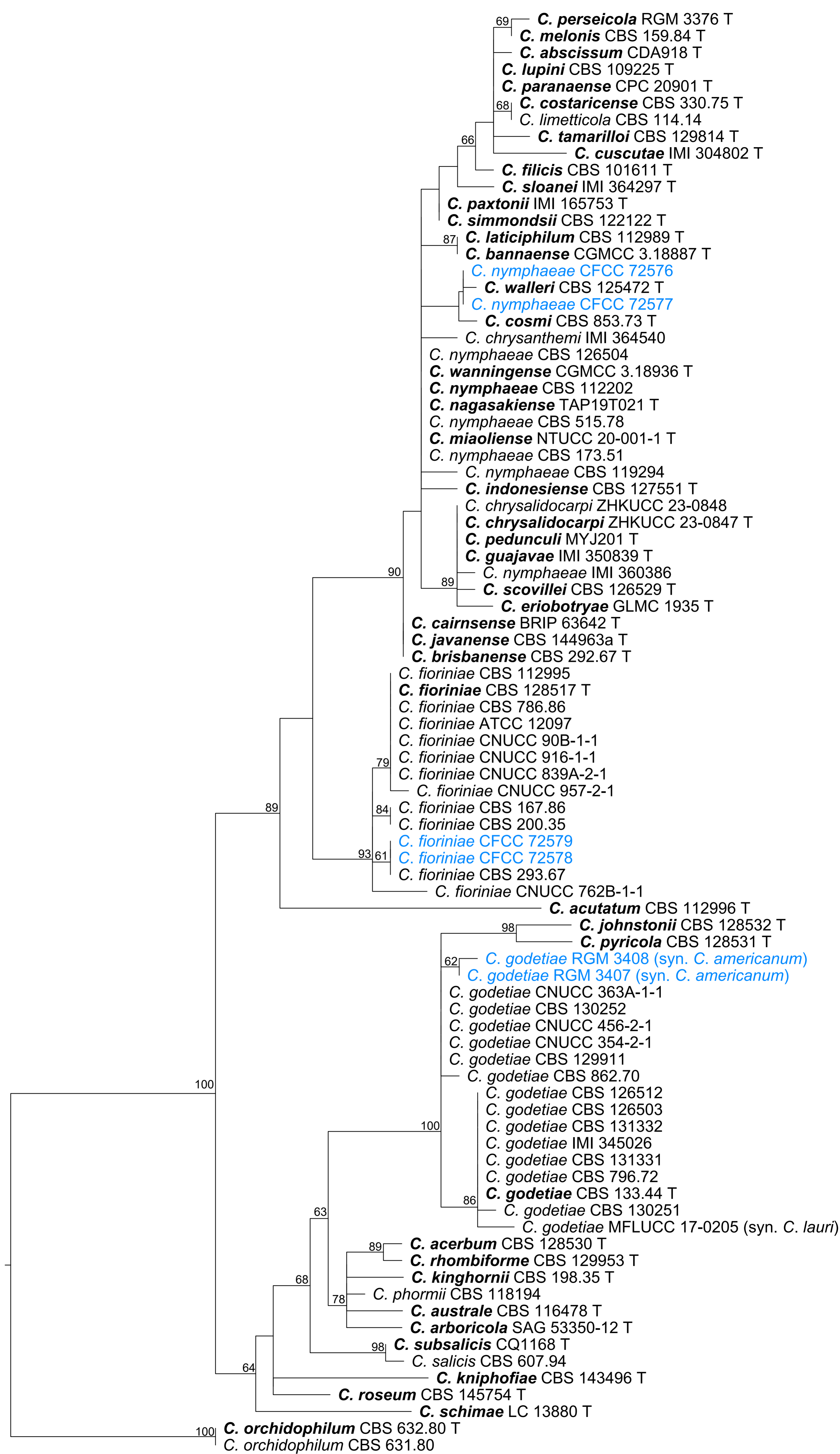

Supplement: Supplementary file 1 [file jof-11-00781-s001.zip › Figure S4.pdf]

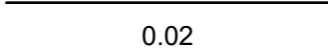

Supplement: Supplementary file 1 [file jof-11-00781-s001.zip › Figure S5.pdf]

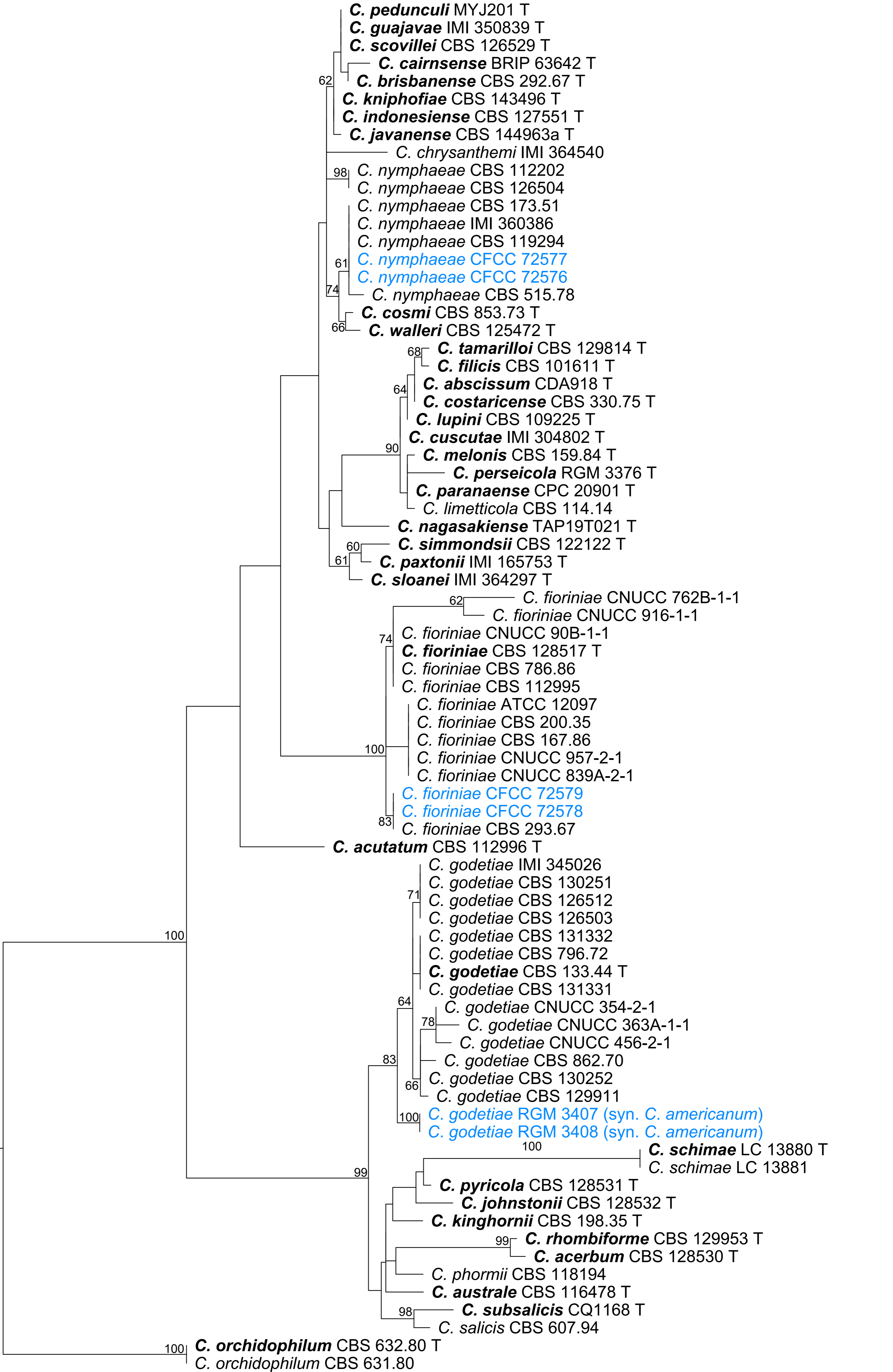

Supplement: Supplementary file 1 [file jof-11-00781-s001.zip › Figure S6.pdf]

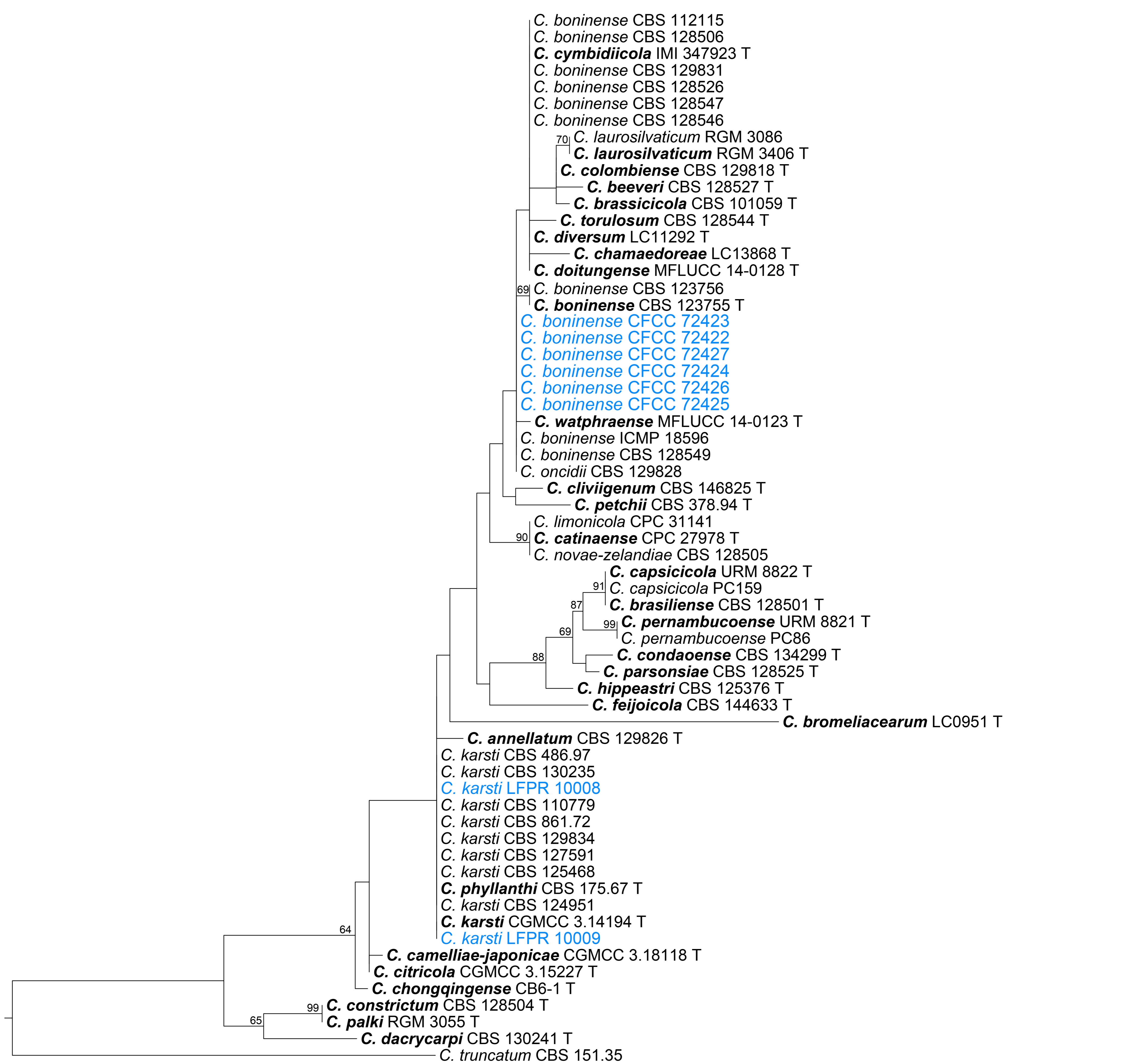

Supplement: Supplementary file 1 [file jof-11-00781-s001.zip › Figure S7.PDF]

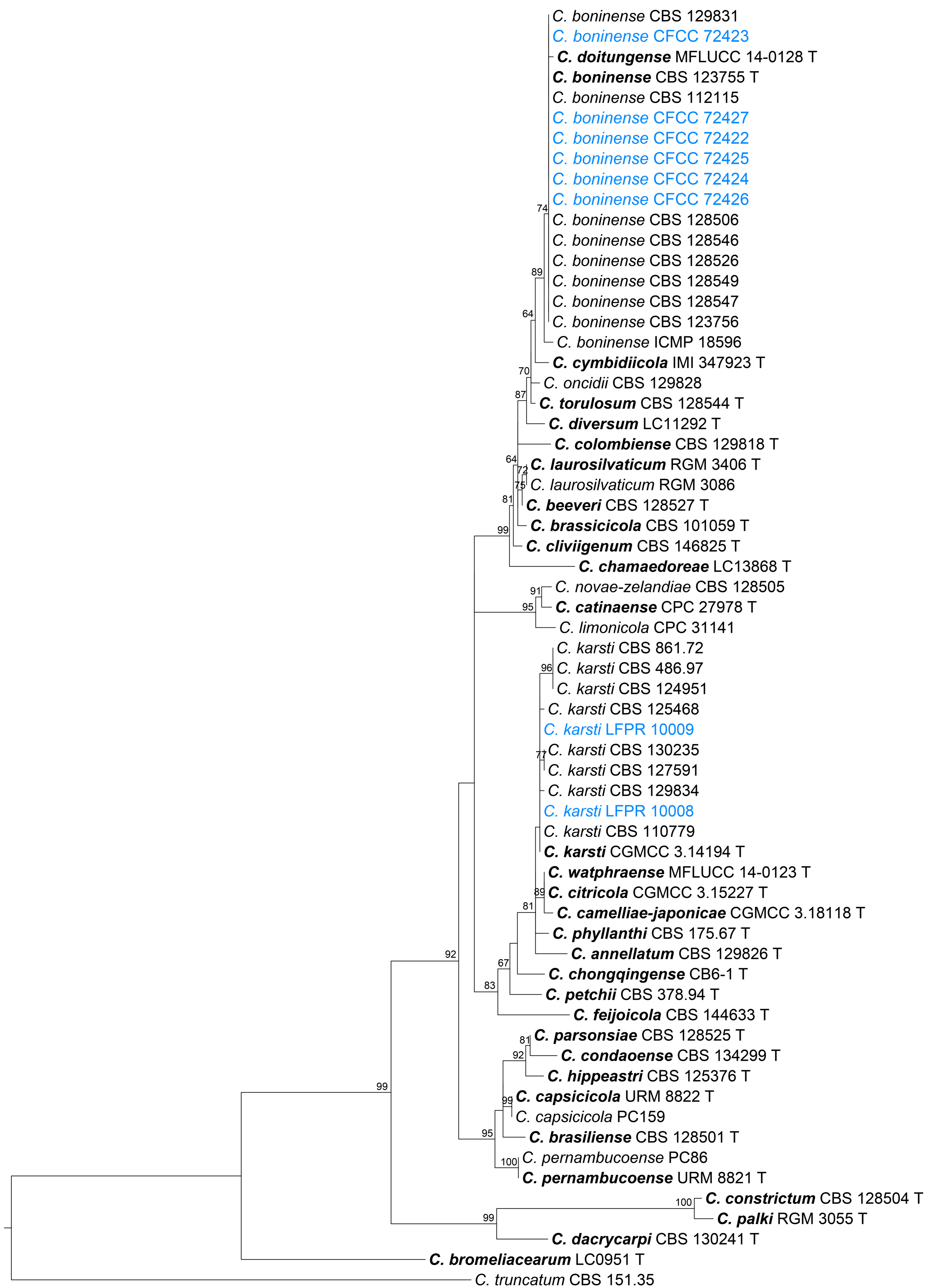

Supplement: Supplementary file 1 [file jof-11-00781-s001.zip › Figure S8.PDF]

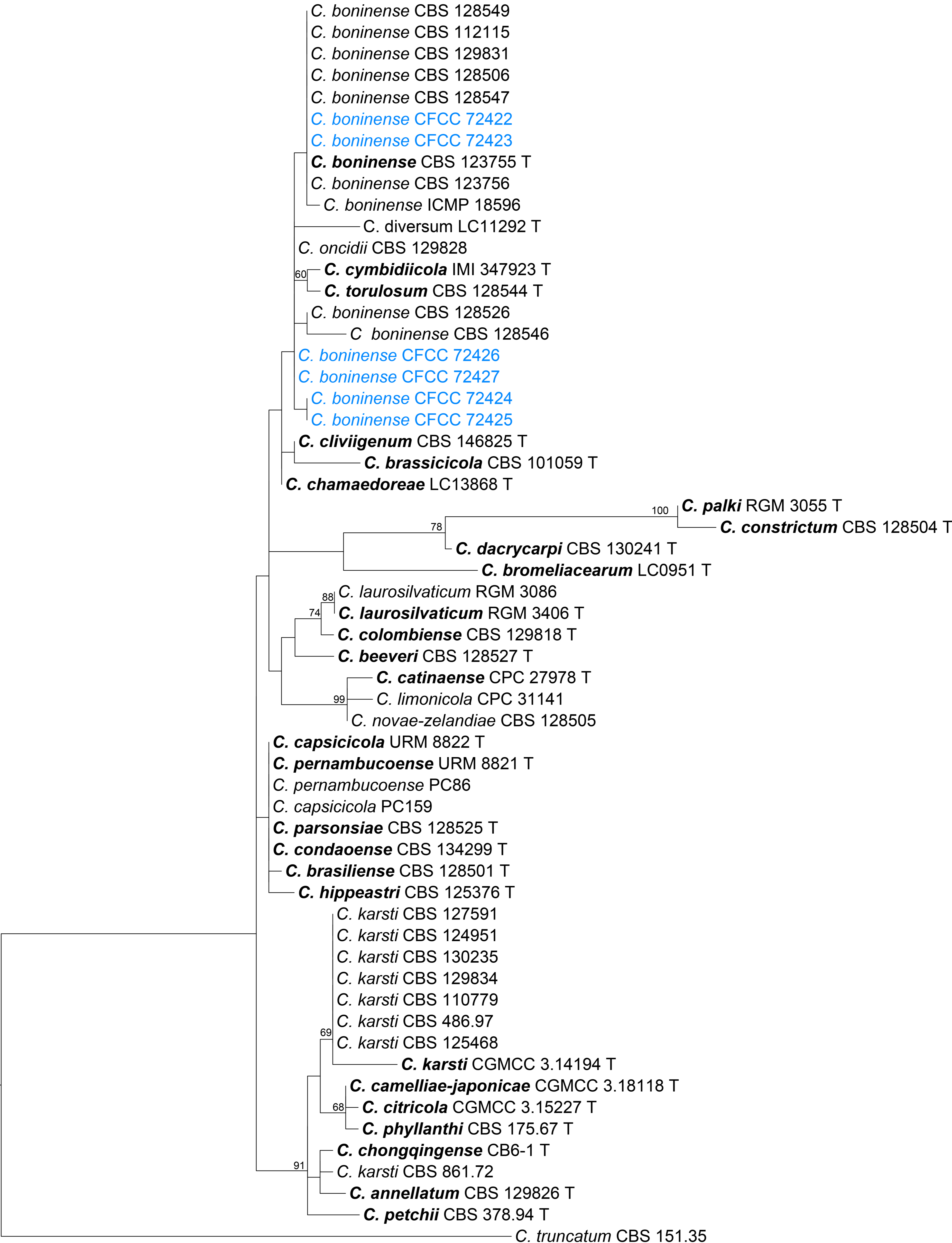

Supplement: Supplementary file 1 [file jof-11-00781-s001.zip › Figure S9.PDF]
